# Antagonistic roles of histone H3 lysine methyltransferases dictate tumor immune surveillance

**DOI:** 10.1038/s41467-026-76657-3

**Published:** 2026-09-14

**Authors:** Amy C. Gladstein, Akino Mercy Charles Solomon, Carson D. Poltorack, Sharan Venkatesh, Keren M. Adler, Maggie R. Robertson, Stephanie Stransky, Valerie M. Irizarry-Negron, Dain A. Ruiz, Dingwen Tao, Nelson F. Freeburg, Simone Sidoli, Irfan A. Asangani, Sydney M. Shaffer, David M. Feldser

**Affiliations:** 1 Department of Cancer Biology, Perelman School of Medicine, University of Pennsylvania, Philadelphia, PA USA; 2 Cell and Molecular Biology Graduate Program, Perelman School of Medicine, University of Pennsylvania, Philadelphia, PA USA; 3 Department of Bioengineering, Perelman School of Medicine, University of Pennsylvania, Philadelphia, PA USA; 4 Department of Pathology and Laboratory Medicine, Perelman School of Medicine, University of Pennsylvania, Philadelphia, PA USA; 5 Bioengineering Graduate Group, School of Engineering and Applied Science, University of Pennsylvania, Philadelphia, PA USA; 6 Department of Biochemistry, Albert Einstein College of Medicine, New York, NY USA; 7 Abramson Family Cancer Research Institute, University of Pennsylvania, Philadelphia, PA USA

**Keywords:** Tumour immunology, Non-small-cell lung cancer, Cancer epigenetics

## Abstract

SETD2 is a critical tumor suppressor in lung cancer that catalyzes histone H3 lysine 36 (H3K36) trimethylation. Oncogenic histone H3 alleles, including *H3*^*K36M*^, causally drive cancer formation by dominantly inhibiting SETD2 and other H3K36 methyltransferases. Here we show that expression of the *H3*^*K36M*^ oncogene paradoxically suppresses tumor growth in KRAS-driven lung cancer by promoting transcription of repetitive elements and accumulation of cytoplasmic dsRNA, activating antiviral-like immune clearance. Unexpectedly, all attendant effects of *H3*^*K36M*^ expression require SETD2 activity despite widespread loss of H3K36 trimethylation. Reducing H3K36 dimethylation through genetic or pharmacologic attenuation of NSD2 phenocopies H3^K36M^ expression, revealing NSD2 as the relevant target through which H3^K36M^ triggers immune activation. Mechanistically, H3^K36M^ expression or NSD2 attenuation reveals a SETD2-dependent mechanism that limits EZH2-mediated repression of repetitive elements, enabling dsRNA accumulation and immune activation. These findings establish opposing roles for NSD2- and SETD2-dependent H3K36 methylation in lung tumor immunity and reveal an NSD2–SETD2–EZH2 axis that can be targeted to modulate viral mimicry and immune surveillance in cancer.

## Abstract (web summary)

Histone H3K36 methyltransferases are frequently altered in cancer, yet the distinct roles of H3K36 methylation states in tumorigenesis and immunity remain unclear. This study identifies an H3K36-dependent epigenetic circuit that controls endogenous retroviral elements (ERVs) repression and immune surveillance in KRAS-driven lung cancer models.

## Introduction

Histone H3 lysine 36 (H3K36) methylation is a critical post-translational modification that regulates chromatin organization, genomic stability, transcriptional fidelity, and cell identity^1,2^. Alterations in H3K36 methyltransferases occur across diverse cancer types, but the functional consequences of disrupting distinct H3K36 methylation states remain incompletely understood^3^. H3K36 can be mono-, di-, or tri-methylated, with each methylation state exhibiting distinct chromatin localization and biological functions^1^. In mammalian cells, multiple enzymes, principally NSD1 and NSD2, catalyze H3K36 mono- and di-methylation, whereas SETD2 is the sole enzyme responsible for H3K36 trimethylation^1^.

SETD2 is frequently inactivated in malignancies including clear cell renal cell carcinoma, gliomas, and KRAS-driven lung adenocarcinoma^3^. In KRAS-driven lung adenocarcinoma, SETD2 acts as a potent tumor suppressor, and its inactivation accelerates tumor growth^4–7^. SETD2 binds RNA polymerase II and promotes co-transcriptional deposition of H3K36me3^8,9^. Although H3K36me3 marks actively transcribed gene bodies, it also contributes to restoration of chromatin after transcription and regulates DNA repair, alternative splicing, and RNA processing^10–15^. More recently, SETD2 has also been reported to methylate non-histone substrates, playing critical roles in cytoskeletal remodeling (α-tubulin and actin), interferon signaling (STAT1), and transcriptional repression (EZH2), raising the possibility that SETD2 loss can affect tumor biology through multiple mechanisms^16–19^.

In contrast to SETD2, the H3K36 di-methyltransferases NSD1 and NSD2 are frequently overexpressed in cancer^20^. NSD2 promotes tumor progression in KRAS-driven lung adenocarcinoma, but the mechanisms by which NSD2-dependent H3K36me2 supports tumor growth remain poorly understood^21^. H3K36me2 is enriched across intergenic regions and has established roles in DNA repair and DNA methylation^22,23^. Importantly, both H3K36me2 and H3K36me3 can antagonize EZH2, the catalytic component of PRC2 that deposits the transcriptionally repressive mark H3K27me3^24,25^. Thus, cancer-associated perturbations in H3K36 methylation may affect tumor behavior not only by altering H3K36 methylation itself, but also by reshaping the balance between H3K36- and H3K27-marked chromatin states.

H3K36 methylation can also be disrupted by mutations in histone H3.1 and H3.3 genes. Lysine 36 mutations occur in nearly all chondroblastomas and are also found in undifferentiated sarcomas and head and neck squamous cell carcinomas^26–29^. These mutations have been termed “oncohistones” because they are causally linked to cancer, are expressed alongside wild-type H3 alleles, and dominantly inhibit H3K36 methylation both locally, where mutant histones are incorporated, and more broadly through inhibition of H3K36 methyltransferases^30,31^. This biology creates an apparent paradox in lung adenocarcinoma: although both *SETD2* loss and *H3*^*K36M*^ oncohistone expression disrupt H3K36 methylation, *SETD2*-inactivating mutations are recurrently selected in lung adenocarcinoma, whereas *H3*^*K36M*^ and other H3 oncohistone mutations are largely absent from this disease^28^.

Here, we used *H3*^*K36M*^ expression as a genetic tool to interrogate how distinct H3K36 methylation states influence KRAS-driven lung adenocarcinoma. Contrary to the expectation that *H3*^*K36M*^ expression would phenocopy *Setd2* loss, we find that H3^K36M^ suppresses tumor growth through a *Setd2*-dependent, immune-mediated mechanism. Our data identify opposing roles for NSD2- and SETD2-dependent H3K36 methylation in regulating endogenous retroviral element expression, dsRNA accumulation, and antiviral-like tumor immune surveillance. They further reveal that, in the absence of SETD2, EZH2-dependent H3K27me3 compensates by silencing retroelements, thereby preventing dsRNA accumulation and immune activation. Together, these findings define an NSD2–SETD2–EZH2 axis that links chromatin regulation to immune evasion and reveals epigenetic vulnerabilities in KRAS-driven lung cancer.

## Results

### H3^K36M^ limits KRAS-driven tumor growth in a Setd2-dependent manner

**Fig. 1:**
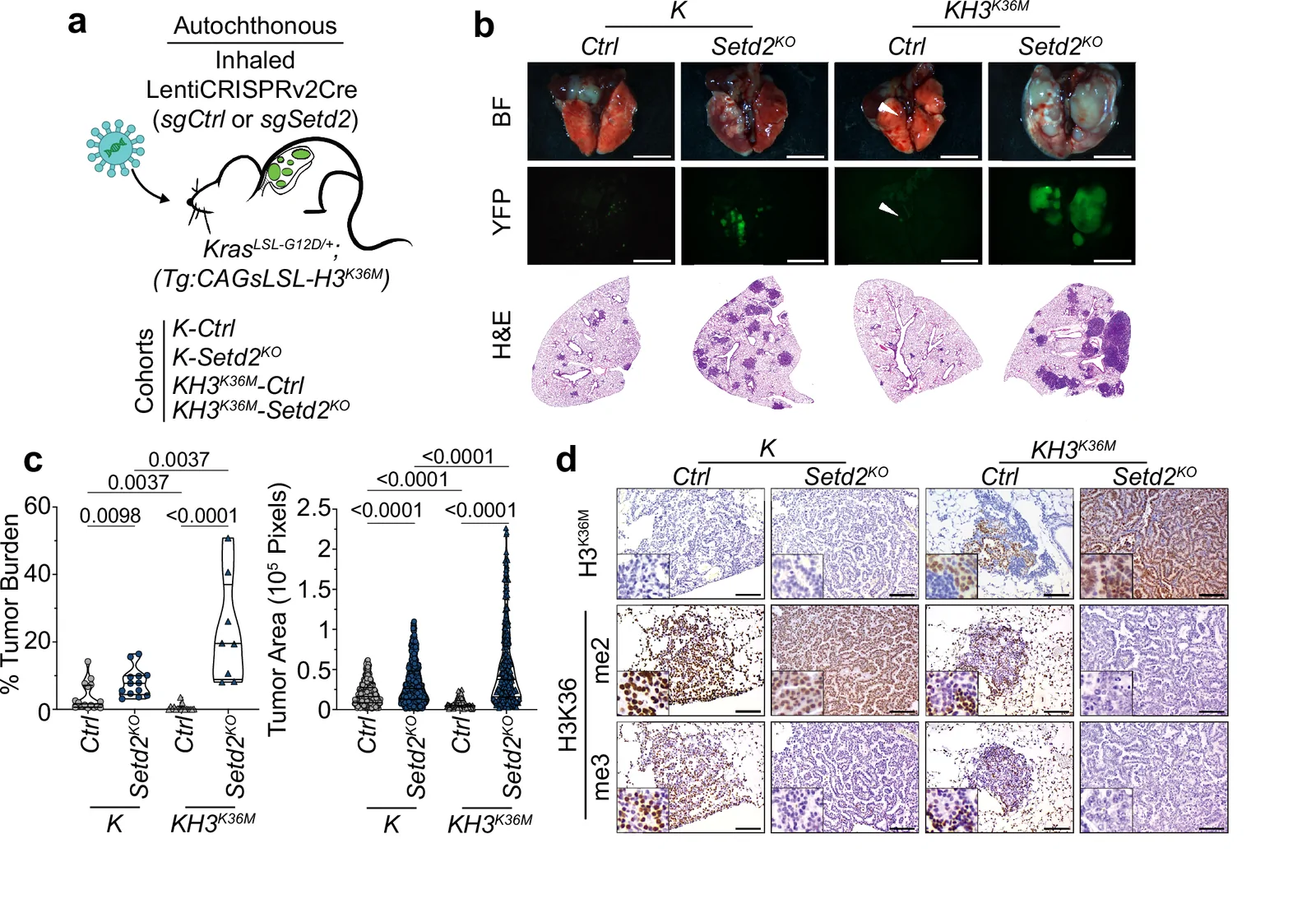
H3^K36M^ limits Kras-driven tumor growth in a Setd2-dependent manner. **a** Experimental scheme of autochthonous tumor initiation. **b** Brightfield (BF) and fluorescent micrographs of lungs from *K-Ctrl*, *K-Setd2*^*KO*^, *KH3*^*K36M*^*-Ctrl*, and *KH3*^*K36M*^*-Setd2*^*KO*^ mice. Scale bars are 4.4 mm. Below are representative H&E images of tumor-bearing lobes from *K-Ctrl*, *K-Setd2*^*KO*^, *KH3*^*K36M*^*-Ctrl*, and *KH3*^*K36M*^*-Setd2*^*KO*^ mice. This experiment was independently repeated three times with similar results. **c** Quantification of tumor burden and individual tumor area in *K-Ctrl* (*n* = 16 mice), *K-Setd2*^*KO*^ (*n* = 13 mice), *KH3*^*K36M*^*-Ctrl* (*n* = 17 mice), and *KH3*^*K36M*^*-Setd2*^*KO*^ (*n* = 8 mice) mice. Significance was determined by two-sided unpaired Student’s *t*test. Violin plots show the data distribution, with the center line indicating the median and the upper and lower lines indicating the interquartile range. **d** IHC for H3^K36M^, H3K36me2, and H3K36me3 in *K-Ctrl*, *K-Setd2*^*KO*^, *KH3*^*K36M*^*-Ctrl*, and *KH3*^*K36M*^*-Setd2*^*KO*^ mice. Scale bars are 119 μm; insets are magnified x3. This IHC was independently repeated three times with similar results.

To determine how H3^K36M^ expression affects KRAS-driven lung adenocarcinoma, we expressed H3^K36M^ in an autochthonous *Kras*^*G12D*^-driven mouse model. *Kras*^*LSL-G12D/+*^*;Rosa26*^*LSL-YFP/+*^ (*K*(*Y*)) and *Kras*^*LSL-G12D/+*^;*Rosa26*^*LSL-YFP*^/+;*Tg::CAG-LSL-H3*^*K36M*^ (*K*(*Y*)*H3*^*K36M*^) mice were transduced with a lentiviral vector expressing Cre, Cas9, and either a *Setd2*-targeting or inert non-targeting sgRNA. This generated four experimental cohorts: *K-Ctrl*, *K-Setd2*^*KO*^, *KH3*^*K36M*^*-Ctrl*, and *KH3*^*K36M*^*-Setd2*^*KO*^ (Fig. 1a). As expected, *Setd2* inactivation significantly increased tumor growth compared to *K-Ctrl* tumors (Fig. 1b, c and Supplementary Fig. 1a). However, in stark contrast, *H3*^*K36M*^ expression significantly reduced tumor number, overall tumor burden, and individual tumor size. Most surprisingly, combined *H3*^*K36M*^ expression and *Setd2* inactivation restored tumor formation and growth, indicating that the tumor-suppressive effect of H3^K36M^ is *Setd2*-dependent.

H3^K36M^ expression was readily detected in both *KH3*^*K36M*^*-Ctrl* and *KH3*^*K36M*^-*Setd2*^KO^ tumors (Fig. 1d). The detection of H3^K36M^-positive tumor cells in both cohorts indicates that the restored tumor growth observed after *Setd2* inactivation was not explained by loss of *H3*^*K36M*^ transgene expression. As expected, H3K36me3 was reduced in *K-Setd2*^*KO*^, *KH3*^*K36M*^*-Ctrl*, and *KH3*^*K36M*^*-Setd2*^*KO*^ tumors, whereas H3K36me2 was reduced in *KH3*^*K36M*^*-Ctrl* and *KH3*^*K36M*^*-Setd2*^*KO*^ tumors (Fig. 1d). Consistent with the tumor-burden phenotype, *KH3*^*K36M*^*-Ctrl* tumors had significantly increased cell death, whereas *KH3*^*K36M*^*-Setd2*^*KO*^ tumors had increased tumor-cell proliferation relative to the other tumor genotypes (Supplementary Fig. 1b–e). All tumors retained typical lung adenocarcinoma lineage markers (Supplementary Fig. 1f). Thus, despite broadly inhibiting H3K36 methylation, H3^K36M^ does not phenocopy *Setd2* inactivation in KRAS-driven lung adenocarcinoma. Instead, H3^K36M^ suppresses tumor formation and growth through a *Setd2*-dependent mechanism.

### H3^K36M^ promotes a Setd2-dependent tumor-cell-killing immune response

**Fig. 2:**
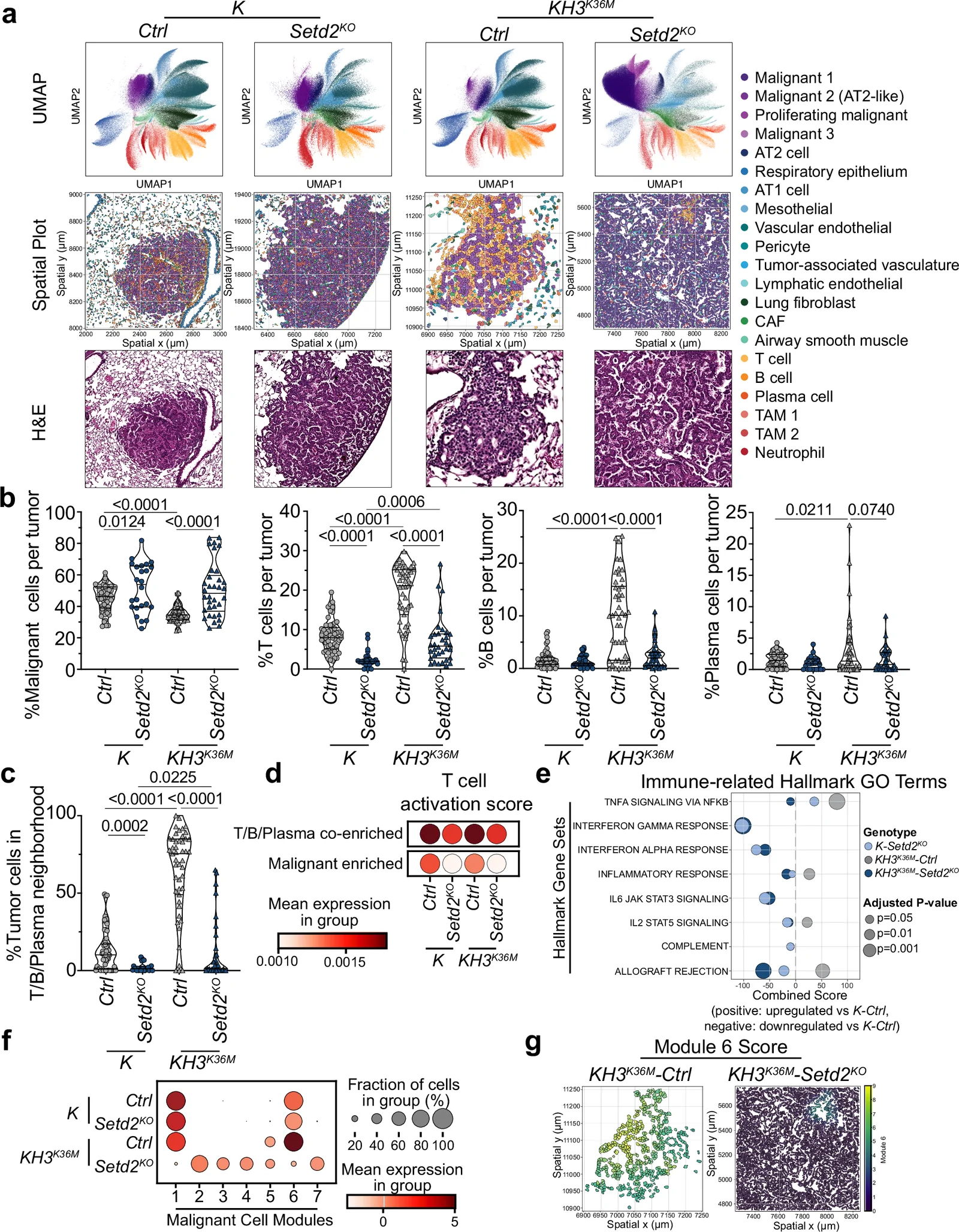
H3^K36M^ promotes a Setd2-dependent tumor-cell-killing immune response. **a** UMAP embeddings of cells from *K-Ctrl*, *K-Setd2*^*KO*^, *KH3*^*K36M*^*-Ctrl*, and *KH3*^*K36M*^*-Setd2*^*KO*^ lung lobes, paired with representative single-tumor spatial plots and H&E images. H&E images are on the same scale as the spatial plots. No independent repetitions of this H&E staining were conducted. **b** Percentage of cells within individual tumors identified as malignant, T, B, or plasma cells in cell typing of *K-Ctrl* (*n* = 54 tumors), *K-Setd2*^*KO*^(*n* = 24 tumors), *KH3*^*K36M*^*-Ctrl* (*n* = 47 tumors), and *KH3*^*K36M*^*-Setd2*^*KO*^ (*n* = 34 tumors) lung lobes. Significance was determined by two-sided unpaired Student’s *t*test. Violin plots show the data distribution, with the center line indicating the median and the upper and lower lines indicating the interquartile range. **c** Percentage of cells within individual *K-Ctrl* (*n* = 54 tumors), *K-Setd2*^*KO*^(*n* = 24 tumors), *KH3*^*K36M*^*-Ctrl* (*n* = 47 tumors), and *KH3*^*K36M*^*-Setd2*^*KO*^ (*n* = 34 tumors) tumors belonging to the T/B/Plasma-enriched cellular neighborhood. Significance was determined by two-sided unpaired Student’s *t*test. Violin plots show the data distribution, with the center line indicating the median and the upper and lower lines indicating the interquartile range. **d** T cell activation scores for T cells present in the T/B/Plasma-enriched or Malignant-enriched cellular neighborhoods in *K-Ctrl*, *K-Setd2*^*KO*^, *KH3*^*K36M*^*-Ctrl*, and *KH3*^*K36M*^*-Setd2*^*KO*^ lung lobes. **e** Gene Ontology (GO) enrichment scores of differentially upregulated and downregulated genes in *K-Setd2*^*KO*^, *KH3*^*K36M*^*-Ctrl*, and *KH3*^*K36M*^*-Setd2*^*KO*^ tumors compared to *K-Ctrl* tumors. Enrichment for a GO Term in differentially upregulated genes is depicted on the positive x-axis, while enrichment in downregulated genes is depicted on the negative x-axis. Non-significant enrichments are not plotted. Differentially expressed genes were identified using a two-sided Student’s t-test implemented in Scanpy comparing each genotype with *K-Ctrl* malignant cells. *P* values were adjusted for multiple comparisons using the Benjamini–Hochberg false discovery rate correction. **f** Hotspot malignant cell module scores in *K-Ctrl*, *K-Setd2*^*KO*^, *KH3*^*K36M*^*-Ctrl*, and *KH3*^*K36M*^*-Setd2*^*KO*^ lung lobes. **g** Spatial plots of malignant cells colored by their expression of Hotspot Module 6 in representative *KH3*^*K36M*^*-Ctrl* and *KH3*^*K36M*^*-Setd2*^*KO*^ tumors.

Pathology review of *KH3*^*K36M*^*-Ctrl* tumors revealed prominent immune-cell infiltration that was largely absent from *KH3*^*K36M*^*-Setd2*^*KO*^ tumors (Supplementary Fig. 2a). To define the composition, activation state, and spatial organization of this response, we performed spatial transcriptomics using the Xenium 5000-plex pan-tissue and signaling pathway panel on lung lobes from *K-Ctrl*, *K-Setd2*^*KO*^, *KH3*^*K36M*^*-Ctrl*, and *KH3*^*K36M*^*-Setd2*^*KO*^ mice^32^. After quality control, we analyzed nearly 1.3 million single cells from 159 tumors and uncovered profound differences in the cellular composition, cellular states, and tissue organization of these tumors (Fig. 2a and Supplementary Fig. 3a–c).

Although *K-Ctrl* tumors appeared largely unremarkable by routine histopathology, spatial transcriptomic analysis revealed a low-level immune reaction that was diminished in *K-Setd2*^*KO*^ tumors. This immune reaction was markedly enhanced in *KH3*^*K36M*^*-Ctrl* tumors and was lost in *KH3*^*K36M*^*-Setd2*^*KO*^ tumors. Interestingly, the *KH3*^*K36M*^*-Ctrl* immune infiltrate was enriched for T cells, B cells, and plasma cells (Fig. 2b). Orthogonally, we sought to confirm these observations by staining tumors with an expanded panel of immune cell markers. *KH3*^*K36M*^*-Ctrl* tumors showed increased CD45-positive, CD3-positive, CD8-positive, B220-positive, and NKp46-positive cells relative to *K-Ctrl* tumors, and this immune infiltration was ablated in *KH3*^*K36M*^*-Setd2*^*KO*^ tumors (Supplementary Fig. 2b, c). Additional staining for myeloid and dendritic-cell markers showed that CD207/Langerin-positive cells were also increased in *KH3*^*K36M*^*-Ctrl* tumors, whereas CD11c, F4/80, and Ly6G staining did not show the same pattern (Supplementary Fig. 2b, c). These analyses indicate that *Setd2* inactivation suppresses both the low-level immune reaction present in KRAS-driven tumors and the robust lymphoid/NK-cell-dominated immune response associated with *H3*^*K36M*^ expression. Thus, *Setd2*-dependent tumor suppression in this setting is associated with maintenance of tumor immunogenicity and immune-mediated tumor-cell killing.

To identify spatial interactions between malignant and immune cell types across tumor genotypes, we performed cellular neighborhood analysis^33^ (Supplementary Fig. 3d–f). A cellular neighborhood enriched for T cells, B cells, and plasma cells was present in *K-Ctrl* tumors and was significantly enhanced in *KH3*^*K36M*^*-Ctrl* tumors (Fig. 2c). In both contexts, *Setd2* inactivation markedly reduced the abundance of this T/B/plasma-cell neighborhood. T cells within *K-Ctrl* and *KH3*^*K36M*^*-Ctrl* tumors also had higher T-cell activation scores than T cells in the corresponding *Setd2*^KO^ tumors, consistent with a more active anti-tumor immune response (Fig. 2d).

We next assessed malignant-cell gene expression programs across tumor genotypes. Gene Ontology (GO) terms associated with inflammatory signaling were suppressed by *Setd2* inactivation in both *K-Setd2*^*KO*^ and *KH3*^*K36M*^*-Setd2*^*KO*^ tumors, whereas a subset of these programs was enriched in *KH3*^*K36M*^*-Ctrl* tumors relative to *K-Ctrl* tumors (Fig. 2e). Conversely, gene expression programs associated with aggressive tumor growth were enriched in *KH3*^*K36M*^*-Setd2*^*KO*^ tumors (Supplementary Fig. 4a). Finally, to determine whether malignant-cell gene expression covaried spatially with immune infiltration, we performed Hotspot analysis on malignant cells^34^. Among the identified modules, Module 6 was elevated in *K-Ctrl* tumors, further increased in *KH3*^*K36M*^*-Ctrl* tumors, and reduced by *Setd2* inactivation in both contexts (Fig. 2f, g). Module 6 was composed largely of immune-related genes, including complement pathway components (*C3*, *Cfb*, *Cd55*), cytokine-associated genes (*Cxcl9*, *Il18rap*), and Jak/Stat pathway genes (*Stat1*, *Nfkb1*) (Supplementary Dataset 1). Module 6 expression also correlated with T-cell infiltration on a per-tumor basis, suggesting that this malignant-cell spatial program reflects local immune engagement (Supplementary Fig. 4b, c). Together, these spatial transcriptomic and immunostaining data show that KRAS-driven tumors harbor a low-level inflammatory immune reaction that is amplified by *H3*^*K36M*^ expression and suppressed by *Setd2* inactivation.

### NK cells are sufficient for H3^K36M^-promoted tumor cell killing

**Fig. 3:**
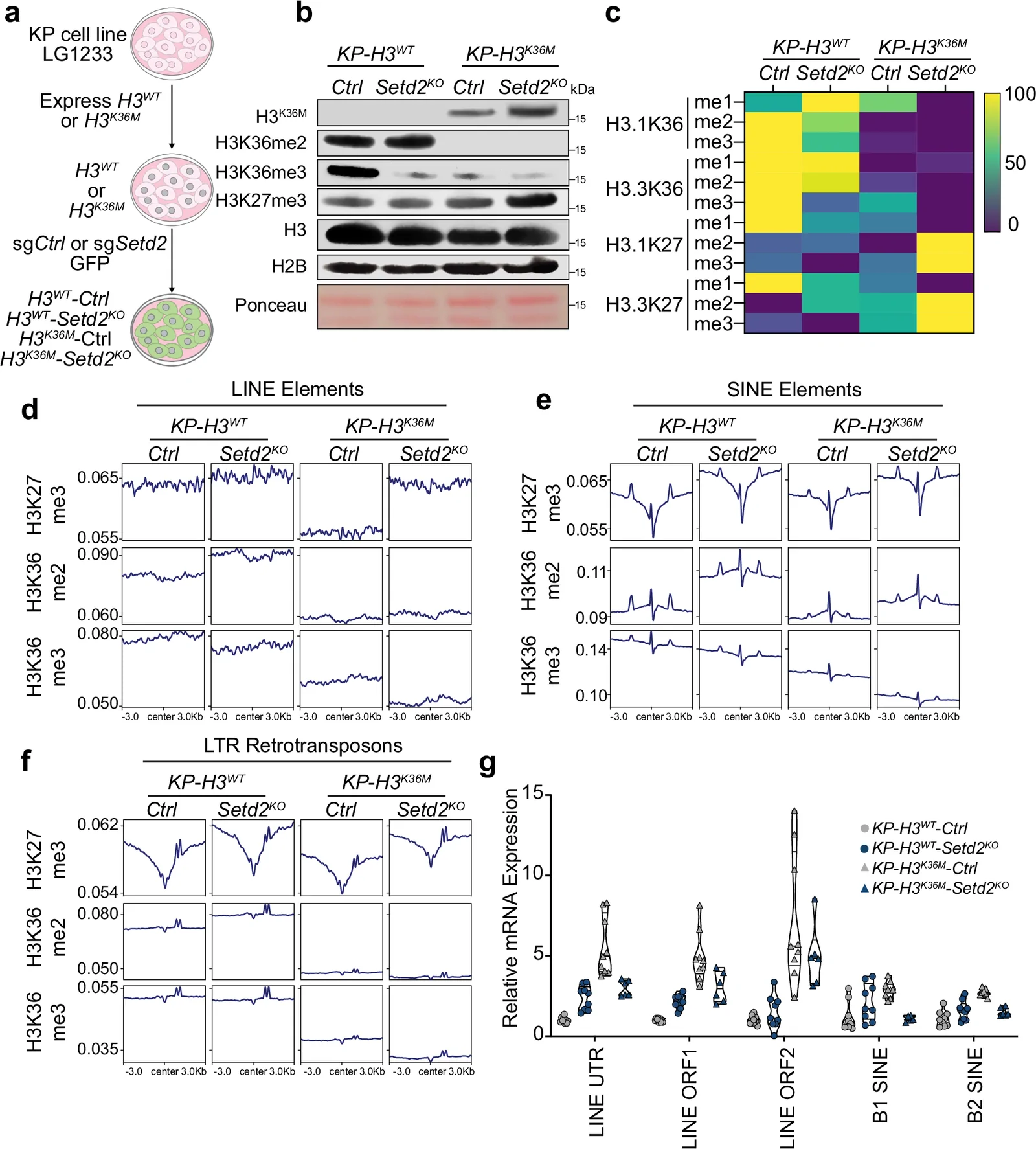
H3^K36M^ causes derepression of endogenous retroviral elements. **a** Schematic for generation of *KP-H3*^*WT*^*-Ctrl*, *KP-H3*^*WT*^*-Setd2*^*KO*^, *KP-H3*^*K36M*^*-Ctrl*, and *KP-H3*^*K36M*^*-Setd2*^*KO*^ cell lines. **b** Immunoblot analysis of H3^K36M^, H3K36me2, H3K36me3, H3K27me3, H3, and H2B in histones prepared from *KP-H3*^*WT*^*-Ctrl*, *KP-H3*^*WT*^*-Setd2*^*KO*^, *KP-H3*^*K36M*^*-Ctrl*, or *KP-H3*^*K36M*^*-Setd2*^*KO*^ cells. H3 and H2B are loading controls. This immunoblot was independently repeated three times with similar results. **c** Relative abundance of H3K36 and H3K27 histone modifications in *KP-H3*^*WT*^*-Ctrl* (*n* = 3 cell lines), *KP-H3*^*WT*^*-Setd2*^*KO*^ (*n* = 1 cell line), *KP-H3*^*K36M*^*-Ctrl* (*n* = 3 cell lines), and *KP-H3*^*K36M*^*-Setd2*^*KO*^ (*n* = 2 cell lines) cells by LC-MS/MS. Data are visualized in a row-normalized heat map. **d** Metagene plot of ChIP-seq data for H3K27me3, H3K36me2, and H3K36me3 at LINE elements. Plots are centered on these elements across a ± 3 kb window. **e** Metagene plot of ChIP-seq data for H3K27me3, H3K36me2, and H3K36me3 at SINE elements. Plots are centered on these elements across a ± 3 kb window. **f** Metagene plot of ChIP-seq data for H3K27me3, H3K36me2, and H3K36me3 at LTR retrotransposons. Plots are centered on these elements across a ± 3 kb window. **g** Quantitative real-time PCR for LINE and SINE element expression. Element expression is normalized to m5srRNA expression and is relative to expression of these elements in *KP-H3*^*WT*^*-Ctrl* cell lines. *n* = 2 cell lines per group.

To test whether *H3*^*K36M*^ expression induces a tumor-cell-intrinsic vulnerability to immune clearance in an orthogonal model, we engineered the *Kras*^G12D/+^;*Trp53*^-/-^ mouse lung adenocarcinoma cell line LG1233^35^ to express either *H3*^*WT*^ or *H3*^*K36M*^ together with an sgRNA targeting *Setd2* or a non-targeting control. This generated four experimental groups: *KP-H3*^*WT*^*-Ctrl*, *KP-H3*^*WT*^*-Setd2*^*KO*^, *KP-H3*^*K36M*^*-Ctrl*, and *KP-H3*^*K36M*^*-Setd2*^*KO*^ (Fig. 3a). All four cell lines had similar in vitro growth rates (Supplementary Fig. 5a). However, after orthotopic transplantation into wild-type syngeneic mice, *KP-H3*^*K36M*^*-Ctrl* cells generated only small, diffuse tumors that were highly infiltrated with immune cells (Supplementary Fig. 5b–e). In contrast, *KP-H3*^*K36M*^*-Setd2*^*KO*^ cells robustly engrafted and expanded in the lungs of recipient mice, mirroring the *Setd2*-dependent restoration of tumor growth observed in the autochthonous model. Thus, *H3*^*K36M*^ promotes immune-mediated tumor suppression through a *Setd2*-dependent mechanism in both autochthonous and transplant settings.

To determine whether adaptive or innate immune cells mediate clearance of *H3*^*K36M*^-expressing tumor cells, we transplanted *KP-H3*^*WT*^*-Ctrl*, *KP-H3*^*WT*^*-Setd2*^*KO*^, *KP-H3*^*K36M*^*-Ctrl*, and *KP-H3*^*K36M*^*-Setd2*^*KO*^ cells into immunodeficient mouse strains. In triple-immunodeficient NOD-*Prkdce*^*m26Cd52*^*;Il2rg*^*em26Cd22*^ (NCG) mice, which lack T cells, B cells, and NK cells, all four cell lines, including *KP-H3*^*K36M*^*-Ctrl* cells, formed lung tumors with similar efficiency (Supplementary Fig. 6a, b). By contrast, in athymic nude mice, which lack mature T cells but retain innate immune populations, *KP-H3*^*K36M*^*-Ctrl* cells were largely unable to form tumors. The few tumors that did arise were small and infiltrated with CD49b-positive innate immune cells, consistent with NK-cell infiltration in this setting (Supplementary Fig. 6c–e). Concordantly, in the autochthonous model, the NK-proficient, T- and B-cell-deficient *Rag1*^*KO*^ background did not restore growth of *KH3*^*K36M*^ tumors (Supplementary Fig. 6f–h). Together, these data indicate that NK cells are sufficient to mediate clearance of *H3*^*K36M*^-expressing tumor cells in the absence of T cells.

### H3^K36M^ derepresses endogenous retroviral elements

To gain insight into cancer cell autonomous programs that are controlled by H3^K36M^ and SETD2, we performed RNA-seq and ATAC-seq on *KP-H3*^*WT*^*-Ctrl*, *KP-H3*^*WT*^*-Setd2*^*KO*^, *KP-H3*^*K36M*^*-Ctrl*, and *KP-H3*^*K36M*^*-Setd2*^*KO*^ cells. *Setd2* inactivation caused few gene expression changes in *H3*^*WT*^-expressing cells. In contrast, *H3*^*K36M*^ expression induced inflammatory gene expression programs, including STAT signaling and complement pathway activation (Supplementary Fig. 7a, b). Importantly, these *H3*^*K36M*^-induced programs were *Setd2*-dependent, as they were significantly reduced in *KP-H3*^*K36M*^*-Setd2*^*KO*^ cells compared to *KP-H3*^*K36M*^*-Ctrl* cells (Supplementary Fig. 7b). ATAC-seq revealed comparatively few changes in chromatin accessibility across groups, with samples clustering primarily by *H3*^*WT*^ or *H3*^*K36M*^ status with S*etd2* inactivation having little to no effect (Supplementary Fig. 7c). Thus, the inflammatory transcriptional response induced by *H3*^*K36M*^ expression is not accompanied by broad changes in chromatin accessibility, suggesting that more focused chromatin changes may underlie this phenotype.

To define how H3^K36M^ and SETD2 alter chromatin methylation, we first measured global lysine methylation at histone tails via immunoblot and mass spectrometry on isolated histones from *KP-H3*^*WT*^*-Ctrl*, *KP-H3*^*WT*^*-Setd2*^*KO*^, *KP-H3*^*K36M*^*-Ctrl*, and *KP-H3*^*K36M*^*-Setd2*^*KO*^ cells. As expected, *KP-H3*^*WT*^*-Setd2*^*KO*^ cells had reduced H3K36me3 relative to *KP-H3*^*WT*^*-Ctrl* cells, whereas *H3*^*K36M*^-expressing cells had significantly reduced H3K36me3 and H3K36me2 (Fig. 3b, c and Supplementary Fig. 5f). In contrast, global H3K27me3 was significantly enriched in *KP-H3*^*K36M*^*-Setd2*^*KO*^ cells. To examine positional changes in histone methylation across the genome, we then performed ChIP-seq for H3K36me2, H3K36me3, and H3K27me3. Across gene bodies, *H3*^*K36M*^ expression depleted H3K36me3, while *Setd2* inactivation had a more modest effect (Supplementary Fig. 7d). *H3*^*K36M*^ expression also altered H3K36me2 distribution across genes, whereas H3K27me3 changes across gene bodies were comparatively modest.

These gene-body patterns contrasted with more pronounced changes at repetitive retroelements, including LINEs, SINEs, and LTR retrotransposons. Across LINEs, SINEs, and LTR retrotransposons, *H3*^*K36M*^ expression reduced both H3K36me2 and H3K36me3 (Fig. 3d–f). In *KP-H3*^*K36M*^*-Ctrl* cells, H3K27me3 was also reduced across these retroelements. However, in *KP-H3*^*K36M*^*-Setd2*^*KO*^ cells, H3K27me3 was restored at LINEs, SINEs, and LTR retrotransposons, consistent with repressive chromatin being re-established at these sequences when *Setd2* is inactivated (Fig. 3d–f). To determine whether loss of H3K27me3 at retroelements was associated with their transcriptional derepression, we measured LINE and SINE transcripts by RT-qPCR. LINE and SINE expression was highest in *KP-H3*^*K36M*^*-Ctrl* cells and was significantly reduced in *KP-H3*^*K36M*^*-Setd2*^*KO*^ cells (Fig. 3g). Together, these results indicate that *H3*^*K36M*^ expression promotes *Setd2*-dependent derepression of repetitive retroelements by remodeling the balance between H3K36 and H3K27 methylation at these sequences.

### H3^K36M^ promotes *Setd2*-dependent dsRNA synthesis

**Fig. 4:**
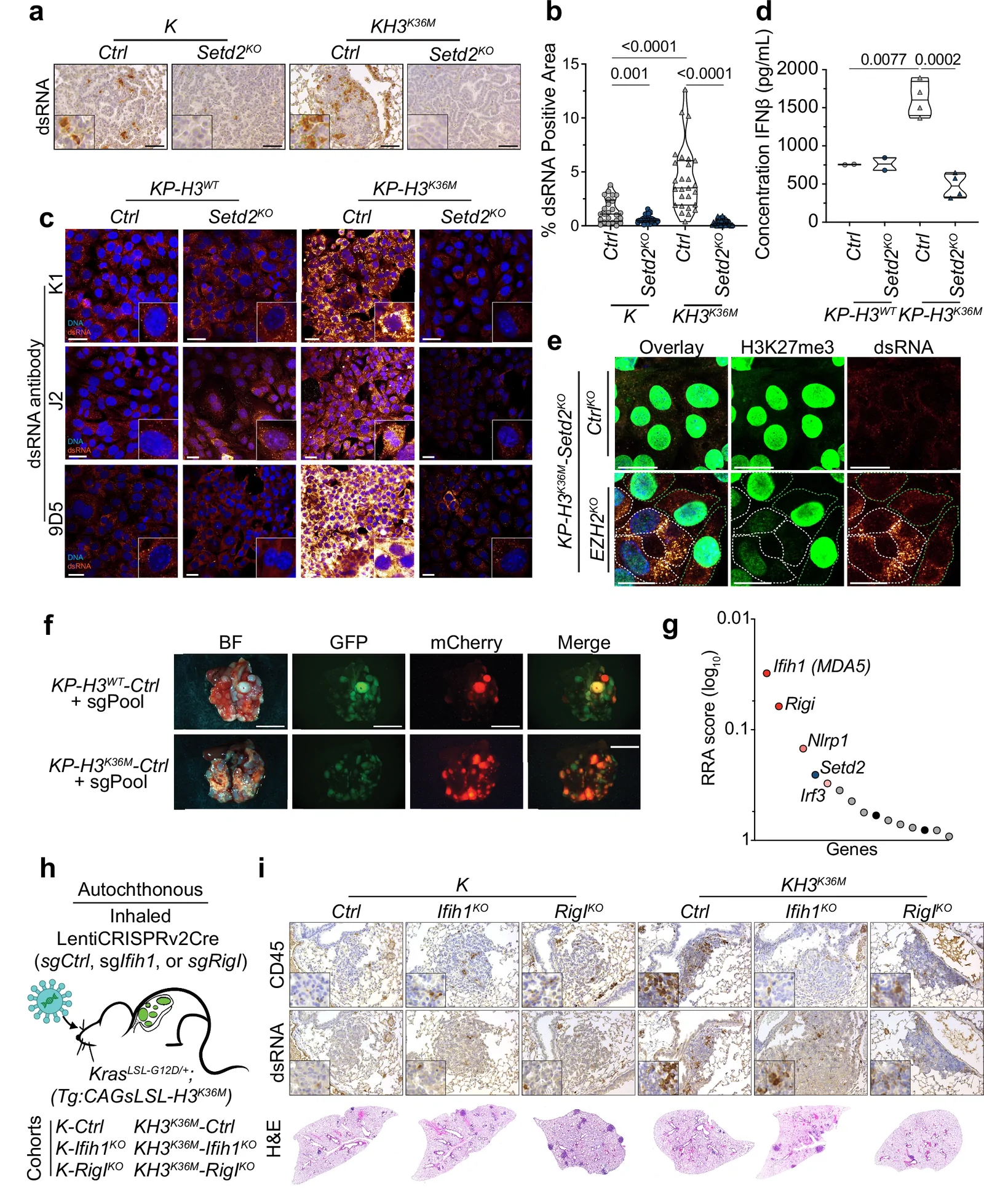
H3^K36M^ promotes *Setd2*-dependent dsRNA synthesis. **a** IHC for dsRNA in *K-Ctrl*, *K-Setd2*^*KO*^, *KH3*^*K36M*^*-Ctrl*, and *KH3*^*K36M*^*-Setd2*^*KO*^ mice. Scale bars are 119 μm; insets are magnified x3. b Quantification of dsRNA IHC in *K-Ctrl* (*n* = 25 tumors), *K-Setd2*^*KO*^(*n* = 27 tumors), *KH3*^*K36M*^*-Ctrl* (*n* = 28 tumors), and *KH3*^*K36M*^*-Setd2*^*KO*^ (*n* = 29 tumors) tumors. Significance was determined by two-sided unpaired Student’s *t*test. Violin plots show the data distribution, with the center line indicating the median and the upper and lower lines indicating the interquartile range. **c** Immunocytochemistry for dsRNA in *KP-H3*^*WT*^*-Ctrl*, *KP-H3*^*WT*^*-Setd2*^*KO*^, *KP-H3*^*K36M*^*-Ctrl*, and *KP-H3*^*K36M*^*-Setd2*^*KO*^ cells. Staining was performed in parallel using 3 dsRNA antibodies: K1, J2, and 9D5. Scale bars are 25 μm; insets are magnified x3. **d** IFN-β ELISA analysis of cell culture supernatant collected from *KP-H3*^*WT*^*-Ctrl*, *KP-H3*^*WT*^*-Setd2*^*KO*^, *KP-H3*^*K36M*^*-Ctrl*, and *KP-H3*^*K36M*^*-Setd2*^*KO*^ cells (*n* = 2 cell lines per group). Significance was determined by two-sided unpaired Student’s *t*test. Violin plots show the data distribution, with the center line indicating the median and the upper and lower lines indicating the interquartile range. **e** Immunocytochemistry for H3K36me2 (green) and K1 dsRNA (orange) in *KP-H3*^*K36M*^*-Setd2*^*KO*^*-Ctrl* and *KP-H3*^*K36M*^*-Setd2*^*KO*^*-EZH2*^*KO*^ cells. Dashed green lines represent cells with intact EZH2/H3K27me3 while dashed white lines represent cells with *EZH2*^*KO*^. Scale bars are 25 μm. No independent repetitions of this staining were conducted. **f** Brightfield (BF) and fluorescent micrographs of lungs from athymic nude mice injected with *KP-H3*^*WT*^*-Ctrl* or *KP-H3*^*K36M*^*-Ctrl* cells infected with lentiCRISPRv2mCherry + sgRNA library. GFP marks all tumor cells while mCherry marks infected cells. Scale bars are 4.4 mm. No independent repetitions of this experiment were conducted. **g** Top-ranked genes enriched in CRISPR screen determined by comparing *KP-H3*^*K36M*^*-Ctrl* cells with *KP-H3*^*WT*^*-Ctrl* cells (*n* = 10 mice per group). Enriched genes are marked in red. Positive control *Setd2* is marked in blue. Non-targeting controls are marked in black. **h** Experimental scheme of autochthonous tumor initiation. **i** IHC for CD45 and dsRNA from *K-Ctrl*, *K-Ifih1*^*KO*^, *K-*RigIKO, *K-H3*^*K36M*^*-Ctrl*, *K-H3*^*K36M*^*-Ifih1*^*KO*^, and *K-H3*^*K36M*^*-RigI*^*KO*^ mice. Scale bars are 119 μm; insets are magnified x3. Below are representative H&E images of tumor-bearing lobes from *K-Ctrl*, *K-Ifih1*^*KO*^, *K-*RigI^KO^, *K-H3*^*K36M*^*-Ctrl*, *K-H3*^*K36M*^*-Ifih1*^*KO*^, and *K-H3*^*K36M*^*-RigI*^*KO*^ mice. No independent repetitions of this experiment were conducted.

Altered chromatin regulation at retroelements and other repetitive sequences can trigger immune surveillance by promoting aberrant RNA production or processing, leading to accumulation of long double-stranded RNA species, or dsRNA^36^. We therefore hypothesized that *H3*^*K36M*^-induced retroelement derepression could promote dsRNA accumulation. Indeed, autochthonous *KH3*^*K36M*^*-Ctrl* lung tumors had significantly elevated dsRNA levels compared to *K-Ctrl* tumors (Fig. 4a, b). This increase was *Setd2*-dependent, as dsRNA detection was lost in *KH3*^*K36M*^*-Setd2*^*KO*^ tumors. Similarly, *KP-H3*^*K36M*^*-Ctrl* cells contained abundant cytoplasmic dsRNA puncta, whereas dsRNA detection was minimal in *KP-H3*^*K36M*^*-Setd2*^*KO*^ cells (Fig. 4c). We confirmed dsRNA detection using three independent dsRNA-specific antibodies, and confirmed signal specificity by RNase III pretreatment and synthetic dsRNA transfection (Fig. 4c and Supplementary Fig. 8a). Consistent with activation of dsRNA-associated antiviral signaling, *KP-H3*^*K36M*^*-Ctrl* cells produced high levels of IFN-β, a key interferon induced by dsRNA sensing, whereas *KP-H3*^*K36M*^*-Setd2*^*KO*^ cells produced little IFN-β (Fig. 4d). Thus, *H3*^*K36M*^ expression promotes cytoplasmic dsRNA accumulation and inflammatory signaling in a *Setd2*-dependent manner.

The ability of H3^K36M^ to induce dsRNA only in the presence of intact SETD2 was unexpected because both *H3*^*K36M*^ expression and *Setd2* inactivation reduce H3K36me3. To determine whether SETD2 catalytic activity is required for dsRNA accumulation in this context, we expressed either wild-type human truncated SETD2 (htSETD2^WT^) or a catalytically inactive human truncated SETD2 mutant containing R1625G and C1631A substitutions in the SET domain (htSETD2^cat mutant^) in *KP-H3*^*K36M*^*-Setd2*^*KO*^ cells. htSETD2^WT^ restored H3K36me3 and dsRNA expression, whereas htSETD2^cat mutant^ was unable to do so (Supplementary Fig. 9a–c). These data indicate that SETD2 catalytic activity is required for H3^K36M^-associated dsRNA accumulation, although the critical SETD2-dependent methylation event remains to be determined.

Because H3^K36M^ lowers H3K27me3 at retroelements and H3K27me3 is restored when *Setd2* is inactivated, we hypothesized that EZH2 contributes to retroelement silencing in *Setd2*-deficient, *H3*^*K36M*^-expressing cells. To test this, we inactivated EZH2 in *KP-H3*^*K36M*^*-Setd2*^*KO*^ cells. Cells with effective EZH2 inactivation, marked by reduced H3K27me3, showed robust restoration of cytoplasmic dsRNA puncta compared to cells with intact H3K27me3 (Fig. 4e). These findings identify EZH2 as a critical effector of retroelement silencing when SETD2 is absent and show that EZH2 loss can reinstate H3^K36M^-driven dsRNA accumulation.

To determine whether dsRNA recognition is required for H3^K36M^-induced tumor-cell killing, we performed a focused in vivo CRISPR enrichment screen targeting nucleic-acid sensors and downstream signaling effectors. We reasoned that sgRNAs disrupting the relevant sensing pathway would allow *KP-H3*^*K36M*^*-Ctrl* cells to evade immune clearance and form tumors after orthotopic transplantation into athymic nude mice, a setting in which these cells are otherwise rejected. The sgRNA library targeted genes encoding STING, IFI16, DDX41, cGAS, MDA5, RIG-I, MAVS, IKKε, IRF3, and TBK1, and included non-targeting controls and *Setd2*-targeting positive-control sgRNAs. These sgRNA-expressing constructs also express mCherry to track transduced cells (Supplementary Fig. 8b). Pooling all transduction vectors together, we transduced *KP-H3*^*WT*^*-Ctrl* or *KP-H3*^*K36M*^*-Ctrl* cells at a transduction multiplicity of ~0.4. Each transduced cell population was orthotopically transplanted into the lungs of athymic nude mice and allowed to engraft and expand for 3 weeks. Whereas *KP-H3*^*WT*^*-Ctrl* cells generated both mCherry-positive and mCherry-negative tumors, *KP-H3*^*K36M*^*-Ctrl* tumors were almost exclusively mCherry-positive, indicating selection for sgRNA-transduced cells that escaped immune clearance (Fig. 4f). To determine the identity of the relevant genes whose inactivation drove selection, we extracted DNA from the tumor-bearing lungs, PCR-amplified the sgRNA containing sequences, and subjected the resulting amplicons to high-throughput sequencing. Compared to the relative sgRNA-specific read counts in lungs transplanted with *KP-H3*^*WT*^*-Ctrl* cells, the most enriched sgRNAs in the *KP-H3*^*K36M*^*-Ctrl* tumors targeted *Ifih1* (encoding MDA5) and *Rigi* (encoding RIG-I). MDA5 and RIG-I together make up the core components of the major dsRNA-sensing pathway traditionally associated with antiviral mechanisms (Fig. 4g). As expected, Setd2-targeting sgRNAs were also highly enriched, and sgRNAs targeting Nlrp1 and Irf3 were also enriched to a lesser extent. These findings indicate that the ability of the cell to detect dsRNA species is critical for tumor-cell-killing immune stimulation induced by H3^K36M^ and again demonstrate the critical requirement for SETD2 in the process.

To validate the requirement for MDA5 and RIG-I in an autochthonous setting, we inactivated *Ifih1* or *Rigi* in *K* and *KH3*^*K36M*^ tumors (Fig. 4h). Loss of either sensor significantly increased the growth of *H3*^*K36M*^-expressing tumors (Fig. 4i and Supplementary Fig. 8c, d). Importantly, *KH3*^*K36M*^*-Ifih1*^*KO*^ and *KH3*^*K36M*^*-RigI*^*KO*^ tumors contained few infiltrating immune cells but retained abundant dsRNA-positive tumor cells (Fig. 4i and Supplementary Fig. 8e, f). Thus, MDA5 and RIG-I act downstream of H3^K36M^-induced dsRNA accumulation and are required for immune recognition and clearance of *H3*^*K36M*^-expressing tumor cells.

### NSD2 inactivation phenocopies H3^K36M^ expression

**Fig. 5:**
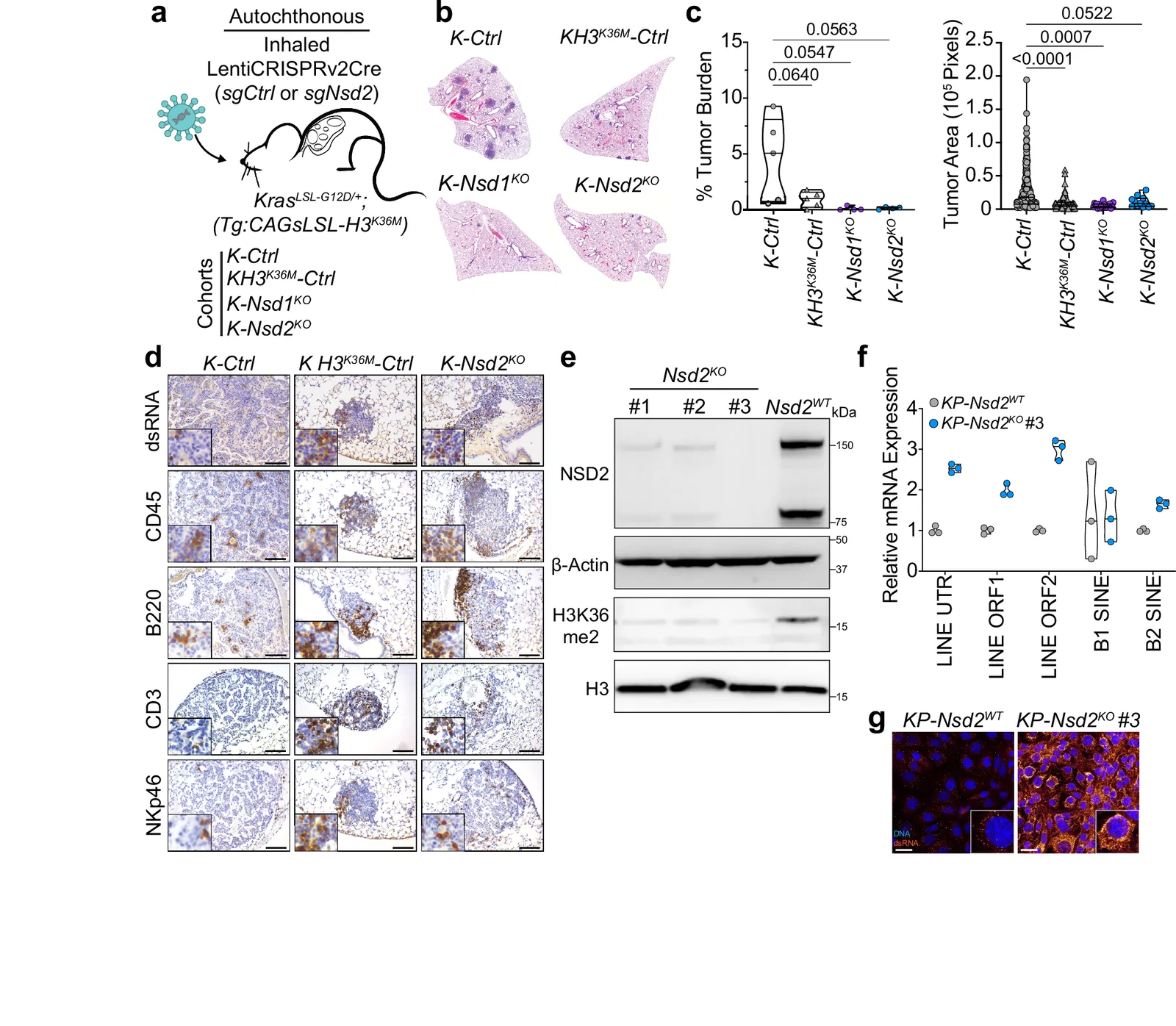
Inactivation of H3K36 di-methyltransferase *Nsd2* phenocopies H3^K36M^ expression. **a** Experimental scheme of autochthonous tumor initiation. **b** Representative images of tumor-bearing lobes from *K-Ctrl*, *KH3*^*K36M*^*-Ctrl*, *K-Nsd1*^*KO*^ and *K-Nsd2*^*KO*^ mice. **c** Quantification of tumor burden and area in *K-Ctrl* (*n* = 5 mice), *KH3*^*K36M*^*-Ctrl* (*n* = 5 mice), *K-Nsd1*^*KO*^ (*n* = 4 mice) and *K-Nsd2*^*KO*^ (*n* = 4 mice) mice. Significance was determined by two-sided unpaired Student’s *t*test. Violin plots show the data distribution, with the center line indicating the median and the upper and lower lines indicating the interquartile range. **d** IHC for dsRNA and immune cell markers CD45, B220, CD3, and NKp46 in *K-Ctrl*, *KH3*^*K36M*^*-Ctrl*, and *K-Nsd2*^*KO*^ tumors. Scale bars are 119 μm; insets are magnified x3. No independent repetitions of this staining were conducted. **e** Immunoblot analysis of NSD2 and B-Actin in whole cell lysates and analysis of H3K36me2, H3K36me3, and H3 in histones prepared from *KP-Nsd2*^*WT*^ and *KP-Nsd2*^*KO*^ cells. This immunoblot was independently repeated twice with similar results. **f** Quantitative real-time PCR for LINE and SINE element expression. Element expression is normalized to m5srRNA expression and is relative to expression of these elements in *KP-Nsd2*^*WT*^ cell lines. *n* = 3 replicates per group. **g** Immunocytochemistry for K1 dsRNA in *KP-Nsd2*^*WT*^ and *KP-Nsd2*^*KO*^ cells. Scale bars are 25 μm; insets are magnified x3.

The ability of H3^K36M^ to alter both H3K36me2 and H3K36me3 suggested that H3K36 methyltransferases other than SETD2 might contribute to H3^K36M^-induced dsRNA accumulation and immune surveillance. In particular, the strong reduction of H3K36me2 at retroelements in *H3*^*K36M*^-expressing cells suggested that inhibition of H3K36 di-methyltransferases, including NSD1 or NSD2, could contribute to retroelement derepression and subsequent immune activation (Fig. 3d–f). To determine whether *Nsd1* or *Nsd2* inactivation could replicate the effects of *H3*^*K36M*^ expression in autochthonous KRAS^G12D^-driven lung cancer, we used the same CRISPR-based somatic genome-editing approach described above (Fig. 5a). Compared to *K-Ctrl* tumors, *K-Nsd1*^KO^ and *K-Nsd2*^KO^ tumors were significantly fewer in number and smaller in size (Fig. 5b, c and Supplementary Fig. 10a). *K-Nsd1*^*KO*^ tumors were detected in only one mouse across all replicates, suggesting that NSD1 loss is poorly tolerated during KRAS-driven lung tumor development or that NSD1 has a distinct requirement in this setting. In contrast, *K-Nsd2*^KO^ tumors were present in most replicate mice, enabling further analysis.

*K-Nsd2*^*KO*^ tumors had markedly reduced H3K36me2, elevated dsRNA, and increased immune infiltration (Fig. 5d and Supplementary Fig. 10b–d). Consistent with these in vivo findings, *KP-Nsd2*^*KO*^ cell lines showed increased LINE and SINE expression, cytoplasmic dsRNA accumulation, and elevated IFN-β production (Fig. 5e–g and Supplementary Fig. 10e). Thus, *Nsd2* inactivation closely mirrors the effects of *H3*^*K36M*^ expression, supporting a model in which H3^K36M^ promotes retroelement derepression, dsRNA accumulation, and immune-mediated tumor suppression at least in part through inhibition of NSD2-dependent H3K36me2.

To assess whether the opposing effects of NSD2 and SETD2 on tumor immunity extend to human lung adenocarcinoma, we performed CIBERSORT analysis on RNA-seq data from human lung adenocarcinoma patient tumors (Supplementary Fig. 11a). Tumors with low *NSD2* expression had increased inferred immune-cell infiltration compared to tumors with high *NSD2* expression. Conversely, tumors with SETD2 loss-of-function mutations showed a significant shift in inferred immune-cell composition, including reduced total immune-cell infiltration, compared to SETD2 wild-type tumors. These findings parallel our mouse models, in which NSD2 and SETD2 have opposing effects on immune infiltration. To further explore the role of NSD2 in human lung adenocarcinoma, we also performed gene set enrichment analysis on human lung adenocarcinoma expression data. Consistent with the CIBERSORT results, gene sets related to T-cell and NK-cell function were downregulated in tumors with high *NSD2* expression (Supplementary Fig. 11b). Moreover, low *NSD2* expression was associated with significantly improved overall survival in lung adenocarcinoma patients (Supplementary Fig. 11c).

To test whether NSD2 inhibition can induce dsRNA accumulation in human lung adenocarcinoma cells, we treated a panel of human lung adenocarcinoma cell lines with the small-molecule NSD2 inhibitor KTX-1001. NSD2 inhibition induced substantial cytoplasmic dsRNA accumulation across multiple human lung cancer cell lines (Supplementary Fig. 12a). NSD2 inhibitor-treated cells also showed increased LINE and SINE expression (Supplementary Fig. 12b), consistent with pharmacologic derepression of repetitive retroelements. These data phenocopy genetic *Nsd2* inactivation and support further investigation of NSD2 inhibition as a strategy to induce dsRNA-driven viral mimicry in KRAS-driven lung adenocarcinoma.

**Fig. 6:**
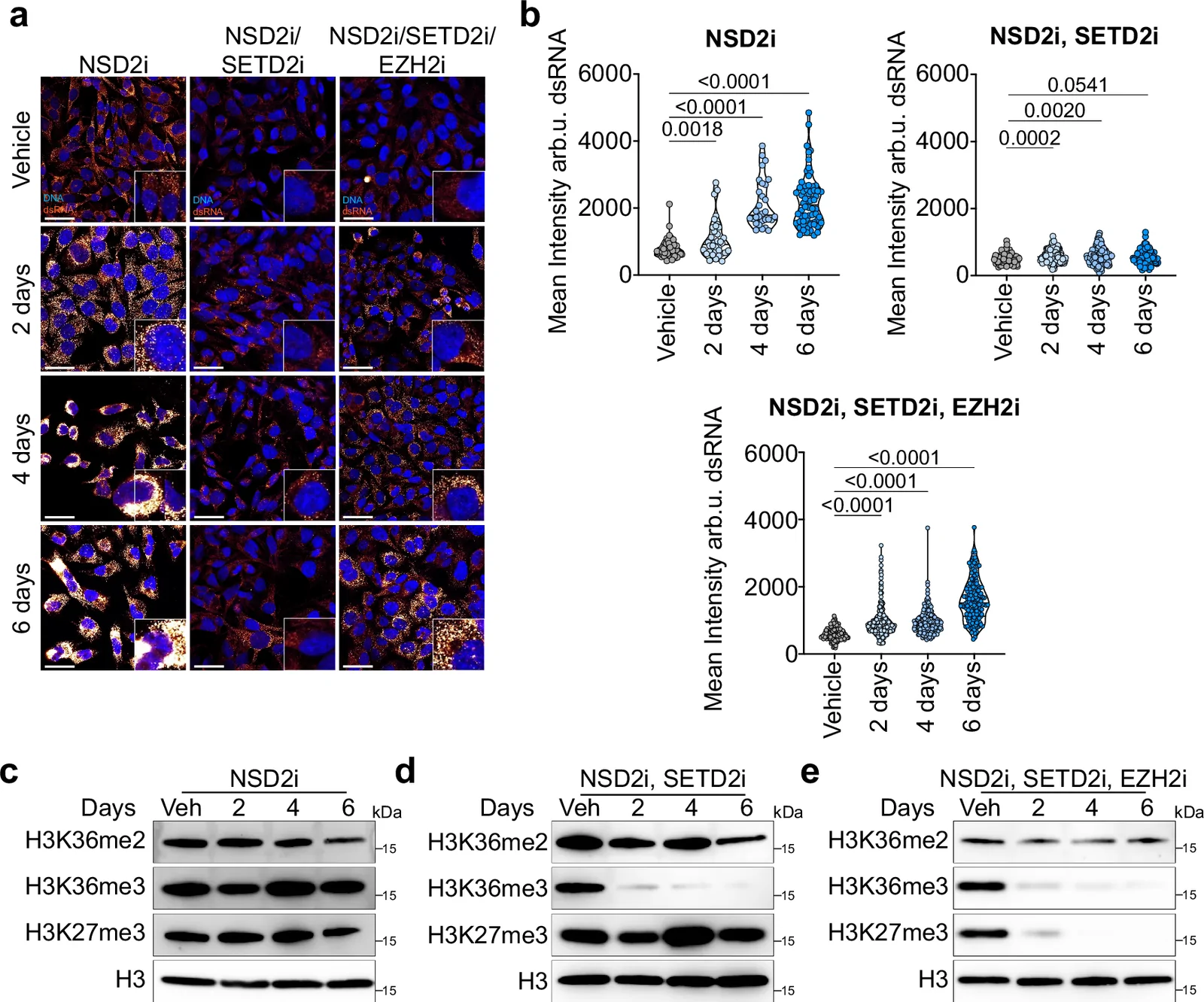
NSD2 is a therapeutic target in lung cancer. **a** Immunocytochemistry for K1 dsRNA in Calu-6 cells treated with NSD2 inhibitor, NSD2/SETD2 inhibitors, or NSD2/SETD2/EZH2 inhibitors for 2, 4, or 6 days. Veh marks Calu-6 cells treated with vehicle (DMSO) for 6 days. Scale bars are 25 μm; insets are magnified x3. **b** Quantification of dsRNA intensity in Calu-6 cells treated with NSD2 inhibitor, NSD2/SETD2 inhibitors, or NSD2/SETD2/EZH2 inhibitors for 2, 4, or 6 days. Significance was determined by two-sided unpaired Student’s *t*test. Violin plots show the data distribution, with the center line indicating the median and the upper and lower lines indicating the interquartile range. **c** Immunoblot analysis of H3K36me2, H3K36me3, H3K27me3 and H3 in histones prepared from Calu-6 cells treated with NSD2 inhibitor for 2, 4, or 6 days. Veh marks Calu-6 cells treated with vehicle (DMSO) for 6 days. H3 is a loading control. This immunoblot was independently repeated twice with similar results. **d** Immunoblot analysis of H3K36me2, H3K36me3, H3K27me3 and H3 in histones prepared from Calu-6 cells treated with NSD2 and SETD2 inhibitors for 2, 4, or 6 days. Veh marks Calu-6 cells treated with vehicle (DMSO) for 6 days. H3 is a loading control. This immunoblot was independently repeated twice with similar results. **e** Immunoblot analysis of H3K36me2, H3K36me3, H3K27me3 and H3 in histones prepared from Calu-6 cells treated with NSD2, SETD2, and EZH2 inhibitors for 2, 4, or 6 days. Veh marks Calu-6 cells treated with vehicle (DMSO) for 6 days. H3 is a loading control. This immunoblot was independently repeated three times with similar results.

While our data suggest that NSD2 inhibition is a promising avenue for therapeutic intervention in KRAS-driven lung adenocarcinoma, our results also suggest likely contraindications for such therapies. Because *H3*^*K36M*^- and *Nsd2*^*KO*^-induced dsRNA accumulation requires SETD2, we next asked whether SETD2 inhibition would blunt the response to NSD2 inhibition in human lung adenocarcinoma cells. As such, combination treatment with the NSD2 inhibitor KTX-1001 and the SETD2 inhibitor EZM0414 reduced dsRNA accumulation compared to NSD2 inhibition alone (Fig. 6a–d). However, addition of the EZH2 inhibitor tazemetostat restored dsRNA accumulation over a six-day time course (Fig. 6a, b, e). Importantly, EZH2 inhibition alone did not induce dsRNA accumulation, suggesting that EZH2 constrains dsRNA expression specifically in the context of impaired SETD2 activity (Supplementary Fig. 12c–e). Together, these data suggest that SETD2 loss may limit the ability of NSD2 inhibition to induce dsRNA, and that EZH2 inhibition can restore dsRNA accumulation in this setting, likely restoring tumor immune surveillance.

## Discussion

Although H3^K36M^ functions as an oncogenic driver in several tumor types, our data show that it has the opposite effect in KRAS-driven lung adenocarcinoma, where it suppresses tumor growth through immune-mediated tumor-cell killing. This finding is paradoxical because H3^K36M^ dominantly inhibits H3K36 methylation, yet it does not phenocopy loss of SETD2, a potent tumor suppressor and the principal H3K36 trimethyltransferase in mammalian cells [. Instead, our data indicate that H3^K36M^ diverges from SETD2 loss because it also inhibits NSD2-dependent H3K36me2, triggering retroelement derepression, dsRNA accumulation, and immune surveillance. Inactivation of the H3K36 di-methyltransferase *Nsd2* phenocopies *H3*^*K36M*^ expression by promoting retroelement derepression, dsRNA accumulation, and an antiviral-like immune response that limits tumor growth in vivo (Supplementary Fig. 13). These findings suggest that the tumor-suppressive activity of H3^K36M^ in lung adenocarcinoma is driven largely by loss of NSD2-dependent H3K36me2 rather than by simple inhibition of SETD2-dependent H3K36me3. This immune-mediated tumor suppression may be particularly effective in the lung, a tissue with robust basal immune surveillance due to continuous environmental exposure. In less inflammatory mesenchymal tissues, where *H3*^*K36M*^ is oncogenic, the chromatin- and differentiation-altering effects of *H3*^*K36M*^ may dominate over its ability to induce dsRNA-driven immune recognition.

A second unexpected finding is that *Setd2* inactivation reverses the anti-tumor effects of H3^K36M^. Because H3^K36M^ reduces H3K36me3, one might expect *H3*^*K36M*^ expression and *Setd2* loss to produce similar effects. Instead, *Setd2* loss prevents *H3*^*K36M*^-induced retroelement derepression, dsRNA accumulation, and immune activation, thereby restoring tumor growth. Our rescue experiments show that SETD2 catalytic activity is required for dsRNA accumulation in *H3*^*K36M*^-expressing, *Setd2*-deficient cells. The relevant SETD2-dependent substrate remains to be determined. One possibility is that residual SETD2 activity maintains a chromatin state that prevents EZH2-mediated re-silencing of retroelements. Another non-mutually exclusive possibility is that non-histone SETD2 substrates contribute to antiviral signaling or EZH2 regulation. For example, SETD2 has been reported to methylate STAT1, a central mediator of interferon responses, and EZH2, which catalyzes H3K27me3^18,19^. Future studies will be needed to determine whether these or other SETD2 substrates link *H3*^*K36M*^ expression to dsRNA accumulation and immune surveillance.

Our data identify EZH2 as a critical effector of retroelement silencing in *Setd2*-deficient contexts. In *H3*^*K36M*^-expressing cells, H3K27me3 is reduced at retroelements, coincident with increased retroelement expression and dsRNA accumulation. When *Setd2* is inactivated, H3K27me3 is restored at these elements and dsRNA accumulation is lost. Genetic *EZH2* inactivation restores dsRNA in *H3*^*K36M*^-expressing, *Setd2*-deficient cells, and pharmacologic EZH2 inhibition restores dsRNA accumulation in human lung adenocarcinoma cells treated with combined NSD2 and SETD2 inhibitors. Thus, SETD2 loss may limit the immunostimulatory effects of NSD2 inhibition by enabling EZH2-dependent retroelement repression, while EZH2 inhibition can relieve this repression.

These findings have important therapeutic implications. NSD2 inhibition induces LINE/SINE expression and dsRNA accumulation in human lung adenocarcinoma cells, suggesting that NSD2 targeting may activate viral mimicry programs in lung cancer. However, the SETD2-dependence of this response suggests that SETD2 status may influence sensitivity to NSD2 inhibition. In *Setd2*-deficient settings, combined inhibition of NSD2 and EZH2 may be required to restore dsRNA accumulation and promote immune recognition. Together, our findings define an NSD2–SETD2–EZH2 axis that governs retroelement repression, dsRNA-driven viral mimicry, and tumor immune surveillance, revealing a chromatin-regulated vulnerability that can be explored therapeutically in KRAS-driven lung adenocarcinoma.

## Methods

### Ethics statement

The research in this study was conducted ethically and complies with all relevant guidelines and regulations. Animal studies were performed under strict compliance with the Institutional Animal Care and Use Committee (IACUC) at the University of Pennsylvania (#804774). Our IACUC protocol states mice may harbor tumors between 0-2 mm and this limit was not exceeded.

### Animal studies and treatment

Mice were housed in a vivarium with regulated light-dark cycles (12 h), ambient temperatures (20–24^o^ C), and relative humidity (45–65%). For the autochthonous lung adenocarcinoma mouse model, *Kras*^*LSL-G12D*^ mice (Jax stock number 008179), *Rosa26*^*LSL-YFP/LSL-YFP*^, and *Tg::CAG-LSL-H3*^*K36M*^ transgenic mice have been previously described^37–39^. Mice were maintained on a C57BL/6J background. Mice were transduced with 6 × 10^4^ plaque-forming units (PFUs) per mouse of lentiCRISPRv2Cre by endotracheal intubation^40^. LentiCRISPRv2Cre expressing sgRNAs targeting *Setd2*, *Nsd2*, and β*-Galactosidase (βgal*, labeled as *sgCtrl*) were used. The sgRNA sequences are listed in Supplementary Dataset 2. *sgSetd2* and *sgCtrl* have been described previously^5,41,42^. *sgNsd2* was designed as described below in “CRISPR design and production”. Lentivirus production and titration were performed as described previously using HEK293B cells^5^. For the orthotopic lung adenocarcinoma mouse model, *Rosa26-LSL-Cas9* mice (called C57BL/6 J mice, Jax stock number 026175), OD-Prkdc^em26Cd52^; Il2rg^em26Cd22^ mice (NCG, Taconic strain 572), and CrTac: NCr-*Foxn1nu* mice (athymic nude, Taconic strain NCRNU) have been previously described. 50,000 cells were injected into the tail veins of these mice at ~4-5 weeks of age. For C57BL/6 J, NCG, and athymic nude mice, tumors formed for 5, 2, or 3 weeks, respectively. Only male NCG and athymic nude mice were used. For all other mouse experiments, roughly equal proportions of male and female mice were used. The animal protocol was approved by the University Laboratory Animal Resources (ULAR) at the University of Pennsylvania and the IACUC. We did not exceed IACUC-mandated maximum tumor volume of 2 mm. Fluorescent images of GFP- or mCherry-positive tumors in mouse lungs were captured using a Leica M80 compact stereomicroscope.

### Immunohistochemistry and Immunofluorescence

Lung and tumor tissue were dissected into 10% neutral-buffered formalin overnight at room temperature before paraffin embedding. Paraffin-embedded and H&E histological sections were produced by the Penn Molecular Pathology and Imaging Core. Immunostaining for H3^K36M^ (1:2500, Cayman Chemical Company 31547), Ki67 (1:800, Novus Biologicals NB110-89717), pH3 (1:500, Cell Signaling Technology cs9701), Nkx2.1 (1:250, abcam ab76013), and CD3 (1:700, abcam ab5690) was performed using the Leica BOND RXm Automatic Slide Stainer. Immunostaining for SPC(1:2000, Millipore AB3786), CD45 (1:100, BioLegend 103102), B220 (1:50, BD Biosciences 551459), NKp46 (1:100, BioLegend 137602), F4/80 (1:500, Bio-Rad MCA497GA), Ly6G (1:500, Biolegend 127601), and CD49b (1:500, Thermo Fisher 14-5971-85) was performed after deparaffinization and citrate based antigen retrieval. Immunostaining for H3K36me3 (1:2000, abcam ab9050) and H3K36me2 (1:1000, abcam ab9049) was performed after proteinase K antigen retrieval. Formalin-fixed, paraffin-embedded sections were incubated with avidin and biotin blocking steps for 20 min each (Vector Laboratories, SP- 2001), a 30-minute protein block (Dako, X090930-2), primary antibody overnight at 4 °C, secondary antibody for 1 h at room temperature, and with ABC reagent for 30 min at room temp (Vector Laboratories PK-4001 for rabbit antibodies and Vector Laboratories PK-6104 for rat antibodies). For J2 dsRNA (1:500, Cell Signaling Technology 76651) immunostaining, citrate-based antigen retrieval was performed, and then a Mouse on Mouse Immunodetection kit was used (Vector Laboratories, BMK-2202) according to manufacturer’s instructions.

For TUNEL staining, tissues were deparaffinized and then permeabilized with 0.1% sodium citrate and 0.1% Triton-X in PBS for 8 min. TMR Red-conjugated TUNEL labeling mix (Millipore Sigma, 12156792910) was added to tissue sections and incubated for 1 h at 37 °C in a humidity chamber. Nuclei were stained by mounting the slides with DAPI-containing Fluoro-Gel (Electron Microscopy Sciences, 17985-50). All photomicrographs were captured on a Leica DMI6000B inverted light and fluorescence microscope.

### Histological quantification

ImageJ software was used to quantify immunostained samples. Quantification was carried out as previously described^43^ for immune markers, Ki67, and J2 dsRNA. For TUNEL quantification, positive cells were counted and divided by the total area of the tumor.

### Analysis of Xenium spatial transcriptomics data

Cell typing was performed using Scanpy. Briefly, low-quality cells (<55 unique genes) were filtered out, and counts were normalized to cell area. The k-nearest neighbors graph was computed with k = 10 and *n* = 35 PCs, after which cells were embedded with UMAP and coarsely clustered at resolution 0.5 with Leiden clustering. Clusters were annotated based on differentially expressed genes and, in some cases, spatial distributions. For instance, AT2 cells and AT2-like malignant cells initially clustered together due to similar gene expression profiles but could be distinguished in sub-clustering by spatial location (ie, in tumor nodules or lung parenchyma). Cellular neighborhood analysis was performed using Scimap. First, the nearest 30 spatial neighbors were determined for each cell, and cells were k-means clustered, using k = 30 to overcluster cells into neighborhoods. Then, neighborhoods were manually merged based on similarity in cell type abundance and distribution in space. Individual tumors were identified by computing the density of malignant cells in 50 × 50 micron bins, and bins were marked as ‘tumor’ if they contained >20% malignant cells. Tumors were further pruned if they had fewer than 10 malignant cells, resulting in a total of 159 tumors across the four genotypes. T cell activation score was computed using Scanpy’s score_genes function on the gene list GO:0050870 (Positive regulation of T cell activation), considering only genes measured in the Xenium experiment. Hotspot analysis^34^ was performed to identify spatially variable programs of gene expression. Briefly, malignant cells were subsetted and their spatial positions were input to the Hotspot algorithm as the similarity graph. Hotspot then identifies genes whose expression is highly autocorrelated on the similarity graph (in this case, in tissue space) and groups genes into modules on this basis. The module gene expression is then scored on a single-cell basis by averaging the standardized residuals from a DANB model for all genes assigned to the module.

### Cell Lines

LG1233 cells were generated from lung tumors of C57BL/6J *Kras*^G12D/+^; *Trp53*^*flox/flox*^ mice and kindly provided by Dr. Tyler Jacks (Massachusetts Institute of Technology, Cambridge, USA). All cell lines were grown in high-glucose DMEM supplemented with 10% fetal bovine serum, GlutaMAX, and gentamicin at 37 °C and 5% CO_2_. *H3.3*^*WT*^ or *H3.3*^*K36M*^ genes were cloned into MSCV puro (Addgene 68469). Retrovirus was produced, and LG1233 cells were infected using polybrene at 4 μg/ml. Infected cells were selected using 2.5 μg/ml puromycin. Next, lentivirus was produced using lentiCRISPRv2GFP (Addgene 82416) with sgRNAs targeting either *Setd2* or *βgal* (sgRNA sequences above and in Supplementary Dataset 2). *KP-H3*^*WT*^ or *KP-H3*^*K36M*^ cells were infected with these lentiviruses and then sorted for GFP positivity by FACS. Next, 1000 resultant cells were plated on a 15 cm dish and expanded for 1-2 weeks until colonies formed. Colonies were individually trypsinized and grown separately as clones. Clones were then screened for *H3*^*K36M*^ expression and *Setd2*^*KO*^ by immunoblot analysis. *KP-Nsd2*^*KO*^ cell lines were generated by transducing LG1233 cells with lentiCRISPRv2Puro (Addgene 98290) expressing an sgRNA targeting *Nsd2* or *βgal* (as a control), followed by single-cell cloning (described above), and validation of NSD2 loss by immunoblotting. *KP-H3*^*K36M*^*-Setd2*^*KO*^*-EZH2*^*KO*^ cell lines were produced using lentiCRISPRv2mCherry (Addgene 99154) with sgRNAs targeting either *EZH2* or *βgal* (as a control) and transducing *KP-H3*^*K36M*^*-Setd2*^*KO*^ cells with these constructs.

Cell number was counted using a Beckman Coulter Z2 Cell and Particle Counter. Proliferation of cell lines was determined by plating 25,000 cells, counting cells, and replating 25,000 cells every 4 days in triplicate.

### Cloning and stable expression of truncated SETD2 constructs

Truncated human SETD2 (amino acids 567–2564) and the catalytic mutant (R1625G/C1631A) were cloned into the MSCV-hygro retroviral vector by Gibson assembly. Mutations were introduced by PCR-based mutagenesis, and an HA-Flag epitope tag was fused to the construct for detection. Primer sequences used for cloning and mutagenesis are listed in Supplementary Dataset 3. The resulting htSETD2^WT^ and htSETD2^cat mutant^ constructs were sequence verified and used for retroviral transduction into *KP*-*H3*^*K36M*^*-Setd2*^*KO*^ cells. Transduced cells were selected with 800 μg/mL hygromycin. Expression of HA-Flag-tagged htSETD2 (~250 kDa) was confirmed by HA and SETD2 immunoblotting.

### Immunoblot analysis

For whole cell lysates, cells were lysed in RIPA buffer. Acid-extracted histones were prepared for histone methylation Western blots. Samples were resolved on NuPage 4–12% (for whole cell lysates) or 12% (for histones) Bis-Tris protein gels (Thermo Fisher) and transferred to polyvinylidene fluoride (PVDF) membranes. Blocking, primary, and secondary antibody incubations were performed in Tris-buffered saline (TBS) with 0.1% Tween-20. Histones were blotted for H3^K36M^ (1:1000, Cayman Chemical Company 31547), H3K36me3 (1:1000, Abcam ab9050), H3K36me2 (1:1000, Abcam ab9049), H3K27me3 (1:1000, Cell Signaling Technology 9733), H3 (1:20000, Abcam ab1791), and H2B (1:20000, Abcam ab1790). Whole cell lysates were blotted for NSD2 (1:1000, Abcam, ab75359), SETD2 (1:1000, Cell Signaling Technology 80290), HA-Tag (1:1000, Cell Signaling Technology 3724) and β-actin (1:10000, Sigma Aldrich, A2066). Protein concentration was determined using a Bradford Protein Assay Kit (Thermo Scientific A55866). Uncropped immunoblots are provided in Supplementary Fig. 14.

### Histone extraction

Histone proteins were extracted from the pellet as described by ref. ^44^ to ensure good-quality identification and quantification of single histone marks. Briefly, histones were acid-extracted with 0.4 N sulfuric acid and incubated with constant rotation for 30 min, followed by precipitation with 33% trichloroacetic acid (TCA) for 1 h at 4 C. Then, the supernatant was removed and the tubes were rinsed with acetone containing 0.1% 12 N HCl, centrifuged, and rinsed again using 100% acetone. After the final centrifugation, the supernatant was discarded, the pellet was dried at room temperature for 30 min, and the histones were resuspended in molecular-grade H_2_O. The histones were then used for either immunoblotting or mass spectrometry.

### Histone digestion and desalting

The pellet was dissolved in 50 mM ammonium bicarbonate, pH 8.0, and histones were subjected to derivatization using 5 µL of propionic anhydride and 14 µL of ammonium hydroxide (all Sigma Aldrich) to balance the pH at 8.0. The mixture was incubated for 15 min and the procedure was repeated. Histones were then digested with 1 µg of sequencing grade trypsin (Promega) diluted in 50 mM ammonium bicarbonate (1:20, enzyme:sample) overnight at room temperature. Derivatization reaction was repeated to derivatize peptide N-termini. The samples were dried in a vacuum centrifuge. Prior to mass spectrometry analysis, samples were desalted using a 96-well plate filter (Orochem) packed with 1 mg of Oasis HLB C-18 resin (Waters). Briefly, the samples were resuspended in 100 µl of 0.1% TFA and loaded onto the HLB resin, which was previously equilibrated using 100 µl of the same buffer. After washing with 100 µl of 0.1% TFA, the samples were eluted with a buffer containing 70 µl of 60% acetonitrile and 0.1% TFA and then dried in a vacuum centrifuge.

### LC-MS/MS acquisition and analysis

Samples were resuspended in 10 µl of 0.1% TFA and loaded onto a Dionex RSLC Ultimate 300 (Thermo Scientific), coupled online with an Orbitrap Fusion Lumos (Thermo Scientific). Chromatographic separation was performed with a two-column system, consisting of a C-18 trap cartridge (300 µm ID, 5 mm length) and a picofrit analytical column (75 µm ID, 25 cm length) packed in-house with reversed-phase Repro-Sil Pur C18-AQ 3 µm resin. Peptides were separated using a 30 min gradient from 1-30% buffer B (buffer A: 0.1% formic acid, buffer B: 80% acetonitrile + 0.1% formic acid) at a flow rate of 300 nl/min. The mass spectrometer was set to acquire spectra in a data-independent acquisition (DIA) mode. Briefly, the full MS scan was set to 300–1100 m/z in the orbitrap with a resolution of 120,000 (at 200 m/z) and an AGC target of 5x10e5. MS/MS was performed in the orbitrap with sequential isolation windows of 50 m/z with an AGC target of 2x10e5 and an HCD collision energy of 30.

Histone peptides raw files were imported into EpiProfile 2.0 software^45^. From the extracted ion chromatogram, the area under the curve was obtained and used to estimate the abundance of each peptide. In order to achieve the relative abundance of post-translational modifications (PTMs), the sum of all different modified forms of a histone peptide was considered as 100% and the area of the particular peptide was divided by the total area for that histone peptide in all of its modified forms. The relative ratio of two isobaric forms was estimated by averaging the ratio for each fragment ion with different mass between the two species. The resulting peptide lists generated by EpiProfile were exported to Microsoft Excel and further processed for a detailed analysis.

### RNA-sequencing preparation and analysis

RNA isolation, library preparation, and sequencing of 3 clonal cell lines per group (*KP-H3*^*WT*^*-Ctrl*, *KP-H3*^*WT*^*-Setd2*^*KO*^, *KP-H3*^*K36M*^*-Ctrl*, and *KP-H3*^*K36M*^*-Setd2*^*KO*^) were performed by Genewiz/Azenta Life Sciences. Libraries were subjected to Illumina 150-bp paired-end sequencing.

Reads from fastq files were mapped to the mouse genome (GRCm39) using Salmon with ‘libType A’ and ‘validateMapping’ options^46^. The reads mapped to coding genes were then used as an input to DESeq to perform differential analysis^47^. For volcano plot data, differentially expressed genes were defined as genes with a false-discovery rate-adjusted *P* value less than 0.05 and log_2_-normalized fold change in expression greater than ± 1. For the pathway enrichment analysis, the gene counts were used as input to the GSEA program^48^. Enrichment analysis was performed on the Mouse Hallmark and Biological Processes gene sets from MSigDb^48^. Heatmaps were generated using the normalized counts from DESeq using the pheatmap package^49^. In addition, ashr^50^, ggplot^51^, snakemake^52^, tximport^53^, and rtracklayer^54^ packages were used for performing these analyses.

### ATAC-sequencing preparation and analysis

ATAC-seq libraries were generated and sequenced by Azenta Life Sciences from 3 clonal cell lines per group (*KP*-*H3*^*WT*^*-Ctrl*, *KP*-*H3*^*WT*^*-Setd2*^*KO*^, *KP*-*H3*^*K36M*^*-Ctrl*, and *KP*-*H3*^*K36M*^*-Setd2*^*KO*^). Libraries were subjected to Illumina 150-bp paired-end sequencing.

ATAC-seq FASTQ files were trimmed using Atria (default options) to remove adapter reads and were then aligned to mm39 (GRCm39) using bowtie2 (“bowtie align” using options –local –no-mixed –discordant, v2.5.1)^55^. Peak calling was performed on the aligned reads using Genrich with the -j and -d 150 options^56^. BigWig files were generated using bamCoverage from deeptools. Using deeptools bigWigsummary and plotCorrelation, the correlation heatmaps were plotted for these samples^57^.

### Chromatin immunoprecipitation (ChIP)

15 million cells from 2 clonal cell lines per group (*KP-H3*^*WT*^*-Ctrl*, *KP-H3*^*WT*^*-Setd2*^*KO*^, *KP-H3*^*K36M*^*-Ctrl*, and *KP-H3*^*K36M*^*-Setd2*^*KO*^) were harvested and cross-linked at room temperature by resuspending cells in PBS containing 1% PFA for 5 min. Crosslinking was quenched by the addition of glycine to a final concentration of 125 mM. After washing cells twice with PBS, cells were lysed in 1 mL sonication buffer (0.25% Sarkosyl, 1 mM DTT, and protease inhibitor in RIPA buffer). Cell suspensions were sonicated using a Covaris S220 Focused Ultrasonicator until chromatin was sheared for 17 min per sample to a size range of around 200- 500 bp. Lysates were centrifuged at a maximum speed for 5 min, supernatant was collected, and lysates were flash frozen in liquid nitrogen. The next day, Protein A Dynabeads (Invitrogen, 10001D) were incubated for 6 hours with H3K36me2 (5 µg, abcam, ab9049), H3K36me3 (5 µg, abcam, ab9050), H3K27me3 (5 µg, Cell Signaling Technologies, 9733 T), or mouse IgG (1 µg, Diagenode, C15400001-15) antibody at 4 °C. Sonicated chromatin was thawed and mixed with 2% sonicated S2 chromatin as a spike-in control. Chromatin from each sample was mixed and used as a single input control. Sonicated samples were added to the pre-bound Dynabeads and rotated overnight at 4 °C. Chromatin-bound beads were washed three times with low-salt wash buffer (0.1% SDS, 1% Triton X-100, 1 mM EDTA pH 8.0, 50 mM Tris-HCl at pH 8.0, 150 mM NaCl), high-salt wash buffer (0.1% SDS, 1% Triton X-100, 1 mM EDTA pH 8.0, 50 mM Tris-HCl pH 8.0, 500 mM NaCl), and LiCl wash buffer (150 mM LiCl, 0.1% SDS, 0.5% deoxycholic acid sodium salt, 1% NP-40, 1 mM EDTA pH 8.0, 50 mM Tris pH 8.0). Beads were then washed with TE buffer containing 50 mM NaCl and spun down at 950 g. The beads were resuspended in ChIP elution buffer (1% SDS, 200 mM NaCl, 10 mM EDTA, pH 8.0, 50 mM Tris pH 8.0) and eluted for 30 min at 65 °C. The solutions were spun down for 1 min at 16000 g, and supernatants were transferred to new tubes. Input DNA was diluted to 200 µl using ChIP elution buffer. Input and experimental samples were reverse cross-linked overnight at 65 °C. All samples were incubated with RNase A for 1 hour at 37 °C, followed by Proteinase K treatment for 1 hour at 55 °C. DNA was purified using a PCR purification Kit (Qiagen, 28104) and eluted into 50 µl of molecular biology grade water. Libraries were generated by Genewiz/Azenta Life Sciences (2 × 150 bp paired-end sequencing; Illumina HiSeq).

### ChIP sequencing analysis

For ChIP-seq samples, spike-in normalization was performed as in ref. ^58^. Drosophila chromatin was added to the histone ChIP samples before the IP was performed to a concentration of 2%. The reads from these samples were first trimmed using Atria (default options, v4.0.0)^59^ and then aligned to mm39 (GRCm39) using bowtie2 (“bowtie align” using options –local –no-mixed –discordant, v2.5.1)^55^. Reads were then also aligned to the Drosophila genome dm6 (BDGP Release 6 + ISO1 MT), and the number of reads mapping to this genome was counted, and the percentage of Drosophila reads in each sample was calculated. Scaling factors for each sample were then calculated by scaling up human reads such that the percentage of Drosophila reads in each sample was the same. Using DeepTools^57^, bigWig files for the samples were then generated using the scaleFactors option while providing respective scaling factors calculated in the previous step. RepeatMasker masked repeat Sequences were downloaded from the UCSC Genome browser database^60^. Using Deeptools, matrices were computed using the ‘computeMatrix’ command for the different repeat elements and plotted using the ‘plotHeatmap’ command.

### Quantitative reverse transcription-PCR

Total RNA was extracted from cells using Qiagen RNeasy Mini Kit (Qiagen, 74106). cDNA synthesis was performed using High-Capacity cDNA Reverse Transcription Kit (Applied Biosystems, 4368814). RT-PCR was performed using PowerUP SYBR Green (Invitrogen, A25742) in triplicate, following manufacturer’s instructions, and evaluated on an Applied Biosystems ViiA 7 RT-PCR machine. RT-PCR primers are listed in Supplementary Dataset 4.

### Immunocytochemistry

25,000 *KP*-*H3*^*WT*^*-Ctrl*, *KP*-*H3*^*WT*^*-Setd2*^*KO*^, *KP*-*H3*^*K36M*^*-Ctrl*, and *KP*-*H3*^*K36M*^*-Setd2*^*KO*^ cells were plated in chamber slides (EMD Millipore PEZGS0816) and grown overnight. Cells were then fixed with 4% PFA, permeabilized with 0.1% Triton X-100 detergent in 1X PBS, blocked with 1% BSA in 1X PBS, and stained overnight with anti-dsRNA antibodies K1 (1:800, Cell Signaling Technology 28764), J2 (1:500, Cell Signaling Technology 76651), or 9D5 (1:500, Ab00458-23.0) or anti-H3K27me3 antibody (1:800, Cell Signaling Technology 9733) diluted in blocking buffer. Cells were then stained with either anti-rabbit IgG strepavidin-Alexa Fluor 594 (1:200, Thermo Fisher A32754) or anti-mouse biotinylated secondary antibody (1:200, Vector Laboratories BA-9200) for 1 h at room temperature, and then with strepavidinAlexa Fluor 594 (1:200, Thermo Fisher S32356) or antistrepavidin Alexa Fluor 647 (1:200, Thermo Fisher S32357). Nuclei were stained with DAPI. Cells were then mounted with Fluoromount-G (Southern Biotech 0100-01), and imaging was performed on a Leica Stellaris 5 confocal microscope using a 63x oil objective and fluorescence intensity was analyzed with LASX software.

For RNase III treatment, cells were treated with ShortCut RNase III (New England Biolabs M0245) after permeabilization for 2 hr at room temperature according to manufacturer’s instructions. For poly(I:C) treatment (Sigma Aldrich P1530), cells were plated as above and then transfected with 0.25 μg poly(I:C) using Lipofectamine 3000 Transfection Reagent (Thermo Fisher L3000001) following manufacturer’s instructions. For both of these treatments, the staining protocol was then followed as above.

### Inhibitor assay

Cells were treated with the NSD2 inhibitor KTX-1001 (1 μM; ProbeChem, PC-21038), the EZH2 inhibitor Tazemetostat (1.25 μM; MCE, HY-13803), or the SETD2 inhibitor EZM0414 (1 μM; MCE, HY-144858), either alone or in combination (KTX-1001 + EZM0414 or all three inhibitors together), for 2, 4, or 6 days, with media and inhibitors replenished every 2 days. Following treatment, cells on chamber slides were fixed and processed for immunofluorescence staining as described above. In parallel, histones were extracted from cells as described above and analyzed by immunoblotting to assess changes in histone methylation status.

### IFNB1 ELISA

Mouse interferon beta (IFNB1) levels were quantified using the Mouse IFNB1 ELISA Kit (Aviva Systems Biology, OKEH00314), according to manufacturer’s instructions. Briefly, pre-coated anti-IFNB1 plates were incubated with standards and experimental samples for 2 h at room temperature. 1x biotinylated IFNB1 detection antibody (1:100) was added to each well and incubated for 1 h. Samples were then incubated with 1X Avidin-HRP conjugate (1:100) for 30 min. Lastly, TMB substrate was added to develop the colorimetric signal. The reaction was stopped with the provided stop solution, and absorbance was measured at 450 nm using a Tecan Infinite 200 Pro microplate reader. Sample concentrations were determined using the standard curve generated using serial dilutions of the IFNB1 standard.

### CRISPR design and production

All sgRNAs not delineated above were designed using the Sanjana Lab CRISPR Cas9 library design tool (http://guides.sanjanalab.org). For in vivo CRISPR, sgRNA duplexes were Golden Gate cloned into BsmBI sites of the LentiCRISPRv2-Cre vector. LentiCRISPRv2GFP sg*Setd2* and sg*Ctrl* were cloned in this manner as well. For the CRISPR enrichment screen library generation, the In-Fusion cloning system (Clontech: #638909) was used. The sgRNA pool was cloned by annealing two DNA oligos for each guide and ligating into a BsmB1-digested lentiCRISPRv2mCherry vector (Addgene 99154), as described^61^. The sgRNA sequences used are detailed in Supplementary Dataset 2. For *RigI* and *Ifih1* single gene knockout experiments in Fig. 4 h-i, sgIfih1 #3 (GCACCTACGCACTTTCCCAG) and sgRigI #3 were used.

### Pooled CRISPR enrichment screen

The sgRNA library targeting dsRNA sensing pathway components was generated as described above. Lentivirus was produced and titered. *KP-H3*^*WT*^*-Ctrl* and *KP-H3*^*K36M*^*-Ctrl* cells were infected at an MOI of 0.4 to ensure a single copy of sgRNA transduction per cell. 50,000 of the resultant cells were injected into the tail veins of 10 athymic nude mice per group. After 3 weeks, the mice were euthanized, their lungs were isolated, and fluorescent images were taken of their lung tumors. Each lung tree was placed into 10 mL of digestion buffer containing 100 mM NaCl, 20 mM Tris, 10 mM EDTA, 0.5% SDS, and 100 μL of 20 mg/mL Proteinase K. The tissue was cut into small pieces and incubated at 55 °C overnight. The next day, genomic DNA was extracted from 5 mL of each digested sample by phenol-chloroform extraction.

To amplify the tumor sgRNA sequences, 20 μg of genomic DNA was PCR amplified. The genomic DNA from the original cell lines infected with the sgRNA library was also extracted and PCR amplified as a benchmark for sgRNA representation. Q5 Hot Start High-Fidelity 2x Master Mix (New England Biolabs, M0494X) was mixed with genomic DNA and a universal forward and reverse primer set (Forward: 5’ CGTGACGTAGAAAGTAATAATTTCTTGGGTAG 3’, Reverse: 5’ GAGGCCGAATTCAAAAAAGCACC 3’). The PCR products were then subjected to double size selection to remove genomic DNA and primer dimer using AmPure XP beads (Beckman Coulter, A63881). 5% of the product was run on an agarose gel to ensure proper product size.

The PCR products were then library-prepped and sequenced by Genewiz/Azenta Life Sciences using their Amplicon-EZ platform. Libraries were subjected to 2 × 250 bp Illumina sequencing. MAGeCK was used to calculate the read count of each individual sgRNA with no mismatches and compared to the sequence of the reference sgRNA. The read counts from the PCR amplified DNA isolated from the in vivo tumors were normalized to the read counts of the parental cell lines. MAGeCK was then used to identify positively enriched sgRNAs in *KP-H3*^*K36M*^*-Ctrl* + sgRNA pool injected mice relative to *KP-H3*^*WT*^*-Ctrl* + sgRNA pool injected mice using the default settings.

### Human dataset analysis

For gene expression analysis in human lung adenocarcinoma, RNA-sequencing data were obtained from the Cancer Genome Atlas lung adenocarcinoma dataset. All genes were ranked in order according to the genes most positively correlated with *Nsd2* expression to most negatively correlated, and this rank list was used to perform Gene Set Enrichment Analysis (GSEA) examining GO biological processes using the molecular signature database (MSigDB). Kaplan–Meier survival curves for lung adenocarcinoma patients with tumors expressing low (*n* = 581) or high (*n* = 580) levels of *Nsd2* were obtained from KM plotter (kmplot.com).

### CIBERSORT

To quantify infiltrating immune cell abundance, deconvolution was performed on bulk tumor RNA-seq profiles from the Cancer Genome Atlas Lung Adenocarcinoma (TCGA-LUAD) cohort using the CIBERSORTx framework against the LM22 signature matrix (22 leukocyte phenotypes). The algorithm was executed in Absolute Mode using 100 permutations and B-mode batch correction to account for cross-platform technical variation. Unlike standard relative fractions that mathematically constrain the data to sum to 1, Absolute Mode scales relative proportions by a sample-specific normalization factor proportional to the total immune transcriptomic signal. The resulting “Absolute Score” serves as a digital surrogate for total immune cell density, enabling quantitative comparisons across samples with varying tumor purity. Samples were stratified by genomic status (*SETD2* Loss-of-Function vs. Wild-Type; *NSD2* Top 10% High vs. Bottom 10% Low), and statistical differences in cumulative leukocyte infiltration were evaluated using a two-tailed Welch’s t-test. All processing and visualizations were executed in R using tidyverse and ggplot2.

### Statistics and reproducibility

All statistical analyses were performed using GraphPad Prism 10. For violin plots, two-sided unpaired Student’s *t*tests were performed. Sample sizes for individual experiments are indicated in the figures. Reproducibility of the findings was confirmed by analyzing at least *n* = 3 technical and/or biological replicates. The findings of all replicates were consistent.

### Reporting summary

Further information on research design is available in the Nature Portfolio Reporting Summary linked to this article.

## Supplementary information

Supplementary Information

Description of Additional Supplementary Files

Supplementary Dataset 1

Supplementary Dataset 2

Supplementary Dataset 3

Supplementary Dataset 4

Reporting Summary

Transparent Peer Review file

## Source data

Source Data

## Data Availability

All sequencing data generated in this study have been deposited in the NCBI Gene Expression Omnibus (GEO) database under accession codes GSE335211 (https://www.ncbi.nlm.nih.gov/geo/query/acc.cgi?acc=GSE335211) (Xenium), GSE341379 (https://www.ncbi.nlm.nih.gov/geo/query/acc.cgi?acc=GSE341379) (RNA-, ATAC-, and ChIP-sequencing), and GSE334916 (https://www.ncbi.nlm.nih.gov/geo/query/acc.cgi?acc=GSE334916) (CRISPR enrichment screen). The mass spectrometry raw files were uploaded in the online repository ProteomeXchange (PRIDE) under the project number PXD069619 (https://www.ebi.ac.uk/pride/archive/projects/PXD069619). Source data are provided with this paper. Publicly available RNA-sequencing and associated clinical data from The Cancer Genome Atlas Lung Adenocarcinoma (TCGA-LUAD) cohort were obtained from the cBioPortal for Cancer Genomics (https://www.cbioportal.org/) under the TCGA Lung Adenocarcinoma (Firehose Legacy) study. Source data are provided with this paper.
