# Supplementary material for: Antagonistic roles of histone H3 lysine methyltransferases dictate tumor immune surveillance
Source: Nat Commun. 2026 Sep 14;17:10810. doi: 10.1038/s41467-026-76657-3 (PMC13669384; doi:10.1038/s41467-026-76657-3)
Supplement: Supplementary file 1 [file 41467_2026_76657_MOESM1_ESM.pdf]

Supplementary Information

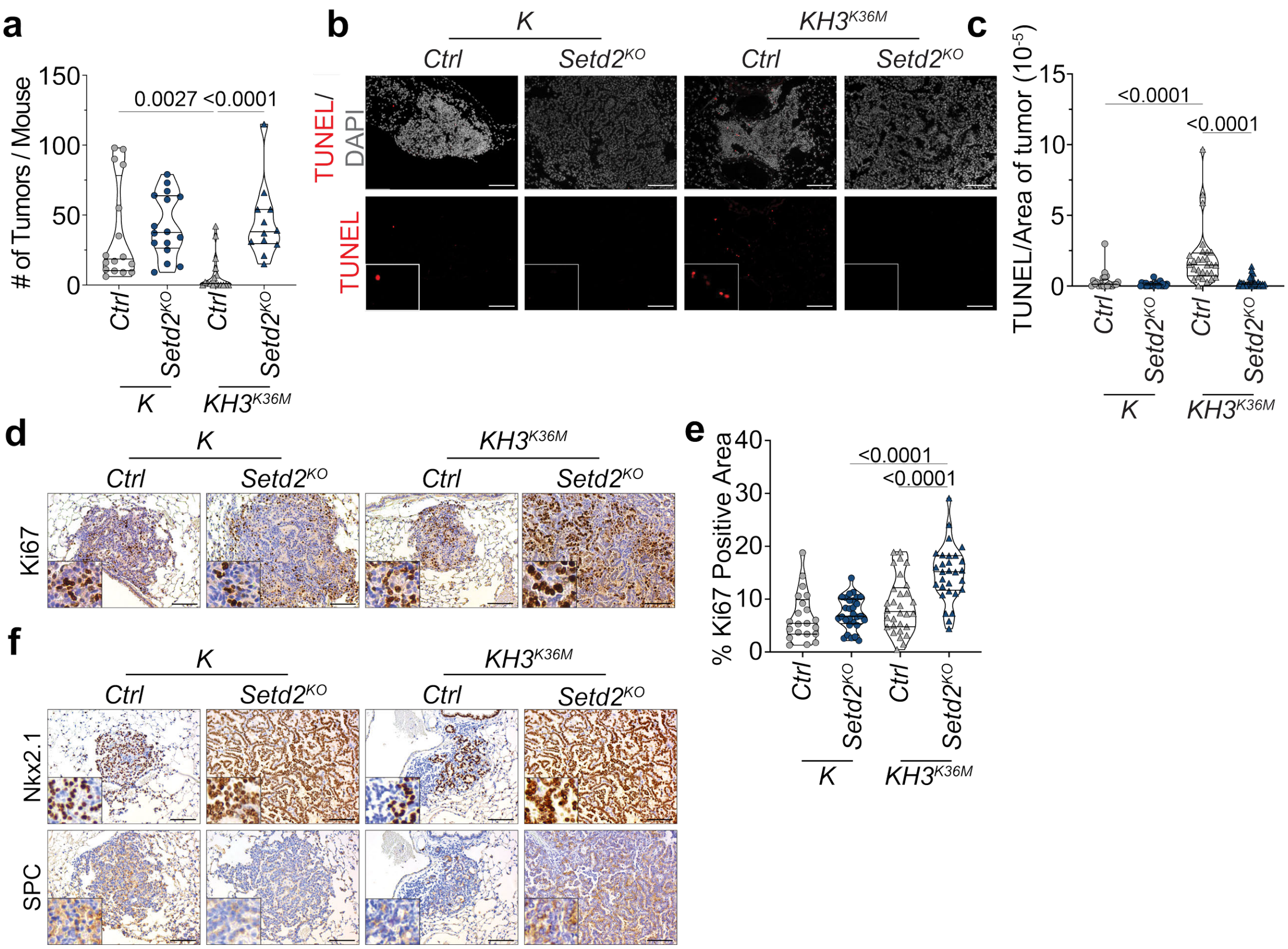

Supplementary Figure 1: H3^{K36M} promotes tumor cell death in a Setd2-dependent manner
a) Quantification of tumor number in *K-Ctrl* (n=16 mice), *K-Setd2*^{KO} (n=16 mice), *KH3*^{K36M}*-Ctrl* (n=17 mice), and *KH3*^{K36M}*-Setd2*^{KO} (n=12 mice) mice. Significance was determined by two-sided unpaired Student's t-test. Violin plots show the data distribution, with the center line indicating the median and the upper and lower lines indicating the interquartile range. **b)** TUNEL staining of tumors from *K-Ctrl*, *K-Setd2*^{KO}, *KH3*^{K36M}*-Ctrl*, and *KH3*^{K36M}*-Setd2*^{KO} mice. Scale bars are 119 μm; insets are magnified x3. **c)** Quantification of TUNEL staining in *K-Ctrl* (n=21 tumors), *K-Setd2*^{KO} (n=26 tumors), *KH3*^{K36M}*-Ctrl* (n=28 tumors), and *KH3*^{K36M}*-Setd2*^{KO} (n=31 tumors) tumors. Significance was determined by two-sided unpaired Student's t-test. Violin plots show the data distribution, with the center line indicating the median and the upper and lower lines indicating the interquartile range. **d)** IHC for Ki67 in *K-Ctrl*, *K-Setd2*^{KO}, *KH3*^{K36M}*-Ctrl*, and *KH3*^{K36M}*-Setd2*^{KO} tumors. Scale bars are 119 μm; insets are magnified x3. **e)** Quantification of Ki67 IHC staining in *K-Ctrl* (n=21 tumors), *K-Setd2*^{KO} (n=29 tumors), *KH3*^{K36M}*-Ctrl* (n=30 tumors), and *KH3*^{K36M}*-Setd2*^{KO} (n=29 tumors) mice. Significance was determined by two-sided unpaired Student's t-test. Violin plots show the data distribution, with the center line indicating the median and the upper and lower lines indicating the interquartile range. **f)** IHC for lung lineage markers Nkx2.1 (TTF1) and SPC in *K-Ctrl*, *K-Setd2*^{KO}, *KH3*^{K36M}*-Ctrl*, and *KH3*^{K36M}*-Setd2*^{KO} tumors. Scale bars are 119 μm; insets are magnified x3.

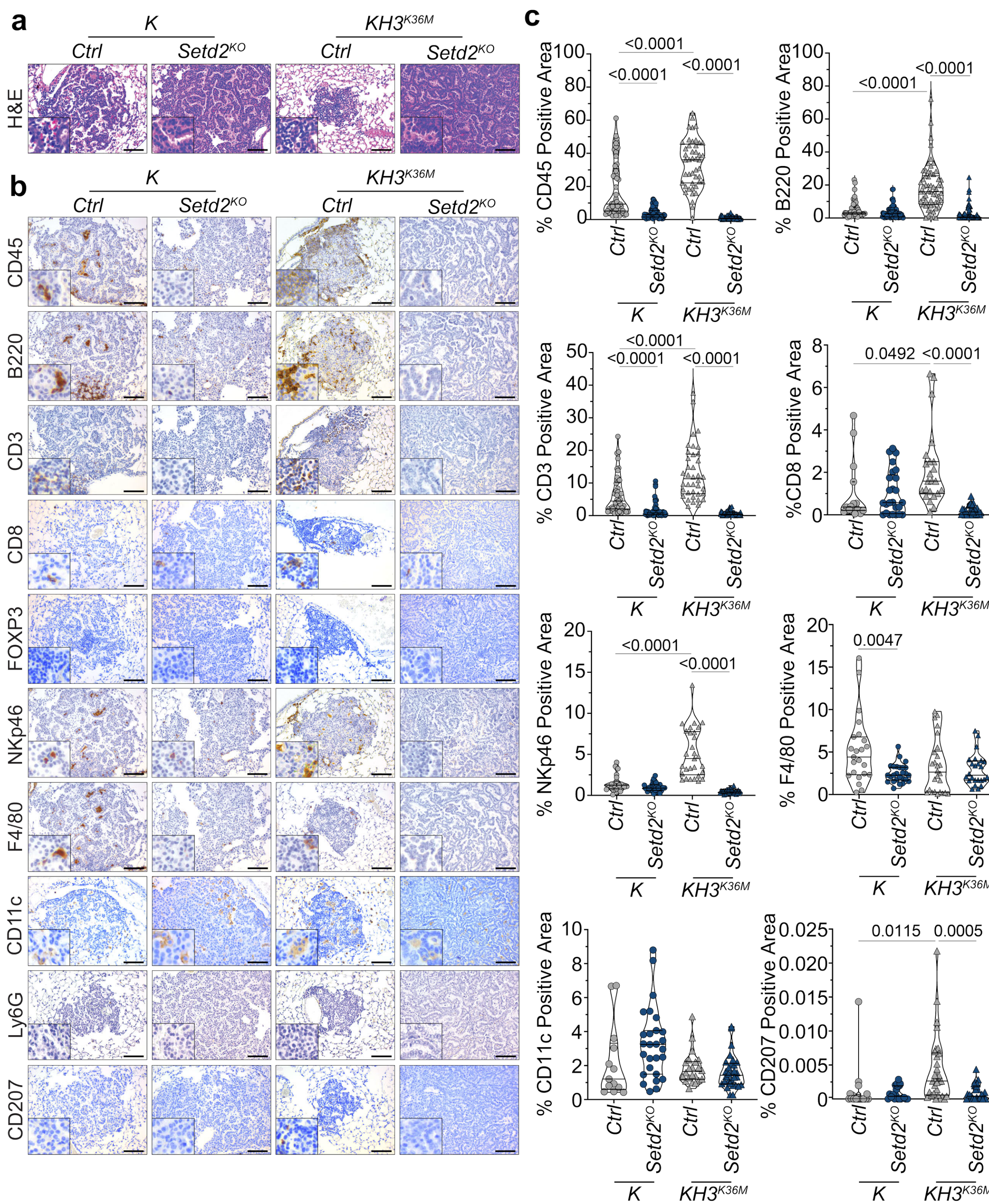

Supplementary Figure 2: H3^{K36M} promotes Setd2-dependent immune infiltration
a) Representative H&E images of tumors from *K-Ctrl*, *K-Setd2^{KO}*, *KH3^{K36M}-Ctrl*, and *KH3^{K36M}-Setd2^{KO}* mice. Scale bars are 119 µm. **b)** IHC for immune cell markers CD45, B220, CD3, CD8, FOXP3, NKp46, F4/80, CD11c, Ly6G, and CD207 in *K-Ctrl*, *K-Setd2^{KO}*, *KH3^{K36M}-Ctrl*, and *KH3^{K36M}-Setd2^{KO}* tumors. Scale bars are 119 µm; insets are magnified x3. **c)** Quantification of CD45, B220, CD3, CD8, NKp46, F4/80, CD11c, and CD207 IHC staining in *K-Ctrl*, *K-Setd2^{KO}*, *KH3^{K36M}-Ctrl*, and *KH3^{K36M}-Setd2^{KO}* mice. Sample sizes are reported in Source Data. Significance was determined by two-sided unpaired Student's t-test. Violin plots show the data distribution, with the center line indicating the median and the upper and lower lines indicating the interquartile range.

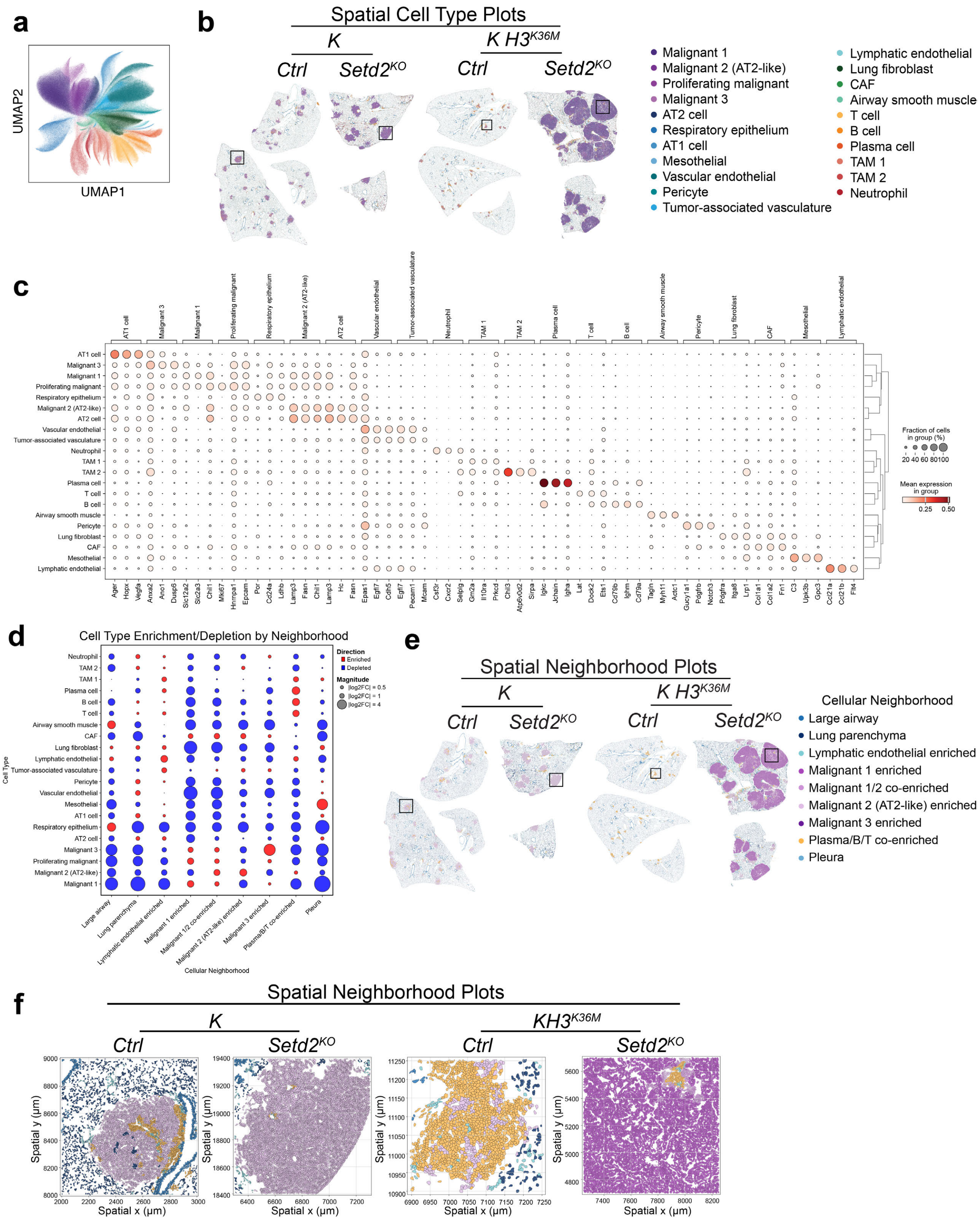

Supplementary Figure 3: Spatial transcriptomics identifies H3^{K36M}-promoted and Setd2-dependent immune infiltration and activation

a) Combined UMAP depicting cells from all four genotypes in **Fig. 2a**. **b)** Spatial cell type plots of full lung lobes used in spatial analysis, with boxes indicating representative tumors in **Fig. 2a**. **c)** Marker gene expression in cell types identified in Xenium spatial analysis. **d)** Fold change enrichment or depletion of cell types by cellular neighborhood. **e)** Spatial cellular neighborhood plots of full lung lobes used in spatial analysis, with boxes indicating representative tumors in **Fig. 2a**. **f)** Spatial cellular neighborhood plots of representative tumors from **Fig. 2a**.

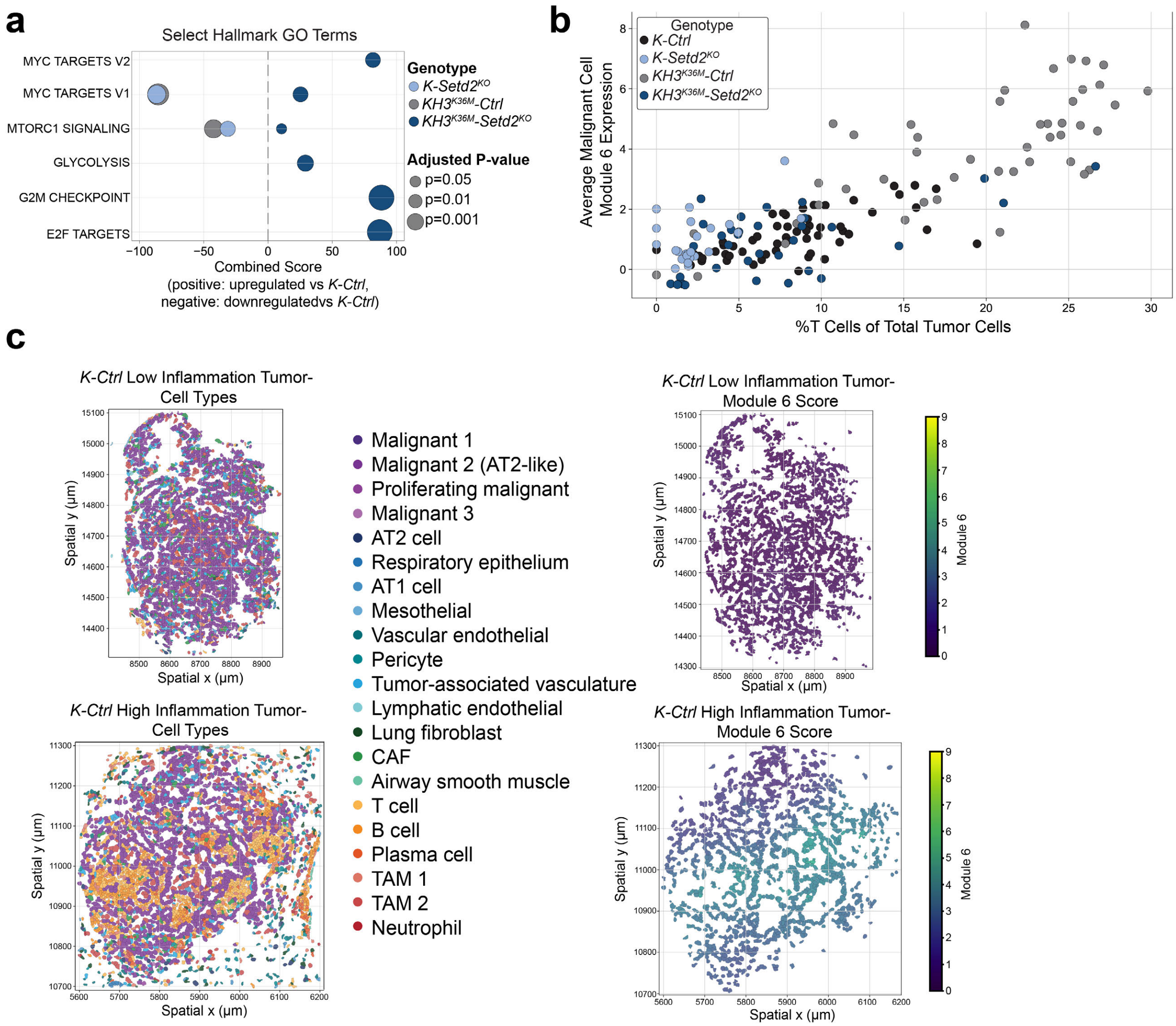

Supplementary Figure 4: Module 6 expression correlates with inflammation

a) Gene Ontology enrichment scores for GO Terms associated with increased tumor aggressiveness. Enrichment scores were calculated for the sets of differentially upregulated and downregulated genes in *K-Setd2*^{KO}, *KH3*^{K36M}*-Ctrl*, and *KH3*^{K36M}*-Setd2*^{KO} tumors compared to *K-Ctrl*. Enrichment for a GO Term in differentially upregulated genes is depicted on the positive x-axis while enrichment in downregulated genes is depicted on the negative x-axis. Non-significant enrichments are not plotted. **b)** Scatter plot depicting T cell infiltration and mean Module 6 (inflammatory module) score in malignant cells on a per-tumor basis, colored by genotype. **c)** Cell types and Module 6 scores in T-cell low and T-cell high *K-Ctrl* tumors.

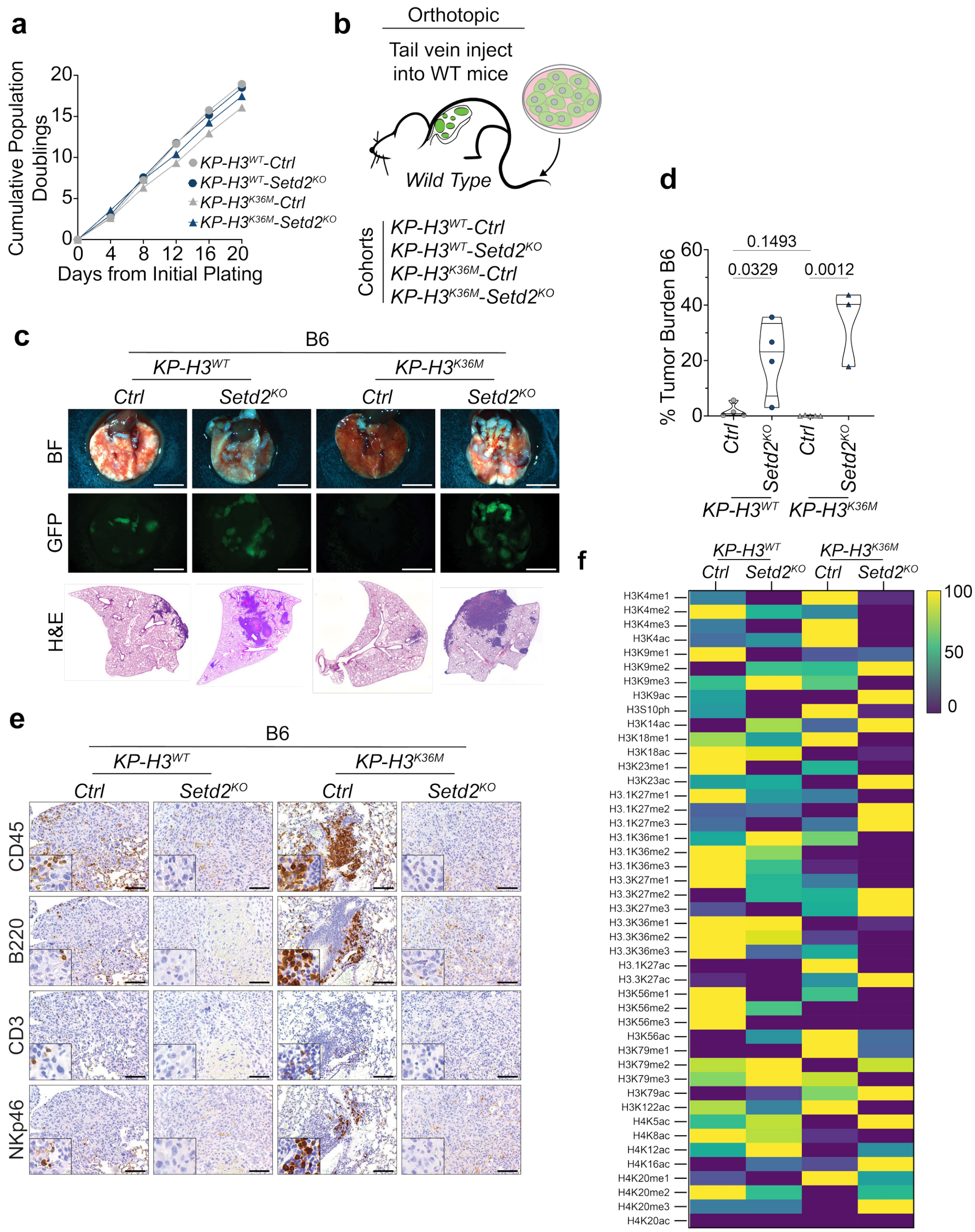

Supplementary Figure 5: H3^{K36M}-expressing cell lines phenocopy features of autochthonous tumors

a) Cell growth assay of *KP-H3^{WT}-Ctrl*, *KP-H3^{WT}-Setd2^{KO}*, *KP-H3^{K36M}-Ctrl*, and *KP-H3^{K36M}-Setd2^{KO}* cells. n=3 cell lines per group. **b)** Experimental scheme of orthotopic tumor initiation. **c)** Brightfield (BF) and fluorescent micrographs of lungs from C57BL/6J mice orthotopically injected with *KP-H3^{WT}-Ctrl*, *KP-H3^{WT}-Setd2^{KO}*, *KP-H3^{K36M}-Ctrl*, or *KP-H3^{K36M}-Setd2^{KO}* cells. Scale bars are 4.4 mm. Below are representative H&E images of tumor-bearing lobes from C57BL/6J mice orthotopically injected with *KP-H3^{WT}-Ctrl*, *KP-H3^{WT}-Setd2^{KO}*, *KP-H3^{K36M}-Ctrl*, or *KP-H3^{K36M}-Setd2^{KO}* cells. **d)** Quantification of tumor burden and individual tumor area in C57BL/6J mice orthotopically injected with *KP-H3^{WT}-Ctrl* (n=4 mice), *KP-H3^{WT}-Setd2^{KO}* (n=4 mice), *KP-H3^{K36M}-Ctrl* (n=5 mice), or *KP-H3^{K36M}-Setd2^{KO}* (n=3 mice) cells. Significance was determined by two-sided unpaired Student's t-test. Violin plots show the data distribution, with the center line indicating the median and the upper and lower lines indicating the interquartile range. **e)** Representative images of IHC for immune cell markers CD45, B220, CD3, and NKp46 in tumors from C57BL/6J mice orthotopically injected with *KP-H3^{WT}-Ctrl*, *KP-H3^{WT}-Setd2^{KO}*, *KP-H3^{K36M}-Ctrl*, or *KP-H3^{K36M}-Setd2^{KO}* cells. **f)** Relative abundance of histone modifications in *KP-H3^{WT}-Ctrl* (n=3 cell lines), *KP-H3^{WT}-Setd2^{KO}* (n=1 cell line), *KP-H3^{K36M}-Ctrl* (n=3 cell lines), and *KP-H3^{K36M}-Setd2^{KO}* (n=2 cell lines) cells by LC-MS/MS. Data are visualized in a row-normalized heat map.

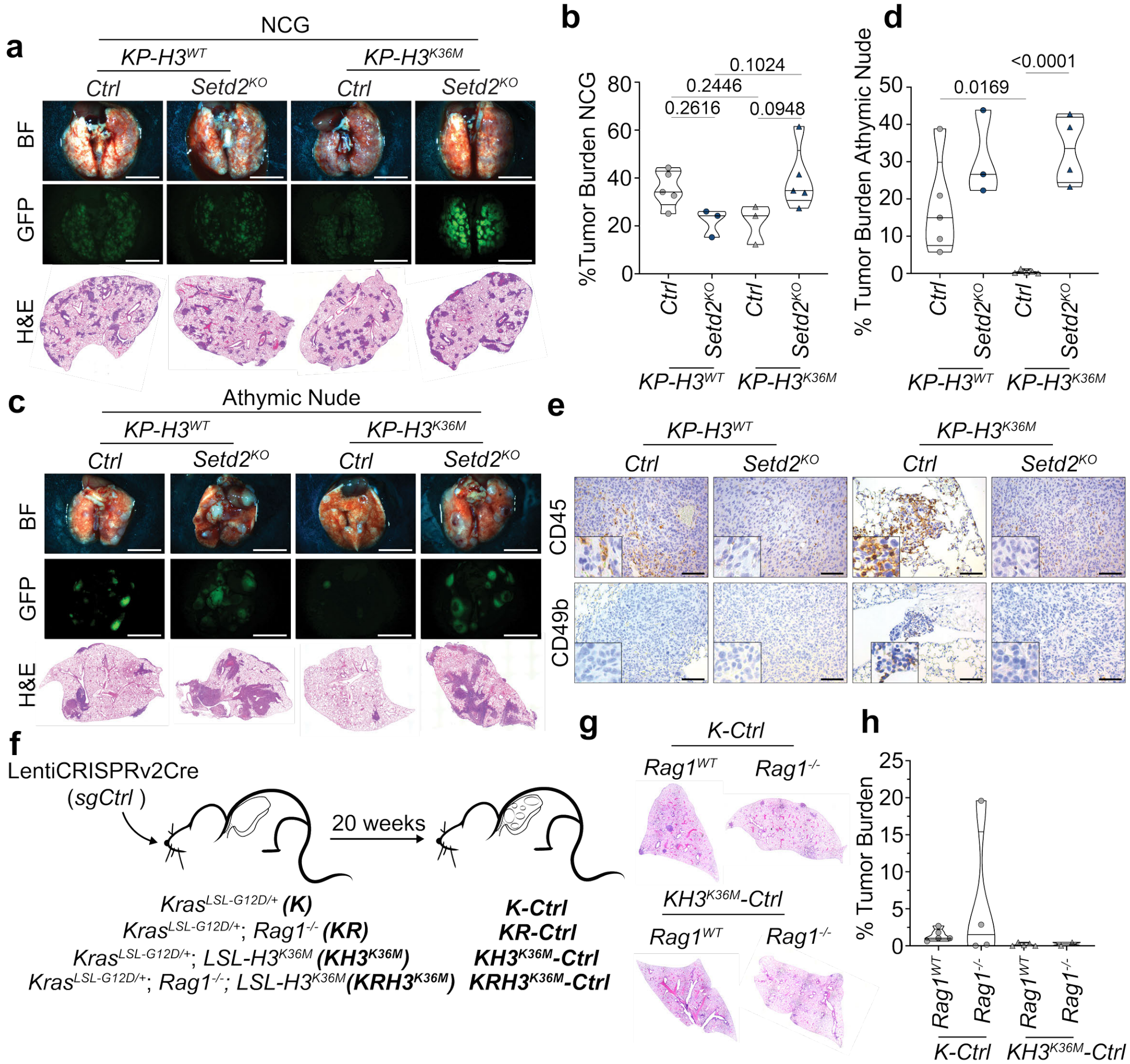

Supplementary Figure 6: NK cells are sufficient for H3^{K36M}-promoted tumor cell killing

a) Brightfield (BF) and fluorescent micrographs of lungs from NCG mice orthotopically injected with *KP-H3^{WT}-Ctrl*, *KP-H3^{WT}-Setd2^{KO}*, *KP-H3^{K36M}-Ctrl*, or *KP-H3^{K36M}-Setd2^{KO}* cells. Scale bars are 4.4 mm. Below are representative H&E images of tumor-bearing lobes from NCG mice orthotopically injected with *KP-H3^{WT}-Ctrl*, *KP-H3^{WT}-Setd2^{KO}*, *KP-H3^{K36M}-Ctrl*, or *KP-H3^{K36M}-Setd2^{KO}* cells. **b)** Quantification of tumor burden in NCG mice orthotopically injected with *KP-H3^{WT}-Ctrl* (n=5 mice), *KP-H3^{WT}-Setd2^{KO}* (n=3 mice), *KP-H3^{K36M}-Ctrl* (n=3 mice), or *KP-H3^{K36M}-Setd2^{KO}* (n=5 mice) cells. Significance was determined by two-sided unpaired Student's t-test. Violin plots show the data distribution, with the center line indicating the median and the upper and lower lines indicating the interquartile range. **c)** Brightfield (BF) and fluorescent micrographs of lungs from athymic nude mice orthotopically injected with *KP-H3^{WT}-Ctrl*, *KP-H3^{WT}-Setd2^{KO}*, *KP-H3^{K36M}-Ctrl*, or *KP-H3^{K36M}-Setd2^{KO}* cells. Scale bars are 4.4 mm. Below are representative H&E images of tumor-bearing lobes from athymic nude mice orthotopically injected with *KP-H3^{WT}-Ctrl*, *KP-H3^{WT}-Setd2^{KO}*, *KP-H3^{K36}M-Ctrl*, or *KP-H3^{K36M}-Setd2^{KO}* cells. **d)** Quantification of tumor burden in athymic nude mice orthotopically injected with *KP-H3^{WT}-Ctrl* (n=5 mice), *KP-H3^{WT}-Setd2^{KO}* (n=3 mice), *KP-H3^{K36M}-Ctrl* (n=5 mice), or *KP-H3^{K36M}-Setd2^{KO}* (n=4 mice) cells. Significance was determined by two-sided unpaired Student's t-test. Violin plots show the data distribution, with the center line indicating the median and the upper and lower lines indicating the interquartile range. **e)** IHC for immune cell markers CD45 and CD49b in tumors from athymic nude mice orthotopically injected with *KP-H3^{WT}-Ctrl*, *KP-H3^{WT}-Setd2^{KO}*, *KP-H3^{K36M}-Ctrl*, or *KP-H3^{K36M}-Setd2^{KO}* cells. Scale bars are 119 µm; insets are magnified x3. **f)** Experimental scheme of autochthonous tumor initiation in *K, KRag1$^{-/-}$, KH3^{K36M}, and KRag1$^{-/-}$H3^{K36M}* mice. **g)** Representative images of tumor-bearing lobes from *K-Ctrl, KRag1$^{-/-}$-Ctrl, KH3^{K36M}-Ctrl, and KRag1$^{-/-}$H3^{K36M}-Ctrl* mice. **h)** Quantification of tumor burden in *K-Ctrl* (n=5 mice), *KRag1$^{-/-}$-Ctrl* (n=4 mice), *KH3^{K36M}-Ctrl* (n=5 mice), *and KRag1$^{-/-}$H3^{K36M}-Ctrl* (n=2 mice) mice. Significance was determined by two-sided unpaired Student's t-test. Violin plots show the data distribution, with the center line indicating the median and the upper and lower lines indicating the interquartile range.

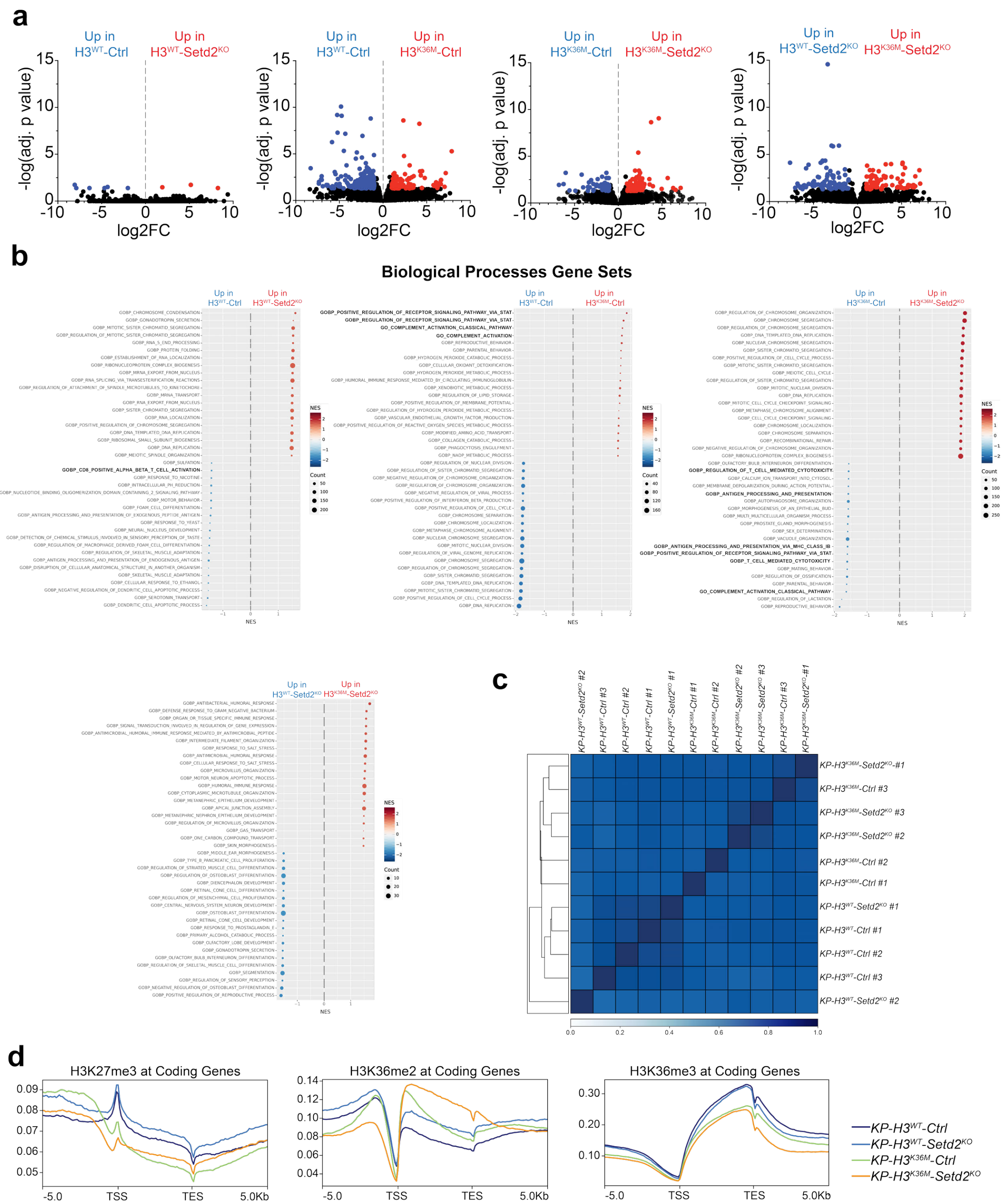

Supplementary Figure 7: H3^{K36M} induces immune-related gene expression programs and remodels chromatin methylation. **a)** Volcano plots of RNA-sequencing data from *KP-H3^{WT}-Ctrl* , *KP-H3^{WT}-Setd2^{KO}*, *KP-H3^{K36M}-Ctrl*, and *KP-H3^{K36M}-Setd2^{KO}* cells. n=3 cell lines per group. Comparisons are as listed. Colored dots represent genes that are differentially enriched (log2 fold-change greater-than 1 and false discovery rate (FDR)-adjusted P-value less-than 0.05). **b)** Bubble plot representation of top 20 up- and down-regulated GSEA biological processes gene sets in the noted comparisons. Normalized enrichment score (NES) plotted and the size of each dot represents the number of genes within the gene set. **c)** Correlation heatmap with hierarchical clustering amongst *KP-H3^{WT}-Ctrl*, *KP-H3^{WT}-Setd2^{KO}*, *KP-H3^{K36M}-Ctrl*, and *KP-H3^{K36M}-Setd2^{KO}* cell lines. **d)** Metagene plots describing the density of H3K27me3, H3K36me2, and H3K36me3 ChIP-seq across all coding genes. Plots are centered on coding gene bodies across a ± 5 kb window.

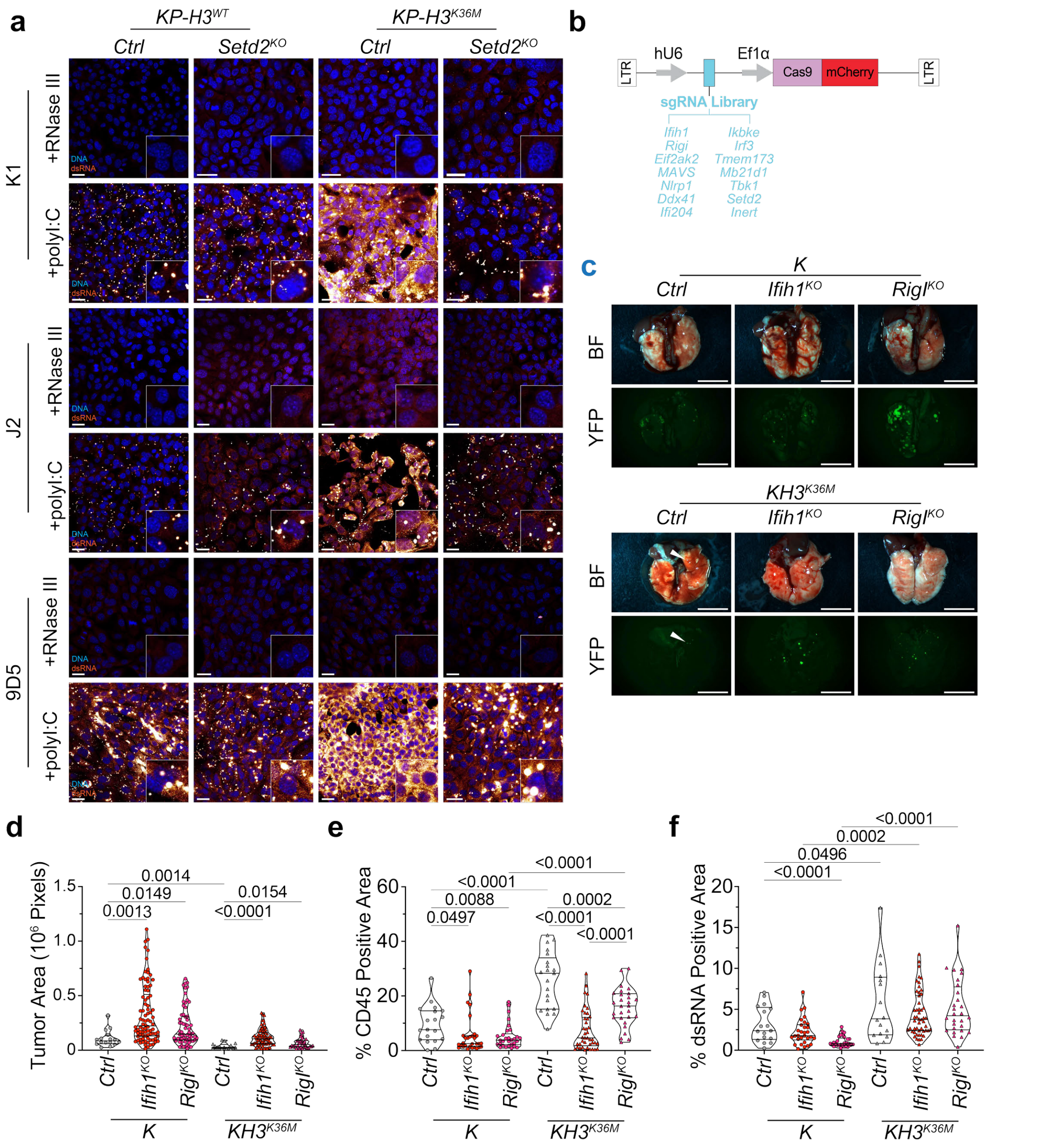

Supplementary Figure 8: H3^{K36M}-induced dsRNA signal is specific

a) Immunocytochemistry for dsRNA in *KP-H3^{WT}-Ctrl*, *KP-H3^{WT}-Setd2^{KO}*, *KP-H3^{K36M}-Ctrl*, and *KP-H3^{K36M}-Setd2^{KO}* cells. Staining was performed in parallel using 3 dsRNA antibodies: K1, J2, and 9D5. Cells received RNase III treatment or were transfected with polyI:C. **b)** Schematic of plasmid used for CRISPR enrichment screen targeting dsRNA sensing pathway genes. **c)** Brightfield (BF) and fluorescent micrographs of lungs from *K-Ctrl*, *K-Ifih1^{KO}*, *K*-RigI^{KO}, *K-H3^{K36M}-Ctrl*, *K-H3^{K36M}-Ifih1^{KO}*, and *K-H3^{K36M}-RigI^{KO}* mice. Scale bars are 4.4 mm. **d)** Quantification of tumor area in *K-Ctrl* (n=16 tumors), *K-Ifih1^{KO}* (n=86 tumors), *K-RigI^{KO}* (n=64 tumors), *K-H3^{K36M}-Ctrl* (n=16 tumors), *K-H3^{K36M}-Ifih1^{KO}* (n=75 tumors), and *K-H3^{K36M}-RigI^{KO}* (n=75 tumors) mice. Significance was determined by two-sided unpaired Student's t-test. Violin plots show the data distribution, with the center line indicating the median and the upper and lower lines indicating the interquartile range. **e)** Quantification of CD45 IHC staining in *K-Ctrl* (n=19 tumors), *K-Ifih1^{KO}* (n=30 tumors), *K-RigI^{KO}* (n=30 tumors), *K-H3^{K36M}-Ctrl* (n=21 tumors), *K-H3^{K36M}-Ifih1^{KO}* (n=41 tumors), and *K-H3^{K36M}-RigI^{KO}* (n=28 tumors) mice. Significance was determined by two-sided unpaired Student's t-test. Violin plots show the data distribution, with the center line indicating the median and the upper and lower lines indicating the interquartile range. **f)** Quantification of dsRNA IHC staining in *K-Ctrl* (n=18 tumors), *K-Ifih1^{KO}* (n=30 tumors), *K-RigI^{KO}* (n=30 tumors), *K-H3^{K36M}-Ctrl* (n=16 tumors), *K-H3^{K36M}-Ifih1^{KO}* (n=39 tumors), and *K-H3^{K36M}-RigI^{KO}* (n=29 tumors) mice. Significance was determined by two-sided unpaired Student's t-test. Violin plots show the data distribution, with the center line indicating the median and the upper and lower lines indicating the interquartile range.

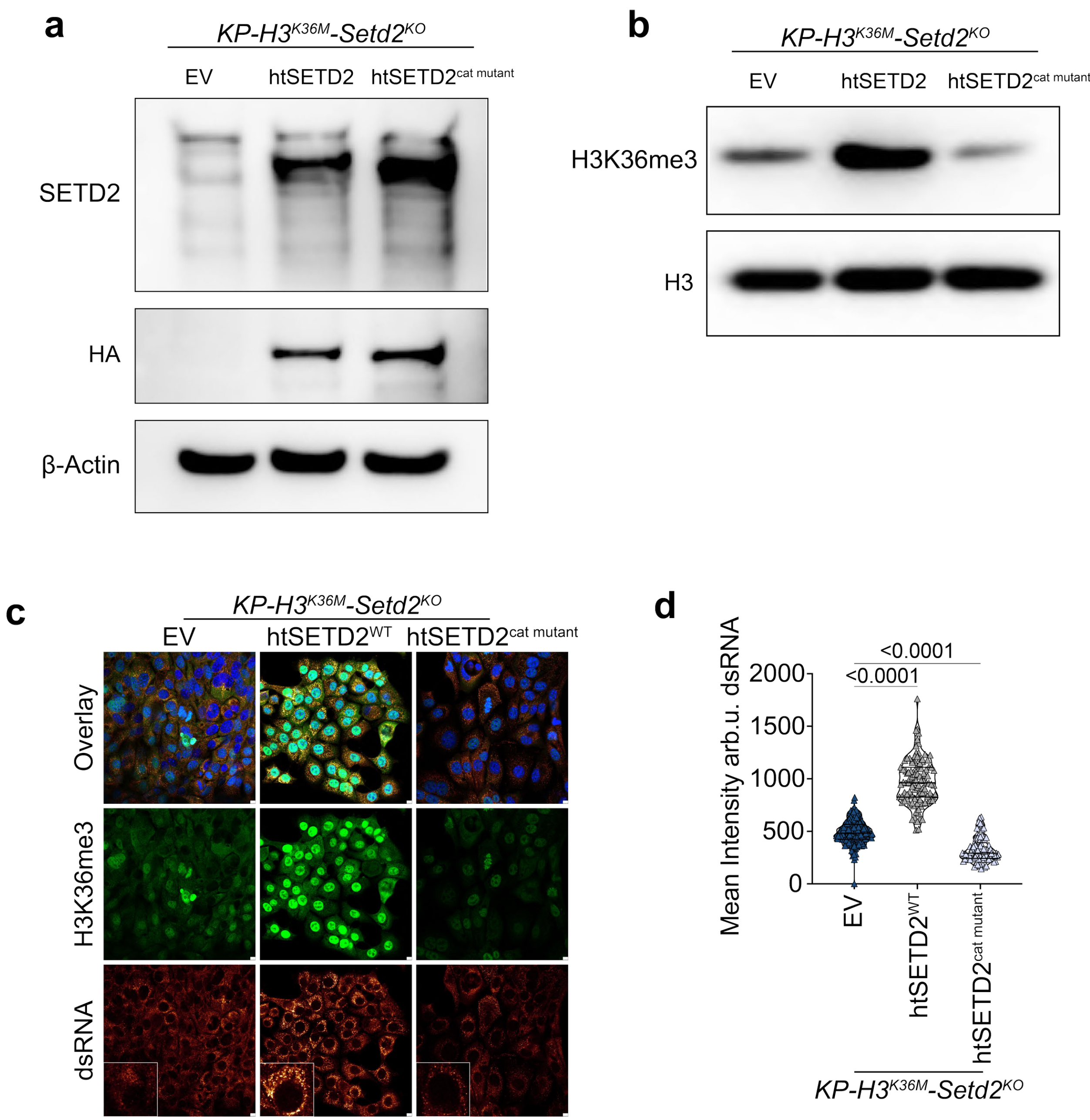

Supplementary Figure 9: **The methyltransferase activity of SETD2 is required for dsRNA production**

a) Immunoblot analysis of SETD2, HA, and β-Actin in lysates prepared from *KP-H3^{K36M}-Setd2^{KO}* cells expressing either empty vector (EV), htSETD2^{WT}, or htSETD2$^{cat\ mutant}$ constructs. β-Actin is a loading control. **b)** Immunoblot analysis of H3K36me3 and H3 in histones prepared from *KP-H3^{K36M}-Setd2^{KO}* cells expressing either empty vector (EV), htSETD2^{WT}, or htSETD2$^{cat\ mutant}$ constructs. H3 is a loading control. **c)** Immunocytochemistry for dsRNA and H3K36me3 in *KP-H3^{K36M}-Setd2^{KO}* cells expressing either empty vector (EV), htSETD2^{WT}, or htSETD2$^{cat\ mutant}$ constructs. Scale bars are 5 µm; insets are magnified x3. **d)** Quantification of dsRNA intensity. Significance was determined by two-sided unpaired Student's t-test. Violin plots show the data distribution, with the center line indicating the median and the upper and lower lines indicating the interquartile range.

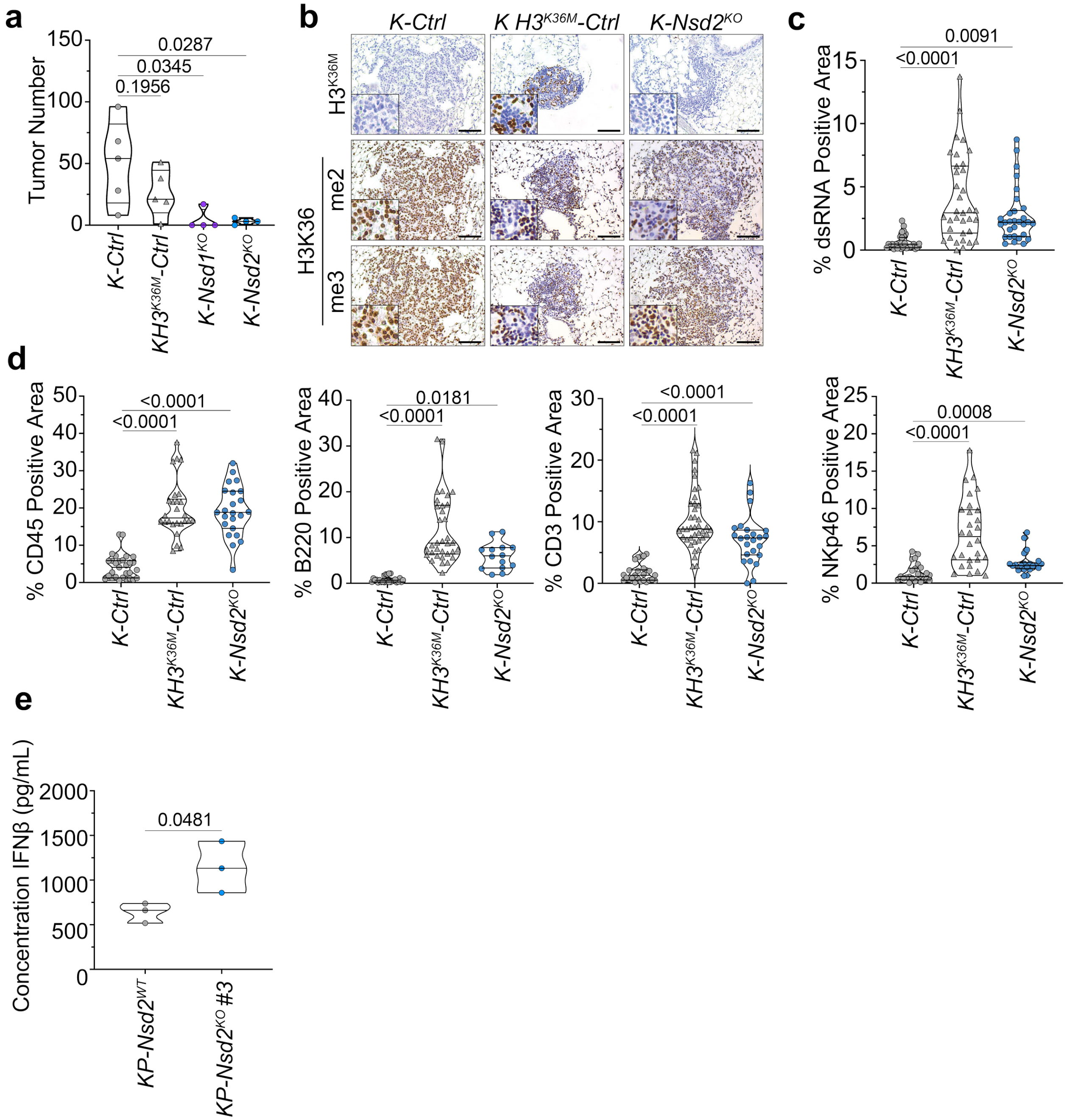

Supplementary Figure 10: NSD2 promotes immune evasion in lung adenocarcinoma.
a) Quantification of tumor number in *K-Ctrl* (n=5 mice), *$KH3^{K36M}$-Ctrl* (n=5 mice), *K-$Nsd1^{KO}$* (n=4 mice), and *K-$Nsd2^{KO}$* mice (n=4 mice) mice. Significance was determined by two-sided unpaired Student's t-test. Violin plots show the data distribution, with the center line indicating the median and the upper and lower lines indicating the interquartile range. **b)** IHC for $H3^{K36M}$, H3K36me2, and H3K36me3 in *K-Ctrl*, *$KH3^{K36M}$-Ctrl*, *K-$Nsd1^{KO}$*, and *K-$Nsd2^{KO}$* mice*.* Scale bars are 119 µm; insets are magnified x3. **c)** Quantification of dsRNA IHC staining in *K-Ctrl* (n=30 tumors), *$KH3^{K36M}$-Ctrl* (n=35 tumors), and *K-$Nsd2^{KO}$* (n=30 tumors) mice. Significance was determined by two-sided unpaired Student's t-test. Violin plots show the data distribution, with the center line indicating the median and the upper and lower lines indicating the interquartile range. **d)** Quantification of CD45, B220, CD3, and NKp46 IHC staining in *K-Ctrl*, *$KH3^{K36M}$-Ctrl*, and *K-$Nsd2^{KO}$* mice. Sample sizes are reported in Source Data. Significance was determined by two-sided unpaired Student's t-test. Violin plots show the data distribution, with the center line indicating the median and the upper and lower lines indicating the interquartile range. **e)** IFN-β ELISA analysis of cell culture supernatant collected from *KP-$Nsd2^{WT}$* and *KP-$Nsd2^{KO}$* cells (n=3). Significance was determined by two-sided unpaired Student's t-test. Violin plots show the data distribution, with the center line indicating the median and the upper and lower lines indicating the interquartile range.

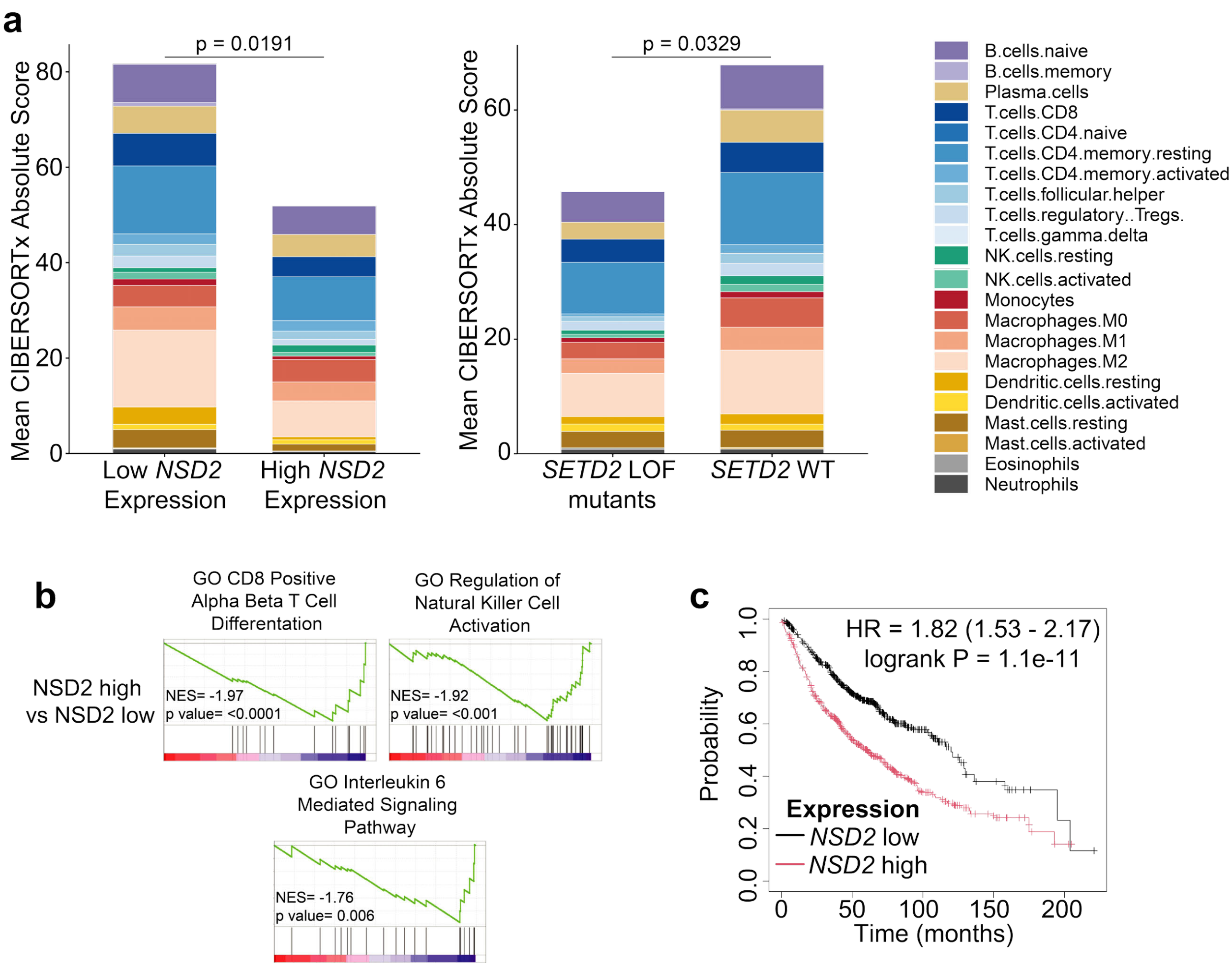

Supplementary Figure 11: Low NSD2 expression in human lung adenocarcinoma is correlated with high immune infiltration and better patient outcomes.
a) CIBERSORT analysis of RNA-seq data from lung adenocarcinoma patients with high and low *NSD2* expression or wildtype SETD2 and SETD2 loss of function mutations. **b)** Select immune-related gene sets that are de-enriched in human lung adenocarcinoma patients with high *NSD2* expression. **c)** Kaplan–Meier survival curves for lung adenocarcinoma patients with tumors expressing low (*n* = 581) or high (*n* = 580) levels of *NSD2*.

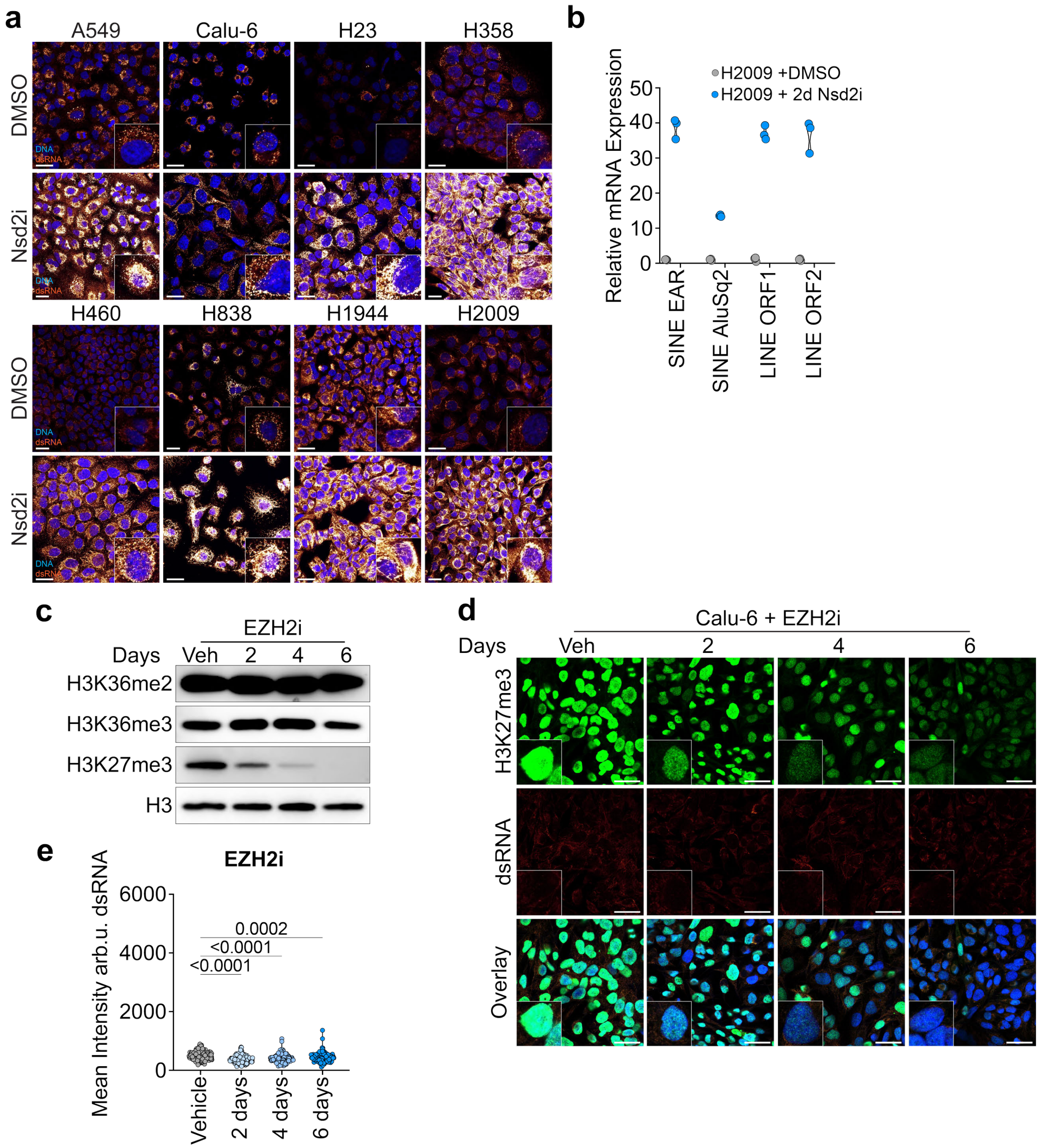

Supplementary Figure 12: NSD2 inhibition promotes ERV expression and dsRNA formation.
a) Immunocytochemistry for K1 dsRNA in human cell lines treated with NSD2 inhibitor for 48 hours. Scale bars are 25 μm; insets are magnified x3. **b)** Quantitative real-time PCR for LINE and SINE element expression. Element expression is normalized to 5srRNA expression and is relative to expression of these elements in untreated H2009 cells. n=3 replicates per group. **c)** Immunoblot analysis of H3K36me2, H3K36me3, H3K27me3 and H3 in histones prepared from Calu-6 cells treated with EZH2 inhibitor for 2, 4, or 6 days. Veh marks Calu-6 cells treated with vehicle (DMSO) for 6 days. H3 is a loading control. **d)** Immunocytochemistry for K1 dsRNA in Calu-6 cells treated with EZH2 inhibitor for 2, 4, or 6 days. Veh marks Calu-6 cells treated with vehicle (DMSO) for 6 days. Scale bars are 25 μm; insets are magnified x3. **e)** Quantification of dsRNA intensity in Calu-6 cells treated with NSD2 inhibitor for 2, 4, or 6 days. Significance was determined by two-sided unpaired Student's t-test. Violin plots show the data distribution, with the center line indicating the median and the upper and lower lines indicating the interquartile range.

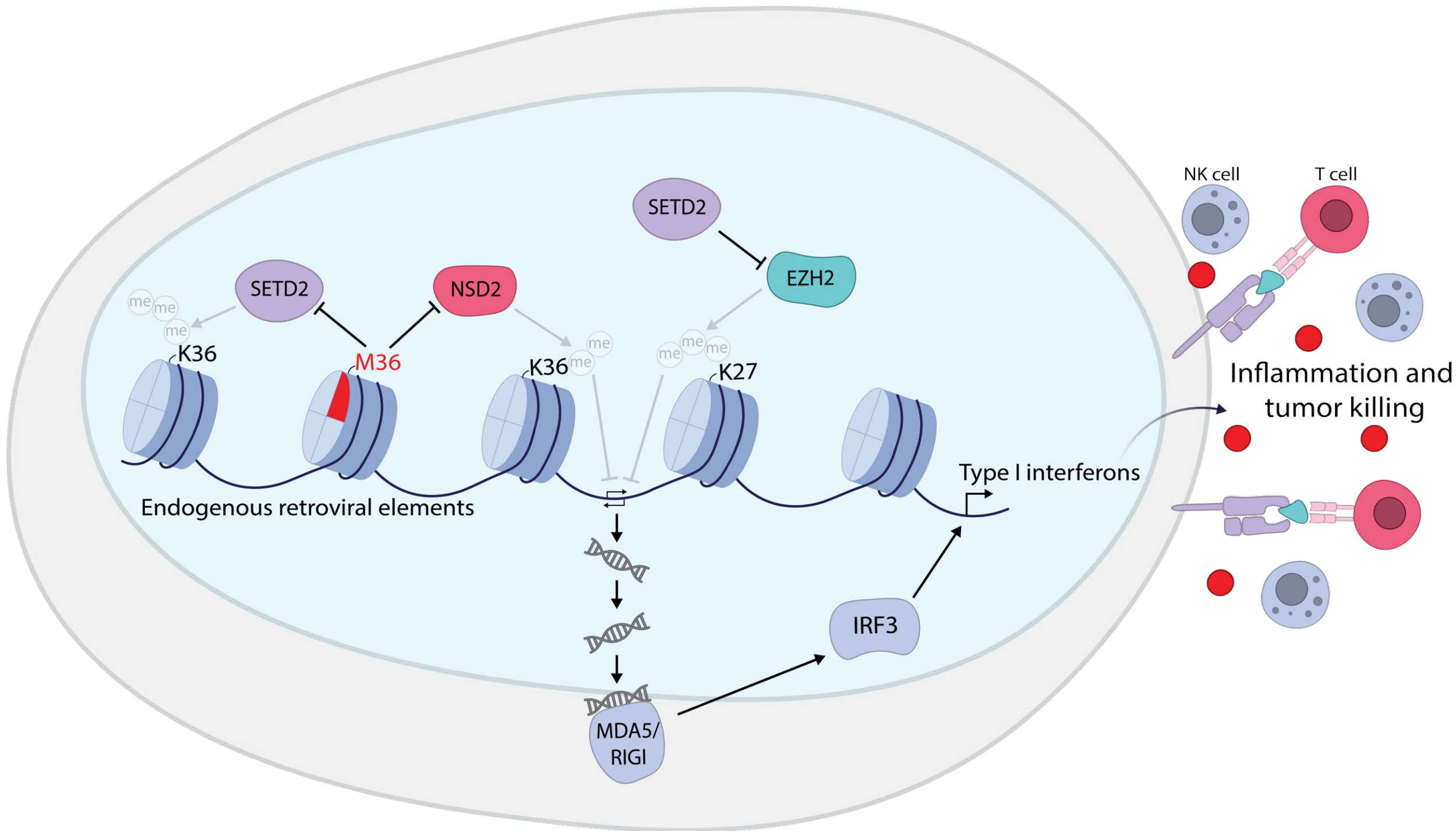

Supplementary Figure 13: Loss of H3K36 dimethylation induces dsRNA expression that promotes Setd2-dependent, immune-mediated tumor killing.
Expression of $H3^{K36M}$ in tumors inhibits H3K36 methyltransferases SETD2 and NSD2. Loss of NSD2-mediated H3K36me2 reduces H3K27me3 at endogenous retroviral elements (ERVs), leading to dsRNA accumulation and activation of RIG-I/MDA5-dependent antiviral immunity that drives tumor cell clearance. In Setd2-deficient contexts, EZH2-dependent H3K27me3 restores ERV repression, prevents dsRNA accumulation, and enables immune escape.
