# Supplementary material for: Antagonistic roles of histone H3 lysine methyltransferases dictate tumor immune surveillance
Source: Nat Commun. 2026 Sep 14;17:10810. doi: 10.1038/s41467-026-76657-3 (PMC13669384; doi:10.1038/s41467-026-76657-3)
Supplement: Supplementary file 2 [file 41467_2026_76657_MOESM2_ESM.pdf]

Description of Additional Supplementary Files

Supplementary Dataset 1: List of genes comprising the Hotspot gene modules identified from Xenium spatial transcriptomic analysis.

Supplementary Dataset 2: sgRNA sequences used for CRISPR-mediated gene inactivation.

Supplementary Dataset 3: Oligonucleotide primer sequences used for cloning human SETD2 constructs.

Supplementary Dataset 4: RT-qPCR primer sequences.
