# Supplementary material for: Antagonistic roles of histone H3 lysine methyltransferases dictate tumor immune surveillance
Source: Nat Commun. 2026 Sep 14;17:10810. doi: 10.1038/s41467-026-76657-3 (PMC13669384; doi:10.1038/s41467-026-76657-3)
Supplement: Supplementary file 3 [file 41467_2026_76657_MOESM7_ESM.pdf]

nature portfolio

Corresponding author(s): David Feldser

Last updated by author(s): Jun 8, 2026

Reporting Summary

Nature Portfolio wishes to improve the reproducibility of the work that we publish. This form provides structure for consistency and transparency in reporting. For further information on Nature Portfolio policies, see our Editorial Policies and the Editorial Policy Checklist.

Statistics

For all statistical analyses, confirm that the following items are present in the figure legend, table legend, main text, or Methods section.

n/a	Confirmed	
☐	☒	The exact sample size (*n*) for each experimental group/condition, given as a discrete number and unit of measurement
☐	☒	A statement on whether measurements were taken from distinct samples or whether the same sample was measured repeatedly
☐	☒	The statistical test(s) used AND whether they are one- or two-sided<br>*Only common tests should be described solely by name; describe more complex techniques in the Methods section.*
☒	☐	A description of all covariates tested
☒	☐	A description of any assumptions or corrections, such as tests of normality and adjustment for multiple comparisons
☐	☒	A full description of the statistical parameters including central tendency (e.g. means) or other basic estimates (e.g. regression coefficient) AND variation (e.g. standard deviation) or associated estimates of uncertainty (e.g. confidence intervals)
☐	☒	For null hypothesis testing, the test statistic (e.g. *F*, *t*, *r*) with confidence intervals, effect sizes, degrees of freedom and *P* value noted<br>*Give P values as exact values whenever suitable.*
☒	☐	For Bayesian analysis, information on the choice of priors and Markov chain Monte Carlo settings
☒	☐	For hierarchical and complex designs, identification of the appropriate level for tests and full reporting of outcomes
☒	☐	Estimates of effect sizes (e.g. Cohen's *d*, Pearson's *r*), indicating how they were calculated

Our web collection on statistics for biologists contains articles on many of the points above.

Software and code

Policy information about availability of computer code

Data collection	No custom or commercial code was used to collect data
Data analysis	ImageJ was used for histological assessments and quantifications. Quantitative and statistical analyses were conducted using GraphPad Prism 10. Xenium analyses were performed in Python 3.11 using Scanpy v1.10.3, AnnData v0.10.9, and Hotspot v1.1.1. UMAP visualization was performed using umap-learn v0.5.6, and gene set enrichment analysis was performed using GSEApy v1.1.8. Code used in this study for the Xenium analysis is available at GitHub (https://github.com/cdpolt/Gladstein-et-al-Xenium) CRISPR screen sequencing data were analyzed using MAGeCK v0.5.9.5. Software used for peak calling for ChIP-seq is described below. For RNA-seq Salmon was used for transcript quantification. Transcript abundance estimates were imported using tximport, and differential expression analysis was performed using DESeq2. Gene set enrichment analysis was performed using GSEA with MSigDB gene sets. Heatmaps and data visualization were generated using pheatmap and ggplot2. Additional analyses were performed using ashr, rtracklayer, and Snakemake. For ATAC seq, reads were processed using Atria for adapter trimming and Bowtie2 v2.5.1 for alignment to the mouse reference genome (mm39/GRCm39). Peak calling was performed using Genrich. Signal tracks were generated and sample correlations were assessed using deepTools (bamCoverage, bigWigSummary, and plotCorrelation).

For manuscripts utilizing custom algorithms or software that are central to the research but not yet described in published literature, software must be made available to editors and reviewers. We strongly encourage code deposition in a community repository (e.g. GitHub). See the Nature Portfolio guidelines for submitting code & software for further information.

Data

Policy information about availability of data

All manuscripts must include a data availability statement. This statement should provide the following information, where applicable:

- Accession codes, unique identifiers, or web links for publicly available datasets
- A description of any restrictions on data availability
- For clinical datasets or third party data, please ensure that the statement adheres to our policy

All sequencing data generated in this study have been deposited in the NCBI Gene Expression Omnibus (GEO) database under accession codes GSE335211 (Xenium), GSE341379 (RNA-, ATAC-, and ChIP-sequencing), and GSE334916 (CRISPR enrichment screen). The mass spectrometry raw files were uploaded in the online repository ProteomeXchange (PRIDE) under the project number PXD069619. Source data are provided with this paper.

Research involving human participants, their data, or biological material

Policy information about studies with human participants or human data. See also policy information about sex, gender (identity/presentation), and sexual orientation and race, ethnicity and racism.

Reporting on sex and gender	*Use the terms sex (biological attribute) and gender (shaped by social and cultural circumstances) carefully in order to avoid confusing both terms. Indicate if findings apply to only one sex or gender; describe whether sex and gender were considered in study design; whether sex and/or gender was determined based on self-reporting or assigned and methods used. Provide in the source data disaggregated sex and gender data, where this information has been collected, and if consent has been obtained for sharing of individual-level data; provide overall numbers in this Reporting Summary. Please state if this information has not been collected. Report sex- and gender-based analyses where performed, justify reasons for lack of sex- and gender-based analysis.*
Reporting on race, ethnicity, or other socially relevant groupings	*Please specify the socially constructed or socially relevant categorization variable(s) used in your manuscript and explain why they were used. Please note that such variables should not be used as proxies for other socially constructed/relevant variables (for example, race or ethnicity should not be used as a proxy for socioeconomic status). Provide clear definitions of the relevant terms used, how they were provided (by the participants/respondents, the researchers, or third parties), and the method(s) used to classify people into the different categories (e.g. self-report, census or administrative data, social media data, etc.) Please provide details about how you controlled for confounding variables in your analyses.*
Population characteristics	*Describe the covariate-relevant population characteristics of the human research participants (e.g. age, genotypic information, past and current diagnosis and treatment categories). If you filled out the behavioural & social sciences study design questions and have nothing to add here, write "See above."*
Recruitment	*Describe how participants were recruited. Outline any potential self-selection bias or other biases that may be present and how these are likely to impact results.*
Ethics oversight	*Identify the organization(s) that approved the study protocol.*

Note that full information on the approval of the study protocol must also be provided in the manuscript.

Field-specific reporting

Please select the one below that is the best fit for your research. If you are not sure, read the appropriate sections before making your selection.

☒ Life sciences ☐ Behavioural & social sciences ☐ Ecological, evolutionary & environmental sciences

For a reference copy of the document with all sections, see nature.com/documents/nr-reporting-summary-flat.pdf

Life sciences study design

All studies must disclose on these points even when the disclosure is negative.

Sample size	Commonly, we tried to randomize ~10 mice into each group. Given the heterogeneity in tumors, combined with the number of mice, we found adequate power for analysis.
Data exclusions	Data was not excluded.
Replication	Animals were enrolled into in vivo experiments as they became available until sufficient numbers were obtained to determine experimental outcomes.
Randomization	Allocation of mice into experimental groups was random.
Blinding	Investigators were not blinded when conducting data collection.

Reporting for specific materials, systems and methods

We require information from authors about some types of materials, experimental systems and methods used in many studies. Here, indicate whether each material, system or method listed is relevant to your study. If you are not sure if a list item applies to your research, read the appropriate section before selecting a response.

Materials & experimental systems

n/a	Involved in the study
☐	☒ Antibodies
☐	☒ Eukaryotic cell lines
☒	☐ Palaeontology and archaeology
☐	☒ Animals and other organisms
☒	☐ Clinical data
☒	☐ Dual use research of concern
☒	☐ Plants

Methods

n/a	Involved in the study
☐	☒ ChIP-seq
☒	☐ Flow cytometry
☒	☐ MRI-based neuroimaging

Antibodies

Antibodies used

Primary antibodies used were:

For immunohistochemistry:
H3K36M (1:2500, Cayman Chemical Company 31547), Ki67 (1:800, Novus Biologicals NB110-89717), pH3 (1:500, Cell Signaling Technology cs9701), Nkx2.1 (abcam ab76013), CD3 (1:700, abcam ab5690), SPC(1:2000, Millipore AB3786), CD45 (1:100, BioLegend 103102), B220 (1:50, BD Biosciences 551459), NKp46 (1:100, BioLegend 137602), F4/80 (1:500, Bio-Rad MCA497GA), Ly6G (1:500 Biolegend), CD8 (1:200, Cell Signaling Technology 98941S), CD11c (1:500, Cell Signaling Technology 97585S), CD207 (1:500, Invitrogen MA562917), and FoxP3 (1:200, Cell Signaling Technology 12653S), CD49b (1:500, Thermo Fisher 14-5971-85), and J2 dsRNA (1:500, Cell Signaling Technology 76651).

For immunoblotting:
Histones were blotted for H3K36M (1:1000, Cayman Chemical Company 31547), H3K36me3 (1:1000, Abcam ab9050), H3K36me2 (1:1000 Abcam ab9049), H3K27me3 (1:1000, Cell Signaling Technology 9733), H3 (1:20000, Abcam ab1791), and H2B (1:20000, Abcam ab1790). Whole cell lysates were blotted for NSD2 (1:1000, Abcam, ab75359), SETD2 (1:1000, Cell Signaling Technology 80290), HA-Tag (1:1000, Cell Signaling Technology 3724) and β-actin (1:10000, Sigma Aldrich, A2066).

For immunocytochemistry:
anti-dsRNA antibodies K1 (1:800, Cell Signaling Technology 28764), J2 (1:500, Cell Signaling Technology 76651), or 9D5 (1:500, Ab00458-23.0), anti-H3K27me3 antibody (1:800, Cell Signaling Technology 9733). anti-rabbit IgG Alexa Fluor 594 (1:200, Thermo Fisher A32754) or anti-mouse biotinylated secondary antibody (1:200, Vector Laboratories BA-9200). antistrepavidin Alexa Fluor 594 (1:200, Thermo Fisher S32356) or antistrepavidin Alexa Fluor 647 (1:200, Thermo Fisher S32357)

For ChIP:
H3K36me2 (5 µg, abcam, ab9049), H3K36me3 (5 µg, abcam, ab9050), H3K27me3 (5 µg, Cell Signaling Technologies, 9733T), or mouse IgG (1 µg, Diagenode, C15400001-15)

Validation

All antibodies were used in accordance to the manufacturer guidelines and have been well documented in the literature.

Eukaryotic cell lines

Policy information about cell lines and Sex and Gender in Research

Cell line source(s)

Human cell lines were obtained from the NCI cell line repository. Mouse LG1233 cell line was provided by Dr. Tyler Jacks (Massachusetts Institute of Technology, Cambridge, USA).

Authentication

Human cell lines were obtained from the NCI cell line repository and used in accordance to manufacturer guidelines. Mouse cell line has been well characterized.

Mycoplasma contamination

All human cell lines were tested by the NCI cell line repository and subsequently tested by our laboratory. The mouse cell line was tested after receipt.

Commonly misidentified lines
(See ICLAC register)

We have not utilized any misidentified cell lines in our study.

Animals and other research organisms

Policy information about studies involving animals; ARRIVE guidelines recommended for reporting animal research, and Sex and Gender in Research

Laboratory animals	All mice used were Mus musculus species.<br><br>Autochthonous model:<br>Equal numbers of male and female C57BL/6J were used. Tumors were initiated by 20 weeks of age.<br>Transplant models:<br>For the orthotropic lung adenocarcinoma mouse model, Rosa26-LSL-Cas9 mice (called C57BL/6J mice, Jax stock number 026175), OD-Prkdcem26Cd52; Il2rgem26Cd22 mice (NCG, Taconic strain 572), and CrTac: NCr-Foxn1nu mice (athymic nude, Taconic strain NCRNU) have been previously described. 50,000 cells were injected into the tail veins of these mice at ~4-5 weeks of age. For C57BL/6J, NCG, and athymic nude mice, tumors formed for 5, 2, or 3 weeks, respectively. Only male NCG and athymic nude mice were used.
Wild animals	Study did not involve wild animals.
Reporting on sex	Authochthnous mouse experiments were sex balanced, to the best of our ability. Transplant models were all male mice.
Field-collected samples	Our study did not involve field-collected samples.
Ethics oversight	Our animal protocol was approved by the University Laboratory Animal Resources (ULAR) at the University of Pennsylvania and the IACUC.

Note that full information on the approval of the study protocol must also be provided in the manuscript.

Plants

Seed stocks	*Report on the source of all seed stocks or other plant material used. If applicable, state the seed stock centre and catalogue number. If plant specimens were collected from the field, describe the collection location, date and sampling procedures.*
Novel plant genotypes	*Describe the methods by which all novel plant genotypes were produced. This includes those generated by transgenic approaches, gene editing, chemical/radiation-based mutagenesis and hybridization. For transgenic lines, describe the transformation method, the number of independent lines analyzed and the generation upon which experiments were performed. For gene-edited lines, describe the editor used, the endogenous sequence targeted for editing, the targeting guide RNA sequence (if applicable) and how the editor was applied.*
Authentication	*Describe any authentication procedures for each seed stock used or novel genotype generated. Describe any experiments used to assess the effect of a mutation and, where applicable, how potential secondary effects (e.g. second site T-DNA insertions, mosiacism, off-target gene editing) were examined.*

ChIP-seq

Data deposition

☒ Confirm that both raw and final processed data have been deposited in a public database such as GEO.

☒ Confirm that you have deposited or provided access to graph files (e.g. BED files) for the called peaks.

Data access links<br>*May remain private before publication.*	https://dataview.ncbi.nlm.nih.gov/object/PRJNA1266719?reviewer=8f75c61n9g19s7mjcd1dgp0aou
Files in database submission	The following files are in the database submission:<br><br>KP-Ctrl, H3K36me2, biol rep 1<br>KP-Ctrl, H3K36me2, biol rep 2<br>KP-Setd2KO, H3K36me2, biol rep 1<br>KP-Setd2KO, H3K36me2, biol rep 2<br>KP-H3K36M-Ctrl, H3K36me2, biol rep 1<br>KP-H3K36M-Ctrl, H3K36me2, biol rep 2<br>KP-H3K36M-Setd2KO, H3K36me2, biol rep 1<br>KP-H3K36M-Setd2KO, H3K36me2, biol rep 2<br>KP-Ctrl, H3K36me3, biol rep 1<br>KP-Ctrl, H3K36me3, biol rep 2<br>KP-Setd2KO, H3K36me3, biol rep 1<br>KP-Setd2KO, H3K36me3, biol rep 2<br>KP-H3K36M-Ctrl, H3K36me3, biol rep 1<br>KP-H3K36M-Ctrl, H3K36me3, biol rep 2<br>KP-H3K36M-Setd2KO, H3K36me3, biol rep 1<br>KP-H3K36M-Setd2KO, H3K36me3, biol rep 2<br>KP-Ctrl, H3K27me3, biol rep 1

KP-Ctrl, H3K27me3, biol rep 2
KP-Setd2KO, H3K27me3, biol rep 1
KP-Setd2KO, H3K27me3, biol rep 2
KP-H3K36M-Ctrl, H3K27me3, biol rep 1
KP-H3K36M-Ctrl, H3K27me3, biol rep 2
KP-H3K36M-Setd2KO, H3K27me3, biol rep 1
KP-H3K36M-Setd2KO, H3K27me3, biol rep 2
Input DNA
IgG Control

Genome browser session (e.g. UCSC)

Provide a link to an anonymized genome browser session for "Initial submission" and "Revised version" documents only, to enable peer review. Write "no longer applicable" for "Final submission" documents.

Methodology

Replicates	Each experimental condition used had n=2 biological replicates.
Sequencing depth	Each sample had ~50 million reads. 2 × 150 bp paired-end sequencing; Illumina HiSeq was performed.
Antibodies	H3K36me2 (5 µg, abcam, ab9049), H3K36me3 (5 µg, abcam, ab9050), H3K27me3 (5 µg, Cell Signaling Technologies, 9733T), and mouse IgG (1 µg, Diagenode, C15400001-15)
Peak calling parameters	The reads from these samples were first trimmed using Atria (default options, v4.0.0) and then aligned to mm39 (GRCm39) using bowtie2 (“bowtie align” using options –local –no-mixed –discordant, v2.5.1) [57]. Reads were then also aligned to the Drosophila genome dm6 (BDGP Release 6 + ISO1 MT), and the number of reads mapping to this genome was counted, and the percentage of Drosophila reads in each sample was calculated.
Data quality	In all NGS experiments, the fastq samples were subject to QC using FastQC. Adapter reads and low quality reads were trimmed from the fastq files using Atria. RNA seq samples were mapped using Salmon and the validate mappings option was used to ensure more sensitive mapping to the reference genomes. To ensure proper normalization of the RNA seq samples, the robust median of ratios method of DESeq was used to calculate normalized counts for genes. These counts were then used for GSEA enrichment analyses. To ensure robustness and reliability of peaks called in the ATAC-Seq dataset, only peaks with FDR < 0.05 were used.
Software	Bowtie2 (v2.5.1) with default parameters was used for alignment. Using DeepTools, bigWig files for the samples were then generated using the scaleFactors option while providing respective scaling factors calculated in the previous step. RepeatMasker masked repeat Sequences were downloaded from the UCSC Genome browser database. Using Deeptools, matrices were computed using the ‘computeMatrix’ command for the different repeat elements and plotted using the ‘plotHeatmap’ command.
