# Supplementary material for: Antagonistic roles of histone H3 lysine methyltransferases dictate tumor immune surveillance
Source: Nat Commun. 2026 Sep 14;17:10810. doi: 10.1038/s41467-026-76657-3 (PMC13669384; doi:10.1038/s41467-026-76657-3)
Supplement: Supplementary file 4 [file 41467_2026_76657_MOESM8_ESM.pdf]

nature portfolio

Peer Review File

Antagonistic roles of histone 3 lysine methyltransferases dictate tumor immune surveillance

Corresponding Author: Dr David Feldser

This file contains all reviewer reports in order by version, followed by all author rebuttals in order by version.

Version 0:

Reviewer comments:

Reviewer #1

(Remarks to the Author)
Gladstein et al. investigate the opposing roles of histone H3 lysine methyltransferases in regulating tumor immune surveillance, focusing on KRAS-driven lung cancer using both autochthonous and xenograft models. They report that Setd2 loss promotes tumor formation and generates an immune-cold microenvironment. The authors also use the oncohistone H3K36M—which inhibits the catalytic activity of H3K36 methyltransferases such as SETD2 and NSD2, both previously implicated in lung adenocarcinoma. Unexpectedly, although H3K36M is generally considered oncogenic, it functions as a tumor suppressor in these models, potentially by derepressing endogenous retroviral elements (ERVs) and inducing a double-stranded RNA–mediated immune response that limits tumor growth. This effect appears SETD2-dependent, as H3K36M–Setd2KO tumors are larger and show minimal immune activation.
The study provides intriguing evidence that Setd2 and Nsd2—enzymes responsible for H3K36 mono- and di-methylation—help restrict ERV expression and dsRNA-triggered immune activation, thereby supporting tumor growth. A notable finding is that Setd2 appears necessary for Nsd2's role in repressing ERVs. Surprisingly, H3K36M, which inhibits both Setd2 and Nsd2, cannot restore dsRNA production or immune activation in the absence of Setd2, suggesting a non-catalytic function for Setd2 in this process. The authors also show that Ezh2 loss, likely through reduced H3K27me3 deposition, re-establishes dsRNA generation and immune activation, thereby suppressing tumorigenesis. For this reviewer, this interpretation conflicts with the proposed non-histone, non-catalytic role, given that H3K27me3 (EZH2) and H3K36me2/3 (NSD2/SETD2) typically occupy opposing chromatin domains and may in this context redistribute to compensate for each other.
Overall, although the conceptual framework is compelling, several issues limit the strength and generalizability of the conclusions. The functional consequences of immune recruitment, the novelty of NSD2's role relative to prior work, and the translational relevance to human tumors require clarification. The roles of Setd2 and Ezh2 remain mostly descriptive. Furthermore, many conclusions rely heavily on murine data, with insufficient discussion of how these findings translate to human disease.
Major Comments
1. Speculative and primarily descriptive claims regarding non-histone SETD2 functions
The authors propose that SETD2 acts as a master regulator through non-histone mechanisms not inhibited by H3K36M. While intriguing, this hypothesis is unsupported by experimental data. Validation using catalytically impaired SETD2, and rescue of the double H3K36M–Setd2KO model with wild-type versus catalytically inactive Setd2, would be informative. Moreover, the manuscript does not address known epigenetic crosstalk among H3K36me2/3 and H3K27me2/3. Previous studies show that H3K27 methylation spreads into regions lacking H3K36 methylation, altering transcription. Here, only IHC is presented, with no direct chromatin mapping to assess redistribution of these marks in Setd2KO, K36M, Setd2KO–K36M, or Setd2KO–K36M–Ezh2KO tumors. The KP-H3K36M-Setd2KO–Ezh2KO model could also directly test whether dsRNA induction and sensing are necessary for the observed immune-mediated tumor control.
Minor comment: The introduction inaccurately states that "SETD2 is the only enzyme known to catalyze mono-, di-, and tri-methylation states." This is true only in yeast or in vitro. In mammalian cells, SETD2 primarily catalyzes H3K36 trimethylation, occasionally from monomethylated substrates. Reference 1 and related literature are misinterpreted; this should be corrected.
2. Incomplete characterization of immune subtypes
The immune landscape is insufficiently described. The identities of infiltrating T-cell subsets (e.g., Tregs, $CD8^{+}$ cytotoxic T cells, double-negative T cells) in KH3K36M-Ctrl tumors remain unclear, as does the composition of the myeloid compartment. Langerhans-type dendritic cells are not assessed, and macrophage populations are simplified into TAM1 and

TAM2, which does not capture their known heterogeneity.
3. Pathway dependencies not established
It is unclear whether T-cell recruitment depends on module 6 pathways (e.g., Cxcl9, Jak/Stat). Modules 2, 3, 4, and 7—seemingly specific to KH3K36M–Setd2^KO^ tumors—are not discussed in relation to immune activation. Further clarification would strengthen the mechanistic interpretation.
4. Interpretation of human data
Patients with high NSD2 expression exhibit downregulation of T- and NK-cell-related pathways. It is unclear whether this reflects reduced lymphoid infiltration or altered immune-cell function. The implications of K36M-mutant or SETD2-deficient tumors for immune infiltration—particularly regarding ERV expression and dsRNA sensing—should be addressed explicitly.
5. Underexplored ATAC-seq and gene-expression data
The ATAC-seq results in Extended Data Fig. 7a–c are not sufficiently analyzed. These data could illuminate chromatin accessibility changes underlying transcriptional rewiring. Additionally, the minimal gene-expression changes observed upon Setd2 inactivation in H3WT-expressing cell lines (Fig. 3b–c), despite reduced H3K36me3, should be discussed to clarify how SETD2 influences transcription.
6. Limited insight from NSD1 versus NSD2 comparison
The comparison yields little information for NSD1, as K-Nsd1KO tumors arose in only one mouse. This may indicate that NSD1 loss impairs viability or has a distinct biological role in this system.
7. Need for direct evidence of ERV derepression in NSD2-deficient tumors
The claim that Nsd2^KO^ phenocopies H3K36M via ERV derepression and dsRNA induction requires direct demonstration. Without this, the mechanistic model remains speculative.
Overall Assessment
This study addresses an important intersection between chromatin regulation and tumor immune surveillance using sophisticated genetic models and provides intriguing evidence connecting histone methylation to immune activation in KRAS-driven lung cancer. However, several mechanistic gaps, overinterpretations, and limited exploration of human relevance weaken the manuscript's impact. Addressing the points above would substantially strengthen the work and enhance its translational significance.

Reviewer #2

(Remarks to the Author)
I co-reviewed this manuscript with one of the reviewers who provided the listed reports. This is part of the Nature Communications initiative to facilitate training in peer review and to provide appropriate recognition for Early Career Researchers who co-review manuscripts.

Reviewer #3

(Remarks to the Author)
In this manuscript, Gladstein et al report their findings on the role of H3K36 methylation regulators in the pathogenesis of lung adenocarcinoma. They primarily utilized a mouse model that expresses the oncohistone H3K36M in conjunction with KRAS driven tumors and crossed into SETD2 null background to generate a cohort of mice and tumors with single, double and triple genetic lesions. They tracked and quantified tumor development in the lungs, performed RNA-seq and spatial transcriptomics to profile the repertoire of immune infiltration in these tumors. Their subsequent epigenomic investigations revealed key impact of H3K36 methylation on endogenous retroviral elements. Based on their observations, the authored have produced a working model where by H3K36 and K27 methylation as regulated by NSD2, SETD2 and EZH2 play a prominent role in dsRNA expression from endogenous retroviral elements leading to type I interferon production that triggers inflammation and tumor killing.
Overall, the report is supported by high quality data, rigorous mouse modeling and profiling experiments. There are potential therapeutic implications that can be derived from this work including use of NSD2 and EZH2 inhibitors to potentiate anti-tumor immunity which makes it of interest to a broader audience.
The manuscript can be strengthened by addressing the following weaknesses.
1) The use of H3K36M oncohistone expressing mouse models to understand role of H3K36 methylation is mechanistically elegant. However, the major caveat is this mutation does not occur in lung adenocarcinoma. Thus, the relevance of this model to study human lung adenocarcinoma is minimal. Hence, in order to confirm and elevate the human disease relevance of the study, additional approaches to manipulate, modulate and measure anti-tumor immune relevance of H3K36me2/me3 need to be done. This includes: use of pharmacologic approaches in vivo to inhibit NSD2 and/or EZH2 in their autochtonous/orthotopic mouse models to document whether inhibition of H3K36me2 and H3K27me3 impacts expression of endogenous retroviral elements as measured for example by dsRNA, alter expression of type I interferons, affect infiltration of tumors with immune cells and ultimately its impact on tumor burden.
2) The authors state that introduction of the oncohistone H3 K36M substantially reduced the number of tumors observed and the size of each tumor in Fig 1bc. However, they are NOT statistically significant as seen in Fig 1C and Ext Data Fig 1a. This is an important point as the authors conclude that "… H3K36M suppresses tumor formation and growth in a Setd2-dependent manner…" This might require additional sample size to get appropriate statistical power.
3) This is further noted in Ext Data Fig. 5D which shows that H3K36M appears to have the same impact as Control on tumor burden. Authors need to show pairwise statistical tests between all the 4 cohorts in this figure. Looking at the data as presented, it is obvious that the Setd2 ko is the dominant genetic lesion with further introduction of H3K36M having a modest impact, at best, on tumor burden.
4) Fig 1d shows modest expression of H3K36M in the control group while it is highly expressed in the Setd2ko. Importantly,

this is seen again in Fig 3b (orthotopic model experiment, whereby K36M expression is higher in the Setd2 KO background. What is the cause of this? This raises the question of whether the level of expression of K36M is modulated by Setd2.
5) The authors report in Ext Data Fig 7d that "..Setd2 inactivation had a more subtle effect…" while describing the ChIP-seq results. This is quite in contrast to the H3K36me3 IHC images shown in Fig 1D that showed substantial abolishment of total H3K36Me3 histone mark in the K Setd2 KO tumors. This needs to be investigated further and source of discrepancy resolved as it is a crucial piece of information (with potential differences arising from orthotopic vs autochtonous model difference, IHC vs ChIP-seq assay, antibody difference etc..). As further supporting data, authors need to investigate this in human lung adenocarcinoma cell lines and show difference between SETD2 WT vs mutant cell lines.

Reviewer #4

(Remarks to the Author)
Major issues:

1.The manuscript presents a compelling "enzymatic triad" model (NSD2, SETD2, and EZH2) that dictates tumor immune surveillance. While the scientific foundation is robust, the current presentation, particularly in the Abstract, is linguistically dense and could more effectively highlight the study's paradoxical nature to engage a broader readership. I have the following specific suggestions:

The sentence in the Abstract ("Specifically, loss of ... leads to derepression of ... that activates ... that ... that...") is structurally over-complex due to nested "that" clauses. Coupled with high-density epigenetic jargon, this may hinder the immediate grasp of the causal chain. I suggest decoupling these steps to explicitly highlight the viral mimicry mechanism; for example: "Specifically, loss of H3K36me2/3 (via H3K36M expression or NSD2 inactivation) derepresses endogenous retroviral elements (ERVs). The resulting transcripts form dsRNA, triggering an antiviral-like immune response that potently eradicates KRAS-driven tumors."
The term "ERV" is used later in the abstract (and throughout the text), but its full name, "endogenous retroviral elements," is mentioned without being linked to the abbreviation upon its first occurrence. For the sake of clarity, please introduce the abbreviation immediately following the full term: "endogenous retroviral elements (ERVs)."
The most striking feature of this work is the biological paradox it resolves. However, the Abstract and the end of the Introduction could more sharply delineate the "Knowns" vs. the "Paradox." I suggest the authors restructure these sections to follow a clearer "Known Background -->Paradox --> Mechanistic Solution" template if I get the core idea correctly, e.g.
- The Known: it's established that H3K36M inhibits the function of SETD2; and SETD2 loss is a potent driver of KRAS-driven LUAD;
- The Paradox: Logic dictates that H3K36M should be oncogenic in this context; however the authors found it tumor-suppressive; and what's more, this suppression requires SETD2, the enzyme that H3K36M blocks.
- The Answer: H3K36M inhibits H3K36 methylation; SETD2 prevents EZH2 from re-silencing ERVs, hence maintaining immune-mediated tumor killing.

2.The Discussion provides a fascinating mechanistic insight into why H3K36M, traditionally viewed as an oncogenic, acts as a tumor suppressor in KRAS-driven lung adenocarcinoma. To further broaden the impact of this study, I suggest the authors consider adding a brief elaboration on the tissue-specific context of this paradox.

While the authors acknowledge the 'bona fide oncogenic potential' of H3K36M in many other tumor types, the finding that it triggers potent immune-mediated eradication in the lung is particularly striking. Adding a sentence or two to discuss whether this reversal is driven by the unique immune surveillance landscape of the lung—compared to the more 'immune-cold' environments like bone or cartilage where H3K36M is oncogenic, would provide valuable context.

3. The current manuscript lacks sufficient technical detail regarding the derivation and calculation of the "Hotspot Modules" and their respective "Module Scores." Please clarify the definition and composition of the modules and the module scoring algorithm in the method parts.

4. The manuscript states that Xenium spatial transcriptomics was performed across four experimental groups (K-Ctrl, K-Setd2KO, KH3K36M-Ctrl, KH3K36M-Setd2KO). Please clarify, for each group, how many independent mice were analyzed (and how many lung lobes/sections per mouse), and how these replicates were incorporated into the statistical analyses (e.g., mouse-level aggregation vs. treating tumors/cells as independent observations). And are these replicates sufficient statistically?

5. The Data Availability statement is unclear regarding deposition of newly generated datasets (e.g., Xenium spatial transcriptomics, and other sequencing-based assays). Please explain why these datasets are not publicly available, and provide accession numbers/links if they have been deposited (e.g., GEO/SRA/ENA or an equivalent repository), or a timeline and mechanism for access if deposition is pending.

Minor issues:

6. Do spatial plot panels and H&E panels in Fig2a share the same scale? The y-axes of different panels in Extended Data

Fig. 7a. should be aligned. And the outlier values could be clipped to better present the squeeze genes in the volcano plots.

7. In Extended Data Fig. 7b, the enriched gene sets are too blurred. Please use high-resolution figures instead.

8. It's not necessary to arrange genes of 7 modules in tabular format in Supplemental Table 1. Seven lists of genes would make this part more readable.

Version 1:

Reviewer comments:

Reviewer #1

(Remarks to the Author)
the authors performed an extensive revision of the manuscript strengthening and clarifying several points.
I have no additional comments

Reviewer #2

(Remarks to the Author)
I co-reviewed this manuscript with one of the reviewers who provided the listed reports. This is part of the Nature Communications initiative to facilitate training in peer review and to provide appropriate recognition for Early Career Researchers who co-review manuscripts.

Reviewer #3

(Remarks to the Author)
The authors have substantially revised & improved the manuscript. Most major concerns are now addressed.
However, there are outstanding issues that need to be addressed.
1) I could not locate Extended Data Fig 10f-h that is cited as a supporting evidence regarding nsd2 knockout and ERV and hence can not adequately assess the response to my question #1.
Also, Fig 5h is missing (cited in response to question #1).
2) in Ext fig 12d dsRNA image is very dim and can not be assessed. the overlay shows simply the loss of the strong H3K27me3 signal. Better images need to provided.
3) Authors need to show the comparison and non-significant p-value of 0.1493 in ext. fig 5d (from response to question #3)

Reviewer #4

(Remarks to the Author)
The authors have answered my questions. I have no additional comments towards this manuscript.

Open Access This Peer Review File is licensed under a Creative Commons Attribution 4.0 International License, which

permits use, sharing, adaptation, distribution and reproduction in any medium or format, as long as you give appropriate credit to the original author(s) and the source, provide a link to the Creative Commons license, and indicate if changes were made.
In cases where reviewers are anonymous, credit should be given to 'Anonymous Referee' and the source.
The images or other third party material in this Peer Review File are included in the article's Creative Commons license, unless indicated otherwise in a credit line to the material. If material is not included in the article's Creative Commons license and your intended use is not permitted by statutory regulation or exceeds the permitted use, you will need to obtain permission directly from the copyright holder.
To view a copy of this license, visit https://creativecommons.org/licenses/by/4.0/

ALL REVIEWERS: We thank the reviewers for their careful evaluation of our manuscript and for their insightful and constructive suggestions. We appreciate their recognition of the conceptual significance of our work linking histone methyltransferase activity to tumor immune surveillance in KRAS-driven lung adenocarcinoma. The reviewers' comments helped us substantially improve the clarity, rigor, and translational relevance of the study.

In the revised manuscript, we have strengthened several key aspects of the work. First, we clarified the mechanistic interpretation of SETD2 function and added new rescue experiments demonstrating that SETD2 catalytic activity is required to restore H3K36me3 and dsRNA accumulation in *H3*^{K36M}-expressing, *Setd2*-deficient tumor cells. Second, we expanded the functional evidence linking dsRNA sensing to tumor immune surveillance by genetically inactivating the dsRNA sensors MDA5/*Ifih1* and RIG-I/*RigI* in autochthonous tumors. Third, we added direct evidence that *Nsd2* loss and pharmacologic NSD2 inhibition promote ERV/LINE/SINE expression and dsRNA accumulation, strengthening the connection between H3K36me2 loss, viral mimicry, and immune activation. Fourth, we expanded the human relevance of the study through additional analyses of human lung adenocarcinoma datasets and pharmacologic experiments in human lung cancer cell lines, including combinatorial perturbation of NSD2, SETD2, and EZH2 activity. Finally, we revised the text, figure presentation, Methods, and Data Availability information to improve clarity, transparency, and readability.

Below, we provide a detailed point-by-point response to each comment. Reviewer comments are reproduced below, followed by our responses in blue text. We have also highlighted the changes in the figures and manuscript text in blue for ease of review.

REVIEWER #1

"Gladstein et al. investigate the opposing roles of histone H3 lysine methyltransferases in regulating tumor immune surveillance, focusing on KRAS-driven lung cancer using both autochthonous and xenograft models. They report that Setd2 loss promotes tumor formation and generates an immune-cold microenvironment. The authors also use the oncohistone H3K36M—which inhibits the catalytic activity of H3K36 methyltransferases such as SETD2 and NSD2, both previously implicated in lung adenocarcinoma. Unexpectedly, although H3K36M is generally considered oncogenic, it functions as a tumor suppressor in these models, potentially by derepressing endogenous retroviral elements (ERVs) and inducing a double-stranded RNA–mediated immune response that limits tumor growth. This effect appears SETD2-dependent, as H3K36M–Setd2KO tumors are larger and show minimal immune activation.
The study provides intriguing evidence that Setd2 and Nsd2—enzymes responsible for H3K36 mono- and di-methylation—help restrict ERV expression and dsRNA-triggered immune activation, thereby supporting tumor growth. A notable finding is that Setd2 appears necessary for Nsd2's role in repressing ERVs. Surprisingly, H3K36M, which inhibits both Setd2 and Nsd2, cannot restore dsRNA production or immune activation in the absence of Setd2, suggesting a non-catalytic function for Setd2 in this process. The authors also show that Ezh2 loss, likely through reduced H3K27me3 deposition, re-establishes dsRNA generation and immune activation, thereby suppressing tumorigenesis. For this reviewer, this interpretation conflicts with the proposed non-histone, non-catalytic role, given that H3K27me3 (EZH2) and H3K36me2/3 (NSD2/SETD2) typically occupy opposing chromatin domains and may in this context redistribute to compensate for each other.
Overall, although the conceptual framework is compelling, several issues limit the strength and generalizability of the conclusions. The functional consequences of immune recruitment, the

novelty of NSD2's role relative to prior work, and the translational relevance to human tumors require clarification. The roles of Setd2 and Ezh2 remain mostly descriptive. Furthermore, many conclusions rely heavily on murine data, with insufficient discussion of how these findings translate to human disease."

We thank the reviewer for their thoughtful evaluation of our work and for recognizing the conceptual importance of the study. We also appreciate the reviewer's careful consideration of the mechanistic interpretation and translational implications of our findings. In response, we have added new experimental data, expanded the analysis of existing datasets, and revised the text to clarify the scope of our conclusions.

We would first like to clarify our interpretation of SETD2 function in this context. The reviewer noted that our model might imply a non-catalytic role for SETD2 in ERV derepression and dsRNA induction. Our intention was not to assert a definitive non-catalytic mechanism, but rather to highlight an unexpected observation: $H3^{K36M}$ suppresses H3K36 methylation, yet fails to induce dsRNA accumulation and immune activation in the absence of SETD2. We agree that this raised an important mechanistic question. To address it directly, we added new rescue experiments in *KP-$H3^{K36M}$-$Setd2^{KO}$* cells using wild-type human truncated SETD2 and a catalytically inactive SETD2 mutant. Wild-type SETD2 restored H3K36me3 and dsRNA accumulation, whereas catalytically inactive SETD2 did not (**Extended Data Fig. 9a-d**). These data demonstrate that SETD2 catalytic activity is required for this phenotype. However, we have revised the manuscript to avoid over-interpreting the identity of the relevant SETD2 substrate. In particular, we do not conclude that SETD2 acts through a non-histone mechanism. Rather, our data support a model in which SETD2 catalytic activity restrains EZH2-dependent repression of ERV-derived transcripts in the context of H3K36me2 loss. We have also added new data further supporting the role of EZH2 in regulating dsRNA expression in this setting (**Fig. 6a-e and Extended Data Fig. 12c-e**).

The reviewer also raised the important question of whether the immune response observed in *$H3^{K36M}$*-expressing tumors is functionally responsible for tumor suppression. We agree that causality is central to the interpretation of our study. The original manuscript already included several complementary lines of evidence supporting this conclusion: *$H3^{K36M}$*-expressing cells did not show a significant growth defect *in vitro*, were poorly tolerated in immune-competent mice, and formed tumors efficiently in severely immunodeficient NCG mice (**Extended Data Fig. 5a-d and Extended Data Fig. 6a,b**). We have now strengthened this point substantially by adding genetic validation of the dsRNA-sensing pathway in autochthonous tumors. Specifically, inactivation of either *Ifih1*/MDA5 or *RigI* restored growth of *$H3^{K36M}$*-expressing tumors and markedly reduced immune infiltration, despite persistent dsRNA expression in the tumor cells (**Fig. 4h,i and Extended Data Fig. 8c-f**). These new data provide direct functional evidence that tumor-cell-intrinsic dsRNA sensing is required for immune recognition and suppression of *$H3^{K36M}$*-expressing tumors.

Finally, we agree with the reviewer that the translational relevance of the work should be articulated more clearly. We have therefore expanded both the experimental and computational analysis of human lung adenocarcinoma. In the revised manuscript, we include additional analyses of human LUAD datasets assessing the relationship between NSD2 expression, SETD2 loss-of-function alterations, and immune infiltration (**Extended Data Fig. 11a-c**). We also added pharmacologic experiments in human lung cancer cell lines showing that NSD2 inhibition induces ERV/LINE/SINE expression and dsRNA accumulation, that SETD2 inhibition attenuates this response, and that EZH2 inhibition

can restore dsRNA accumulation in this setting (**Fig. 5a-e and Extended Data Fig. 12a-e**). Together, these additions strengthen the translational framework of the study and support the therapeutic relevance of the NSD2–SETD2–EZH2 axis in human lung cancer.

Major Comments

Q1.1a: "Speculative and primarily descriptive claims regarding non-histone SETD2 functions. The authors propose that SETD2 acts as a master regulator through non-histone mechanisms not inhibited by H3K36M. While intriguing, this hypothesis is unsupported by experimental data. Validation using catalytically impaired SETD2, and rescue of the double H3K36M–Setd2KO model with wild-type versus catalytically inactive Setd2, would be informative. Moreover, the manuscript does not address known epigenetic crosstalk among H3K36me2/3 and H3K27me2/3. Previous studies show that H3K27 methylation spreads into regions lacking H3K36 methylation, altering transcription. Here, only IHC is presented, with no direct chromatin mapping to assess redistribution of these marks in Setd2KO, K36M, Setd2KO–K36M, or Setd2KO–K36M–Ezh2KO tumors."

A1.1a: We appreciate the reviewer's thoughtful comments regarding SETD2 function and the potential relationship between H3K36 and H3K27 methylation in our model. We agree that distinguishing catalytic, non-catalytic, histone-directed, and non-histone-directed functions of SETD2 is important. Our original intent was not to conclude that SETD2 acts through a non-catalytic mechanism. Rather, our data revealed an unexpected observation: although *H3*^{K36M} suppresses H3K36me3 deposition, *H3*^{K36M}-induced dsRNA accumulation and immune activation are lost when *Setd2* is inactivated. This raised the possibility that residual SETD2 activity, or SETD2-dependent regulation of another chromatin pathway, is required for dsRNA induction in the context of H3K36me2 loss.

Our original data already supported a central role for H3K36/H3K27 methylation crosstalk in this process. *H3*^{K36M} expression reduced H3K36me3 while promoting dsRNA accumulation (**Fig. 3b,c and Fig. 4a-d**). In contrast, *KP-H3*^{K36M}*-Setd2*^{KO} cells showed increased H3K27me3 by immunoblotting and mass spectrometry (**Fig. 3b,c**). Moreover, our ChIP-seq analyses demonstrated a marked loss of H3K27me3 at LINE, SINE, and LTR retroelement sequences in *KP-H3*^{K36M}*-Ctrl* cells, whereas H3K27me3 was restored at these elements in *KP-H3*^{K36M}*-Setd2*^{KO} cells (**Fig. 3d-f**). Finally, genetic inactivation of EZH2 in *KP-H3*^{K36M}*-Setd2*^{KO} cells restored dsRNA accumulation (**Fig. 4e**). Together, these data support a model in which SETD2 restrains EZH2-dependent repression of ERV-derived transcripts in the setting of H3K36me2 loss, and they directly address the known antagonism between H3K36me2/3 and H3K27me3.

We also appreciate the reviewer's suggestion that chromatin mapping in tumors would be informative. In the current study, we performed ChIP-seq in controlled tumor-cell systems rather than whole tumors because the tumor genotypes analyzed *in vivo* have dramatically different levels of immune infiltration, tumor-cell abundance, tumor size, and stromal composition. These differences are central to the biology of the study, but they would also substantially confound bulk chromatin profiling of whole tumor tissue. The KP cell-line system allowed us to compare matched tumor-cell genotypes under conditions where tumor-cell-intrinsic chromatin changes could be interpreted

without differences in immune and stromal cell content. Importantly, these ChIP-seq experiments directly mapped H3K27me3, H3K36me2, and H3K36me3 at LINE, SINE, and LTR retroelement sequences across the relevant *$H3^{WT}$*, *$Setd2^{KO}$*, *$H3^{K36M}$*, and *$H3^{K36M}$-$Setd2^{KO}$* tumor-cell contexts (**Fig. 3d-f**). Thus, while we agree that *in vivo* chromatin mapping is an interesting future direction, the existing ChIP-seq data provide direct evidence for redistribution of these marks at retroelements in the tumor-cell compartment most relevant to dsRNA production.

To directly test whether SETD2 catalytic activity is required for dsRNA accumulation, we performed new rescue experiments in *KP-$H3^{K36M}$-$Setd2^{KO}$* cells. In these cells, endogenous mouse *Setd2* was inactivated using an sgRNA targeting an N-terminal coding exon. We then expressed an N-terminally truncated human SETD2 construct, htSETD2WT amino acids 567-2564, which lacks the mouse sgRNA target sequence but retains methyltransferase activity and is commonly used for SETD2 functional studies. Because full-length SETD2 is exceptionally large, this truncated construct provides a technically tractable approach to test SETD2 catalytic function. In parallel, we expressed a catalytically inactive htSETD2 mutant containing R1625G and C1631A substitutions in the SET domain. Expression of htSETD2WT restored H3K36me3 and dsRNA accumulation, whereas expression of the catalytically inactive htSETD2 mutant did not (**Extended Data Fig. 9a-c**). These data demonstrate that SETD2 catalytic activity is required for dsRNA induction in *$H3^{K36M}$*-expressing cells. Importantly, we have revised the manuscript to avoid concluding that the relevant substrate is definitively histone or non-histone. Instead, we now state more precisely that SETD2 catalytic activity is required and that our data are consistent with a model in which SETD2 limits EZH2-dependent repression of ERV-derived transcripts.

We have also expanded these analyses using pharmacologic perturbation of NSD2, SETD2, and EZH2 in human lung cancer cell lines. In this system, NSD2 inhibition induced dsRNA accumulation over a six-day time course. Combined NSD2 and SETD2 inhibition markedly attenuated dsRNA accumulation, paralleling the genetic interaction observed in mouse KP cells. However, addition of an EZH2 inhibitor to the NSD2 and SETD2 inhibitor combination restored dsRNA accumulation, whereas EZH2 inhibition alone did not induce dsRNA (**Fig. 5a-e; Extended Data Fig. 12c-e**). Thus, this human cancer cell system recapitulates the central genetic findings from the mouse models: dsRNA induction following loss of H3K36me2 requires SETD2 catalytic activity and is opposed by EZH2-dependent repression.

In response to the reviewer's concern, we have revised the Results and Discussion to more explicitly describe the known crosstalk between H3K36 and H3K27 methylation and to clarify how our data support an EZH2-dependent mechanism of ERV repression in the absence of SETD2.

Q1.1b: "The KP-H3K36M-Setd2KO–Ezh2KO model could also directly test whether dsRNA induction and sensing are necessary for the observed immune-mediated tumor control."

A1.1b: We agree with the reviewer that the *KP-$H3^{K36M}$-$Setd2^{KO}$-$EZH2^{KO}$* model would be an interesting system in which to further dissect how EZH2-dependent ERV repression interfaces with dsRNA sensing and immune control. However, we respectfully note that the central issue raised by the reviewer—whether dsRNA sensing is required

for immune-mediated tumor control—is directly addressed by both our original and newly added data.

In the original submission, we used a pooled CRISPR-based *in vivo* screening approach to identify genes whose loss allowed *$H3^{K36M}$*-expressing tumor cells to evade immune-mediated elimination. This screen identified the dsRNA sensors MDA5/*Ifih1* and RIG-I/*RigI* as the most enriched genes, indicating that dsRNA sensing is required for immune elimination of *$H3^{K36M}$*-expressing cells (**Fig. 4f,g**). We have now strengthened this conclusion with new genetic experiments in the KRAS-driven autochthonous model. Specifically, we inactivated *Ifih1*/MDA5 or *RigI* in *$H3^{K36M}$*-expressing tumors and observed significantly increased tumor growth compared with *$H3^{K36M}$*-expressing tumors with intact dsRNA sensing (**Fig. 4h,i and Extended Data Fig. 8c,d**). Importantly, *$KH3^{K36M}$-$Ifih1^{KO}$* and *$KH3^{K36M}$-$RigI^{KO}$* tumors retained high levels of dsRNA but showed markedly reduced immune infiltration relative to *$H3^{K36M}$*-expressing tumors with intact sensors (**Fig. 4i and Extended Data Fig. 8e,f**). These data demonstrate that dsRNA accumulation alone is not sufficient for tumor immune control; tumor-cell-intrinsic sensing through MDA5 and RIG-I is required to translate dsRNA accumulation into immune recognition.

We also note that our new pharmacologic experiments in human lung cancer cells further support the context-dependent role of EZH2 in this pathway. EZH2 inhibition alone did not induce dsRNA accumulation. However, when NSD2 and SETD2 were inhibited together, addition of EZH2 inhibition restored dsRNA accumulation over a six-day time course. (**Fig. 5e-h**) Notably, EZH2 inhibition alone did not cause increased dsRNA accumulation, indicating that EZH2 inhibition does not act as a nonspecific inducer of dsRNA (**Extended Data Fig. 12c,d**). Rather, these data support a model in which EZH2-dependent repression becomes functionally relevant when H3K36me2 is reduced and SETD2 activity is compromised.

Thus, while we agree that *KP-$H3^{K36M}$-$Setd2^{KO}$-$EZH2^{KO}$* tumors could provide an additional context for studying EZH2-dependent ERV regulation, the requirement for dsRNA sensing in immune-mediated tumor suppression is now directly demonstrated genetically in autochthonous *$H3^{K36M}$*-expressing tumors. We have revised the manuscript to emphasize this point and to clarify the relationship between EZH2-dependent dsRNA regulation and MDA5/RIG-I-dependent immune activation.

MC1.1: "Minor comment: The introduction inaccurately states that "SETD2 is the only enzyme known to catalyze mono-, di-, and tri-methylation states." This is true only in yeast or in vitro. In mammalian cells, SETD2 primarily catalyzes H3K36 trimethylation, occasionally from monomethylated substrates. Reference 1 and related literature are misinterpreted; this should be corrected."

A1.1: We thank the reviewer for raising this point. SETD2 has been reported to catalyze multiple H3K36 methylation states *in vitro*, but we agree that its best-established and most relevant function in mammalian cells is H3K36 trimethylation. To avoid ambiguity and keep the Introduction focused on the biology most relevant to this study, we have revised the text to state that "Multiple enzymes, such as NSD1 and NSD2, catalyze the mono- and di-methylation states of H3K36, while SETD2 is the only enzyme known to catalyze the tri-methylation state in mammalian cells."

A1.2: We agree with the reviewer that additional immune-subtype characterization would improve the manuscript. In the initial submission, we profiled immune infiltration by IHC for CD45, CD3, B220, NKp46, F4/80, and Ly6G, allowing us to quantify total leukocytes, T cells, B cells, NK cells, macrophages, and neutrophils across tumor genotypes. We also used Xenium spatial transcriptomics to characterize the spatial organization of malignant and immune cells within tumors. While this approach was powerful for identifying the dominant immune-cell classes and their spatial relationships, we agree that the targeted spatial transcriptomic panel did not provide sufficient resolution to confidently classify all immune subtypes, including specific T-cell and myeloid subsets.

To address this concern, we have added new IHC analyses for CD8, Foxp3, CD11c, and CD207 to evaluate cytotoxic T cells, regulatory T cells, and dendritic cells respectively. These new analyses showed the expected increase in CD8-positive T cells specifically in *KH3^{K36M}-Ctrl tumors*. In contrast, we did not observe major differences in Foxp3-positive regulatory T cells. We thank the reviewer for noting that our initial immune characterization did not explicitly address Langerhans-type dendritic cells. In response, we added staining for CD207/Langerin and CD11c. We found that CD207 was selectively increased in *KH3^{K36M}-Ctrl* tumors compared with all other genotypes, whereas CD11c staining was similar across groups. These results indicate that H3^{K36M} expression does not cause a broad expansion of the CD11c-positive dendritic/myeloid compartment, but is associated with selective enrichment of a CD207/Langerin-positive dendritic-cell subset or state within the SETD2-dependent inflammatory tumor microenvironment. However, the absolute abundance of CD207-positive cells was low across all genotypes, suggesting that Langerhans cells are unlikely to represent a dominant mediator of tumor control. Rather, their enrichment may reflect the broader inflammatory microenvironment associated with *H3^{K36M}*-induced tumor immune activation.Nevertheless, we have added these data and revised associated text to describe the immune infiltrate with greater specificity.(**Extended Data Fig. 2b,c**).

We have also clarified the limits of our myeloid-cell interpretation. Although macrophages, dendritic cells, and other innate myeloid cells may contribute to the inflammatory tumor microenvironment, our functional data indicate that these populations are not sufficient to mediate the tumor-eradication phenotype in the absence of lymphoid and NK-cell compartments. Specifically, orthotopic transplantation of *KP-H3^{K36M}-Ctrl* cells into NCG mice, which lack T cells, B cells, and NK cells but retain myeloid-cell populations, resulted in efficient tumor growth comparable to control cells (**Extended Data Fig. 6a,b**). Thus, while myeloid cells may contribute accessory functions, the available functional data support a model in which *H3^{K36M}*-induced tumor control requires immune components absent from NCG mice, consistent with the prominent T-cell, B-cell, plasma-cell, and NK-cell signatures observed in immune-competent settings.

We have revised the Results to include these new immune-subtype data and have modified the text to more clearly state the resolution and limitations of the spatial transcriptomic and IHC-based immune profiling.

Q1.3: “Pathway dependencies not established. It is unclear whether T-cell recruitment depends on module 6 pathways (e.g., Cxcl9, Jak/Stat). Modules 2, 3, 4, and 7—seemingly specific to

KH3K36M–Setd2^KO^ tumors—are not discussed in relation to immune activation. Further clarification would strengthen the mechanistic interpretation."

A1.3: We thank the reviewer for raising this point. Our intention was to identify tumor-cell transcriptional programs associated with *H3^{K36M}*-induced inflammation and T-cell infiltration, not to imply that individual Module 6 genes are proven causal mediators of T-cell recruitment. We agree that the original wording could have been clearer on this distinction, and we have revised the manuscript accordingly.

Module 6 was identified through Hotspot analysis as a malignant-cell gene-expression module that was enriched in *K-Ctrl* tumors, further increased in *KH3^{K36M}-Ctrl* tumors, and suppressed by *Setd2* inactivation in both contexts (**Fig. 2f,g**). This module contains multiple immune-associated genes, including complement components, cytokine/chemokine-related genes such as Cxcl9 and Il18rap, and genes associated with Jak/Stat and NF-kB pathway activity, including Stat1 and Nfkb1. Importantly, Module 6 expression correlated strongly with T-cell infiltration on a per-tumor basis (**Extended Data Fig. 4b,c**). We therefore interpret Module 6 as a spatially resolved malignant-cell inflammatory program associated with immune infiltration, rather than as a genetically validated causal pathway for T-cell recruitment.

To better establish pathway dependency, we have added new functional experiments focused on the upstream mechanism that links *H3^{K36M}*-induced dsRNA accumulation to immune activation. Specifically, inactivation of either *Ifih1*/MDA5 or *RigI* in *H3^{K36M}*-expressing autochthonous tumors restored tumor growth and markedly reduced immune infiltration despite persistent dsRNA accumulation (**Fig. 4h,i and Extended Data Fig. 8c-f**). These data demonstrate that tumor-cell-intrinsic dsRNA sensing is required for immune recognition of *H3^{K36M}*-expressing tumors. Thus, while Module 6 provides a useful spatial transcriptional signature associated with this immune response, the causal pathway dependency established in the revised manuscript is the requirement for MDA5/RIG-I-mediated dsRNA sensing.

We also agree that additional discussion of other Hotspot modules would help avoid perceived overemphasis on Module 6. Modules 2, 3, 4, and 7 were relatively enriched in *KH3^{K36M}-Setd2^{KO}* tumors and were not associated with the robust immune infiltration observed in *KH3^{K36M}-Ctrl* tumors. To assign biological function to the genes in Modules 2, 3, 4, and 7 we performed GO analysis (**Rebuttal Fig. 1a-d**). We found that Module 2 has one significantly enriched gene set related to effects of exercise on cardiac growth that is difficult to interpret or assign biological significance. Module 3 was enriched for glycolytic metabolism-related gene sets, Module 4 was enriched for lipid and proliferation-related gene sets, and Module 7 was enriched for gene sets related to ECM remodeling all of which could be related to the aggressive growth phenotype observed in the *KH3^{K36M}-Setd2^{KO}* tumors. While these data are interesting, we have chosen not to include these analyses in the manuscript for clarity as these results do not contribute to our overall conclusions related to anti-viral immune responses. However, we have revised the text to clarify that these modules likely reflect transcriptional states associated with aggressive tumor growth, altered malignant-cell state, or genotype-specific tumor biology in *KH3^{K36M}-Setd2^{KO}* tumors, rather than inflammatory immune activation. This interpretation is consistent with the observation that *KH3^{K36M}-Setd2^{KO}* tumors show increased tumor growth and lack the immune infiltration and Module 6 inflammatory signature observed in *KH3^{K36M}-Ctrl* tumors.

Q1.4: "Interpretation of human data. Patients with high NSD2 expression exhibit downregulation of T- and NK-cell-related pathways. It is unclear whether this reflects reduced lymphoid infiltration or altered immune-cell function. The implications of K36M-mutant or SETD2-deficient tumors for immune infiltration—particularly regarding ERV expression and dsRNA sensing—should be addressed explicitly."

A1.4: We appreciate this comment and agree that immune pathway downregulation in human tumors requires careful interpretation. In the original submission, our human analysis showed that tumors with high *NSD2* expression were enriched for reduced T- and NK-cell-associated gene-expression programs. As the reviewer correctly notes, this analysis alone could not distinguish whether these signatures reflected reduced lymphoid infiltration, altered immune-cell function, or both.

To address this issue more directly, we performed CIBERSORT analysis on human lung adenocarcinoma datasets to estimate immune-cell composition in relation to *NSD2* expression and SETD2 genotype. These new analyses showed that tumors with low *NSD2* expression had increased immune-cell infiltration, whereas tumors with *SETD2* loss-of-function alterations showed reduced overall immune infiltration (**Extended Data Fig. 11a**). These findings are consistent with our mouse models, in which *Nsd2* inactivation promotes dsRNA accumulation and immune infiltration, while *Setd2* inactivation suppresses the inflammatory immune response.

We have also revised the manuscript to more explicitly discuss the implications of these findings for human lung adenocarcinoma. Because $H3^{K36M}$ mutations are not a recurrent feature of lung adenocarcinoma, we now emphasize $H3^{K36M}$ as a mechanistic tool that revealed a therapeutically relevant NSD2/H3K36me2 axis rather than as a common genetic event in this disease. In contrast, *NSD2* expression and *SETD2* loss-of-function alterations are directly relevant to human lung cancer biology. Our revised human-cell experiments further support this translational framework: pharmacologic NSD2 inhibition induced LINE/SINE expression and dsRNA accumulation, SETD2 inhibition attenuated this response, and combined EZH2 inhibition restored dsRNA accumulation in the NSD2i/SETD2i setting (**Fig. 5e-h and Extended Data Fig. 12a-d**). Together, these data support a model in which NSD2 inhibition may promote viral-mimicry-like immune activation in SETD2-competent tumors, while SETD2 deficiency may limit this response through EZH2-dependent repression of ERV-derived transcripts.

To further investigate the effect of NSD2 inhibition in human cancer, we treated human lung adenocarcinoma cell lines with an Nsd2 inhibitor and performed RT-qPCR for LINE/SINE element expression (**Extended Data Fig. 12b**). In agreement with our mouse data, we found that NSD2 inhibition increased the expression of ERVs. Together these new data suggest that the ERV de-repression mechanism that we have described in detail in mice can also be applied to patient tumors.

Q1.5: Underexplored ATAC-seq and gene-expression data. The ATAC-seq results in Extended Data Fig. 7a–c are not sufficiently analyzed. These data could illuminate chromatin accessibility changes underlying transcriptional rewiring. Additionally, the minimal gene-expression changes observed upon Setd2 inactivation in H3WT-expressing cell lines (Fig. 3b–c), despite reduced H3K36me3, should be discussed to clarify how SETD2 influences transcription.

A1.5: We thank the reviewer for raising this point. We agree that the RNA-seq and ATAC-seq data provide important context for interpreting how SETD2 and $H3^{K36M}$ influence transcriptional states. In the revised manuscript, we have expanded the discussion of these data and clarified their interpretation.

Previous work from our laboratory and others identified substantial transcriptional changes following *Setd2* inactivation in KRAS-driven lung adenocarcinoma *in vivo* [1, 2]. In addition, Xie et al. performed ATAC-seq on Setd2-deficient tumors and reported chromatin accessibility changes that correlated with gene-expression changes [2]. In contrast, in the present study, RNA-seq and ATAC-seq performed in matched KP cell-line models showed relatively few gene-expression or chromatin-accessibility changes following *Setd2* inactivation in $H3^{WT}$-expressing cells, despite reduced H3K36me3 (**Fig. 3b,c and Extended Data Fig. 7a-c**). We suspect this reflects an important difference between the *in vivo* and *in vitro* contexts of *Setd2* loss. Consistent with this interpretation, *Setd2* inactivation has a dramatic effect on tumor growth *in vivo*, whereas matched *Setd2*-deficient cells do not show a major growth advantage *in vitro* (**Fig. 1b,c and Extended Data Fig. 5a**). These observations suggest that many consequences of *Setd2* inactivation are context-dependent and may require tumor microenvironmental cues, *in vivo* signaling, or selective pressures that are not fully recapitulated in cell culture.

At the same time, the limited ATAC-seq changes are informative for the central mechanism of this study. *$H3^{K36M}$* expression induced inflammatory gene-expression programs, including STAT signaling and complement-related pathways, while producing relatively limited changes in chromatin accessibility (**Extended Data Fig. 7a-c**). Thus, the *$H3^{K36M}$*-induced inflammatory state does not appear to arise from broad accessibility remodeling. Instead, these data support a more specific mechanism in which altered chromatin methylation at repetitive elements promotes ERV-derived transcript expression, dsRNA accumulation, and downstream immune activation. Importantly, this SETD2-dependent dsRNA phenotype is observed both *in vitro* and *in vivo* (**Fig. 4a-c**), indicating that the key viral-mimicry phenotype is robust across systems even though the broader transcriptional consequences of Setd2 loss differ between cell culture and tumors.

We have revised the Results and Discussion to better highlight this interpretation and to clarify why the minimal transcriptional and ATAC-seq effects of *Setd2* loss in *$H3^{WT}$*-expressing cell lines do not conflict with the strong *in vivo* tumor-suppressive and immune-regulatory functions of SETD2.

Q1.6: Limited insight from NSD1 versus NSD2 comparison. The comparison yields little information for NSD1, as K-Nsd1KO tumors arose in only one mouse. This may indicate that NSD1 loss impairs viability or has a distinct biological role in this system.

A1.6: We agree with the reviewer that the NSD1 arm provides limited mechanistic insight compared with the NSD2 experiments. In the Kras-driven autochthonous model, *$K\text{-}Nsd1^{KO}$* tumors were exceptionally rare, which may indicate that *Nsd1* inactivation is poorly tolerated during KRAS-driven lung tumor initiation or that NSD1 has a distinct biological role in this setting. Moreover, the few tumors that arose retained substantial H3K36me2 staining, suggesting incomplete CRISPR-mediated *Nsd1* inactivation or selection for cells that escaped effective *Nsd1* loss (**Rebuttal Fig. 2**).

We have therefore revised the text to avoid over-interpreting the NSD1 result. The purpose of the NSD1 experiment was to compare candidate H3K36 dimethyltransferases, but the central mechanistic and translational conclusions of the manuscript are based on the NSD2 data. In contrast to the limited recovery of *K-Nsd1*^{KO} tumors, *Nsd2* inactivation reproducibly reduced H3K36me2, induced dsRNA accumulation, promoted immune infiltration, and suppressed KRAS-driven tumor growth z**Fig. 5b-d; Extended Data Fig. 10b-d**). Thus, while the NSD1 result suggests that NSD1 loss may be poorly tolerated in this model, the data identify NSD2 as the more experimentally tractable and therapeutically relevant H3K36 dimethyltransferase controlling dsRNA-mediated immune surveillance in KRAS-driven lung adenocarcinoma.

Q1.7: Need for direct evidence of ERV derepression in NSD2-deficient tumors. The claim that Nsd2^KO^ phenocopies H3K36M via ERV derepression and dsRNA induction requires direct demonstration. Without this, the mechanistic model remains speculative.

A1.7: We appreciate this suggestion and agree that direct evidence of repeat-element derepression strengthens the mechanistic link between Nsd2 inactivation and H3K36M expression. In the revised manuscript, we now directly assess ERV/repetitive element expression in *Nsd2*-deficient KP cells by RT-qPCR. These experiments show that *Nsd2* inactivation increases ERV/LINE/SINE transcript expression, in parallel with dsRNA accumulation and IFN-β production (**Extended Data Fig. 10e-h**).

We have also extended this analysis to human lung cancer cell lines treated with the NSD2 inhibitor KTX-1001. Pharmacologic NSD2 inhibition increased LINE and SINE expression and induced robust cytoplasmic dsRNA accumulation (**Extended Data Fig. 12a,b**). Thus, the revised manuscript now provides direct evidence in both mouse genetic and human pharmacologic systems that NSD2 loss or inhibition promotes repetitive-element derepression and dsRNA accumulation.

Together with the *in vivo* finding that *Nsd2* inactivation reduces H3K36me2, induces dsRNA, promotes immune infiltration, and suppresses KRAS-driven tumor growth (**Fig. 5b-d and Extended Data Fig. 10b-d**), these new data strongly support the conclusion that *Nsd2* inactivation phenocopies *H3*^{K36M} by derepressing repetitive-element transcripts that give rise to dsRNA-mediated immune activation.

REVIEWER 2

“I co-reviewed this manuscript with one of the reviewers who provided the listed reports. This is part of the Nature Communications initiative to facilitate training in peer review and to provide appropriate recognition for Early Career Researchers who co-review manuscripts.”

Thank you to the Reviewer for their contribution.

REVIEWER 3

“In this manuscript, Gladstein et al report their findings on the role of H3K36 methylation regulators in the pathogenesis of lung adenocarcinoma. They primarily utilized a mouse model that expresses the oncohistone H3K36M in conjunction with KRAS driven tumors and crossed into SETD2 null background to generate a cohort of mice and tumors with single, double and triple genetic lesions. They tracked and quantified tumor development in the lungs, performed

RNA-seq and spatial transcriptomics to profile the repertoire of immune infiltration in these tumors. Their subsequent epigenomic investigations revealed key impact of H3K36 methylation on endogenous retroviral elements. Based on their observations, the authored have produced a working model where by H3K36 and K27 methylation as regulated by NSD2, SETD2 and EZH2 play a prominent role in dsRNA expression from endogenous retroviral elements leading to type I interferon production that triggers inflammation and tumor killing.

Overall, the report is supported by high quality data, rigorous mouse modeling and profiling experiments. There are potential therapeutic implications that can be derived from this work including use of NSD2 and EZH2 inhibitors to potentiate anti-tumor immunity which makes it of interest to a broader audience."

We thank the Reviewer for their positive assessment of the study and for recognizing the rigor of the genetic models, profiling approaches, and epigenomic analyses. We also appreciate their recognition of the potential therapeutic implications of our findings. In the revised manuscript, we have added new data and analyses that further strengthen the mechanistic and translational framework linking NSD2, SETD2, and EZH2 activity to ERV-derived dsRNA expression and tumor immune surveillance.

Q3.1: The use of H3K36M oncohistone expressing mouse models to understand role of H3K36 methylation is mechanistically elegant. However, the major caveat is this mutation does not occur in lung adenocarcinoma. Thus, the relevance of this model to study human lung adenocarcinoma is minimal. Hence, in order to confirm and elevate the human disease relevance of the study, additional approaches to manipulate, modulate and measure anti-tumor immune relevance of H3K36me2/me3 need to be done. This includes: use of pharmacologic approaches in vivo to inhibit NSD2 and/or EZH2 in their autochtonous/orthotopic mouse models to document whether inhibition of H3K36me2 and H3K27me3 impacts expression of endogenous retroviral elements as measured for example by dsRNA, alter expression of type I interferons, affect infiltration of tumors with immune cells and ultimately its impact on tumor burden.

A3.1: We appreciate this important point and agree that the relevance of H3K36 methylation biology to human lung adenocarcinoma should not rest solely on the *$H3^{K36M}$* model. Although *$H3^{K36M}$* mutations are not recurrent in lung adenocarcinoma, we used $H3^{K36M}$ as a genetic tool to broadly inhibit H3K36 methyltransferase activity *in vivo*. This approach allowed us to uncover an unexpected relationship between H3K36 methylation, ERV-derived dsRNA expression, and tumor immune surveillance. Importantly, our conclusions are not based solely on *$H3^{K36M}$* expression. Rather, the *$H3^{K36M}$* model led us to identify NSD2-mediated H3K36me2 as the disease-relevant and therapeutically actionable axis in KRAS-driven lung adenocarcinoma.

To strengthen this point, the revised manuscript includes multiple $H3^{K36M}$-independent approaches. First, genetic inactivation of *Nsd2* in the KRAS-driven autochthonous model phenocopied *$H3^{K36M}$* expression, reducing H3K36me2, inducing dsRNA accumulation, promoting immune infiltration, and suppressing tumor growth (**Fig. 5b-d and Extended Data Fig. 10b-d**). Second, we now show directly that *Nsd2* inactivation increases ERV/repetitive element expression in mouse KP cells, linking NSD2 loss to the proposed ERV-dsRNA mechanism (**Extended Data Fig. 10e-h**). Third, we extended these findings to human lung cancer cell lines using the NSD2 inhibitor KTX-1001, which induced LINE/SINE expression and robust cytoplasmic dsRNA accumulation (**Extended Data Fig. 12a,b**). Thus, the revised manuscript now directly

manipulates NSD2 genetically and pharmacologically and demonstrates effects on repetitive element expression and dsRNA accumulation in both mouse and human lung cancer systems.

We agree that *in vivo* pharmacologic inhibition of NSD2 and/or EZH2 in autochthonous or orthotopic models would be informative. These experiments require optimization of drug exposure, scheduling, pharmacodynamic assessment, and therapeutic combinations and are therefore beyond the scope of the present revision. However, related *in vivo* work has recently shown that KRAS-driven lung adenocarcinoma tumors regress upon combined KRAS and NSD2 inhibition [3]. Our study provides a mechanistic framework that may help explain this therapeutic effect by linking NSD2/H3K36me2 inhibition to repetitive-element derepression, dsRNA accumulation, and immune activation.

We also expanded the translational relevance of the study through human tumor analyses and pharmacologic combination experiments. CIBERSORT analysis of human lung adenocarcinoma datasets showed that low *NSD2* expression is associated with increased immune infiltration, whereas *SETD2* loss-of-function alterations are associated with reduced immune infiltration (**Extended Data Fig. 11a**). In human lung cancer cell lines, NSD2 inhibition induced dsRNA accumulation, SETD2 inhibition attenuated this response, and EZH2 inhibition restored dsRNA accumulation in the combined NSD2i/SETD2i setting, while EZH2 inhibition alone did not induce dsRNA (**Fig. 5e-h and Extended Data Fig. 12c,d).** These findings reinforce the human relevance of the NSD2-SETD2-EZH2 axis and support the idea that SETD2 status may influence responses to NSD2-targeted therapeutic strategies.

We have revised the manuscript to make this distinction clearer: $H3^{K36M}$ is used as a mechanistic discovery tool, whereas NSD2 inhibition and SETD2/EZH2-dependent regulation of ERV-derived dsRNA represent the more directly relevant translational framework for human lung adenocarcinoma.

Q3.2: “The authors state that introduction of the oncohistone H3 K36M substantially reduced the number of tumors observed and the size of each tumor in Fig 1bc. However, they are NOT statistically significant as seen in Fig 1C and Ext Data Fig 1a. This is an important point as the authors conclude that ”… H3K36M suppresses tumor formation and growth in a Setd2-dependent manner…” This might require additional sample size to get appropriate statistical power.”

A3.2: We thank the reviewer for raising this important statistical point. In response, we increased the number of mice in the relevant experimental cohorts to improve statistical power. With the expanded sample size, we now observe statistically significant decreases in both tumor burden and tumor number when comparing *K-Ctrl* tumors with $KH3^{K36M}$*-Ctrl* tumors (**Fig. 1c and Extended Data Fig. 1a**). Thus, the revised data now support the conclusion that $H3^{K36M}$ expression suppresses KRAS-driven tumor formation and growth in a *Setd2*-dependent manner. We have updated the figure panels, statistical analyses, and text accordingly.

Q3.3: “This is further noted in Ext Data Fig. 5D which shows that H3K36M appears to have the same impact as Control on tumor burden. Authors need to show pairwise statistical tests between all the 4 cohorts in this figure. Looking at the data as presented, it is obvious that the

Setd2 ko is the dominant genetic lesion with further introduction of H3K36M having a modest impact, at best, on tumor burden."

A3.3: We thank the reviewer for this comment and have updated **Extended Data Fig. 5d** to include the requested pairwise statistical comparisons among the four cohorts.

We agree that the orthotopic experiment has important interpretive differences from the autochthonous model. In this experiment, the rapid growth of *KP-H3^{K36M}-Setd2^{KO}* tumors required euthanasia at 5 weeks post-injection as a humane endpoint. This timing limited the dynamic range for detecting differences in total tumor burden between *KP-H3^{WT}-Ctrl* and *KP-H3^{K36M}-Ctrl* tumors. Accordingly, the pairwise comparison between *KP-H3^{WT}-Ctrl* and *KP-H3^{K36M}-Ctrl* tumor burden trended in the expected direction but did not reach statistical significance ($p = 0.1493$). However, *KP-H3^{K36M}-Ctrl* mice had markedly fewer tumors than *KP-H3^{WT}-Ctrl* mice, with a mean of 1.6 tumors per mouse compared with 16 tumors per mouse in the *KP-H3^{WT}-Ctrl* group, consistent with impaired engraftment and/or immune-mediated clearance of *H3^{K36M}*-expressing cells.

Importantly, the same biological effect is more clearly captured in the autochthonous model, where tumor initiation and progression occur in the native lung environment and where the endpoint was not dictated by the rapid growth of the *KH3^{K36M}-Setd2^{KO}* transplant cohort. In the expanded autochthonous cohort, *H3^{K36M}* expression significantly reduced both tumor burden and tumor number relative to *K-Ctrl* tumors, whereas *Setd2* inactivation restored tumor formation and growth in the *H3^{K36M}*-expressing background (**Fig. 1c and Extended Data Fig. 1a**). Thus, the autochthonous data provide the primary statistically supported evidence that H3^{K36M} suppresses KRAS-driven tumor formation and growth in a *Setd2*-dependent manner.

We have revised the text to avoid over-interpreting the orthotopic tumor-burden comparison. The orthotopic model provides complementary evidence that *H3^{K36M}*-expressing tumor cells are poorly tolerated in immune-competent hosts, that this phenotype is reversed by *Setd2* inactivation, and that the effect is immune-dependent because *H3^{K36M}*-expressing cells form tumors efficiently in severely immunodeficient NCG mice (**Extended Data Fig. 5c,d and Extended Data Fig. 6a,b**).

Q3.4: "Fig 1d shows modest expression of H3K36M in the control group while it is highly expressed in the Setd2ko. Importantly, this is seen again in Fig 3b (orthotopic model experiment, whereby K36M expression is higher in the Setd2 KO background. What is the cause of this? This raises the question of whether the level of expression of K36M is modulated by Setd2."

A3.4: We thank the reviewer for raising this point. In the autochthonous model, H3^{K36M} is readily detected by immunohistochemistry in both *KH3^{K36M}-Ctrl* and K*H3^{K36M}-Setd2^{KO}* malignant cells (**Fig. 1d**). Thus, in the *in vivo* setting that provides the primary evidence for *H3^{K36M}*-mediated tumor suppression, the difference in phenotype is not explained by a failure of *KH3^{K36M}-Ctrl* tumors to express the *H3^{K36M}* transgene.

We agree that the immunoblot in the KP cell-line system shows higher *H3^{K36M}* signal in *KP-H3^{K36M}-Setd2^{KO}* cells than in *KP-H3^{K36M}-Ctrl* cells (**Fig. 3b**). Although it is possible that *Setd2* status could influence *H3^{K36M}* expression, we do not have evidence that Setd2 directly regulates the *H3^{K36M}* transgene. We think a more likely explanation is

clonal variation and/or selection during generation and expansion of the *KP-H3[K36M]-Ctrl* cell lines. *H3[K36M]*-expressing cells show reduced tolerance in immune-competent settings, and *KP-H3[K36M]-Ctrl* cells show a modest, though not statistically significant, reduction in *in vitro* growth relative to control cells (**Extended Data Fig. 5a**). These factors could select for clones with comparatively lower *H3[K36M]* expression, whereas *Setd2* inactivation may increase tolerance of the *H3[K36M]*-expressing state.

Importantly, despite this difference in expression level in the cell-line model, *KP-H3[K36M]-Ctrl* cells display the expected biological and molecular phenotypes: reduced H3K36 methylation, increased ERV/repetitive element expression, dsRNA accumulation, IFN-β production, and immune-dependent rejection *in vivo* (**Fig. 3b-g, Fig. 4a-g, Extended Data Fig. 5c-e, and Extended Data Fig. 6a,b**). Thus, the central conclusions are not dependent on higher *H3[K36M]* expression in the *Setd2[KO]* background. We have clarified this point in the revised text.

Q3.5: "The authors report in Ext Data Fig 7d that "..Setd2 inactivation had a more subtle effect…" while describing the ChIP-seq results. This is quite in contrast to the H3K36me3 IHC images shown in Fig 1D that showed substantial abolishment of total H3K36Me3 histone mark in the K Setd2 KO tumors. This needs to be investigated further and source of discrepancy resolved as it is a crucial piece of information (with potential differences arising from orthotopic vs autochtonous model difference, IHC vs ChIP-seq assay, antibody difference etc..). As further supporting data, authors need to investigate this in human lung adenocarcinoma cell lines and show difference between SETD2 WT vs mutant cell lines."

A3.5: We thank the reviewer for highlighting this point. We agree that the relationship between the IHC and ChIP-seq data should be clarified. We believe the apparent difference in magnitude reflects both technical and biological differences between the assays. The H3K36me3 IHC in **Fig. 1d** was performed on autochthonous tumors in situ and provides a tissue-level assessment of H3K36me3 staining intensity in tumor sections. In contrast, the ChIP-seq analysis in **Extended Data Fig. 7d** was performed in clonal KP cell lines and reports positional enrichment of H3K36me3 across gene bodies after chromatin immunoprecipitation, sequencing, normalization, and metagene averaging. Thus, IHC and ChIP-seq are not expected to yield identical apparent magnitudes of change.

We have revised the text to avoid overstating this comparison. In the ChIP-seq analysis, our statement that Setd2 inactivation had a "more subtle effect" referred specifically to the metagene profile across coding gene bodies in the KP cell-line system. This does not conflict with the IHC result showing strong reduction of H3K36me3 staining in Setd2-targeted autochthonous tumors (**Fig. 1d**). Rather, these data reflect different experimental contexts and analytical scales: whole-tissue staining in autochthonous tumors versus locus-averaged chromatin enrichment in cultured KP cells.

Importantly, the KP cell-line system faithfully reproduced the central functional relationships observed in the autochthonous model. In orthotopic transplantation experiments, *Setd2* inactivation increased tumor burden and reversed the poor tolerance of *H3[K36M]*-expressing cells in immune-competent mice (**Extended Data Fig. 5c,d**). In the same cell-line system, *H3[K36M]* expression induced ERV/repetitive element expression, dsRNA accumulation, and IFN-β production in a *Setd2*-dependent manner (**Fig. 3g and Fig. 4a-d**). Thus, although the apparent magnitude of H3K36me3 depletion differs

between assays and systems, the KP cells capture the key genetic and functional interactions central to the study.

We also note that previous work from our laboratory demonstrated that SETD2 knockdown in human lung adenocarcinoma cell lines significantly depletes H3K36me3 [1]. We have revised the manuscript to clarify the assay-specific interpretation of the IHC and ChIP-seq data and to avoid implying that these readouts are directly quantitative equivalents.

REVIEWER 4

Major Comments:

Q4.1: “The manuscript presents a compelling "enzymatic triad" model (NSD2, SETD2, and EZH2) that dictates tumor immune surveillance. While the scientific foundation is robust, the current presentation, particularly in the Abstract, is linguistically dense and could more effectively highlight the study’s paradoxical nature to engage a broader readership. I have the following specific suggestions:

The sentence in the Abstract (“Specifically, loss of ... leads to derepression of ... that activates ... that ... that...”) is structurally over-complex due to nested “that” clauses. Coupled with high-density epigenetic jargon, this may hinder the immediate grasp of the causal chain. I suggest decoupling these steps to explicitly highlight the viral mimicry mechanism; for example: "Specifically, loss of H3K36me2/3 (via H3K36M expression or NSD2 inactivation) derepresses endogenous retroviral elements (ERVs). The resulting transcripts form dsRNA, triggering an antiviral-like immune response that potently eradicates KRAS-driven tumors."
The term "ERV" is used later in the abstract (and throughout the text), but its full name, "endogenous retroviral elements," is mentioned without being linked to the abbreviation upon its first occurrence. For the sake of clarity, please introduce the abbreviation immediately following the full term: "endogenous retroviral elements (ERVs)."
The most striking feature of this work is the biological paradox it resolves. However, the Abstract and the end of the Introduction could more sharply delineate the "Knowns" vs. the "Paradox." I suggest the authors restructure these sections to follow a clearer “Known Background -->Paradox --> Mechanistic Solution” template if I get the core idea correctly, e.g.
- The Known: it’s established that H3K36M inhibits the function of SETD2; and SETD2 loss is a potent driver of KRAS-driven LUAD;
- The Paradox: Logic dictates that H3K36M should be oncogenic in this context; however the authors found it tumor-suppressive; and what’s more, this suppression requires SETD2, the enzyme that H3K36M blocks.
- The Answer: H3K36M inhibits H3K36 methylation; SETD2 prevents EZH2 from re-silencing ERVs, hence maintaining immune-mediated tumor killing.”

A4.1: We appreciate this helpful suggestion. We have substantially revised the Abstract to improve readability, reduce nested clauses, and more clearly present the causal sequence linking H3K36me2 loss, endogenous retroviral element derepression, dsRNA accumulation, antiviral-like immune activation, and tumor suppression. We now introduce the abbreviation “endogenous retroviral elements (ERVs)” upon first mention.

We have also revised the end of the Introduction to more clearly frame the known biology, the unexpected observation, and the mechanistic model that emerged from our study. Specifically, we now more explicitly state that *Setd2* loss promotes KRAS-driven

lung adenocarcinoma, whereas *H3*^{K36M} expression, despite inhibiting H3K36 methylation, unexpectedly suppresses tumor growth in a SETD2-dependent manner. We then introduce the NSD2–SETD2–EZH2 axis as the mechanistic solution to this observation.

Q4.2: “The Discussion provides a fascinating mechanistic insight into why H3K36M, traditionally viewed as an oncogenic, acts as a tumor suppressor in KRAS-driven lung adenocarcinoma. To further broaden the impact of this study, I suggest the authors consider adding a brief elaboration on the tissue-specific context of this paradox.

While the authors acknowledge the 'bona fide oncogenic potential' of H3K36M in many other tumor types, the finding that it triggers potent immune-mediated eradication in the lung is particularly striking. Adding a sentence or two to discuss whether this reversal is driven by the unique immune surveillance landscape of the lung—compared to the more 'immune-cold' environments like bone or cartilage where H3K36M is oncogenic, would provide valuable context.”

A4.2: We thank the reviewer for this helpful suggestion. We have added text to the Discussion noting that the consequences of *H3*^{K36M} expression may be strongly shaped by tissue context, including the local immune environment. Specifically, we now discuss the possibility that *H3*^{K36M}-induced dsRNA accumulation is more effectively translated into immune-mediated tumor suppression in the lung, a tissue with robust immune surveillance capacity, whereas other tissue contexts in which *H3*^{K36M} is oncogenic may be less permissive for this type of antiviral-like tumor immune response. We present this as an important context-dependent consideration and future direction rather than as a definitive explanation.

Q4.3: “The current manuscript lacks sufficient technical detail regarding the derivation and calculation of the "Hotspot Modules" and their respective "Module Scores." Please clarify the definition and composition of the modules and the module scoring algorithm in the method parts.”

A4.3: We thank the reviewer for identifying this omission. We have expanded the Methods section to include additional technical detail regarding the Hotspot analysis. Specifically, we now clarify that malignant cells were subsetted for this analysis, spatial positions were used as the similarity graph, and Hotspot was then used to identify genes with spatially autocorrelated expression and group them into modules. We also now describe how module scores were calculated on a single-cell basis by averaging standardized residuals from the DANB model for all genes assigned to each module.

Q4.4: “The manuscript states that Xenium spatial transcriptomics was performed across four experimental groups (K-Ctrl, K-Setd2KO, KH3K36M-Ctrl, KH3K36M-Setd2KO). Please clarify, for each group, how many independent mice were analyzed (and how many lung lobes/sections per mouse), and how these replicates were incorporated into the statistical analyses (e.g., mouse-level aggregation vs. treating tumors/cells as independent observations). And are these replicates sufficient statistically?”

A4.4: We thank the reviewer for raising this important point. We have revised the Methods to clarify the experimental replication and statistical treatment of the Xenium spatial transcriptomics data. The Xenium experiment included 2–3 lung lobes per experimental group, and individual tumors were segmented and analyzed across these samples. Because each tumor in the autochthonous model arises from an independent initiating recombination event, tumor-level analyses were performed using individual tumors as the unit of observation.

However, we recognize that there may be systemic effects caused by the mouse as a whole. Because of this and due to the natural limitations on sample size in spatial transcriptomic experiments, we independently validated these results using independent histologic approaches across larger mouse cohorts. Specifically, we confirmed the major immune-infiltration patterns by IHC for CD45, CD3, CD8, B220, NKp46, Foxp3, CD11c, CD207, F4/80, and Ly6G using at least n = 5 mice per group (**Extended Data Fig. 2b,c**). These orthogonal analyses support the key spatial transcriptomic findings: *$H3^{K36M}$*-expressing tumors exhibit robust immune infiltration, whereas *Setd2* inactivation suppresses this immune response. Thus, while the Xenium analysis provides high-resolution spatial and transcriptional insight, the main immune-composition conclusions are supported by independent validation in larger cohorts.

Q4.5: “The Data Availability statement is unclear regarding deposition of newly generated datasets (e.g., Xenium spatial transcriptomics, and other sequencing-based assays). Please explain why these datasets are not publicly available, and provide accession numbers/links if they have been deposited (e.g., GEO/SRA/ENA or an equivalent repository), or a timeline and mechanism for access if deposition is pending.”

A4.5: We thank the reviewer for raising this important point. We have revised the Data Availability statement to provide reviewer access to all newly generated datasets. All sequencing and spatial transcriptomics datasets generated for this study, including datasets from Xenium spatial transcriptomics, RNA-seq, ATAC-seq, ChIP-seq, and the CRISPR enrichment screen, have been deposited in GEO and will be publicly available upon publication.

Minor Comments:

MC4.1: “Do spatial plot panels and H&E panels in Fig2a share the same scale? The y-axes of different panels in Extended Data Fig. 7a. should be aligned. And the outlier values could be clipped to better present the squeeze genes in the volcano plots.”

A4.1: We thank the reviewer for these careful suggestions. The spatial transcriptomic plots and corresponding H&E panels in **Fig. 2a** are shown at the same scale, and we have now clarified this in the figure legend. We have also revised **Extended Data Fig. 7a** so that the y-axes are aligned across panels. We appreciate the reviewer’s careful attention to figure clarity and presentation.

MC4.2: “In Extended Data Fig. 7b, the enriched gene sets are too blurred. Please use high-resolution figures instead.”

A4.2: We thank the reviewer for noting this issue. We have replaced the **Extended Data Fig. 7b** panel with a higher-resolution version so that the enriched gene-set labels and plotted values are more legible.

MC4.3: “It’s not necessary to arrange genes of 7 modules in tabular format in Supplemental Table 1. Seven lists of genes would make this part more readable.”

A4.3: We thank the reviewer for this helpful formatting suggestion. We have reformatted **Supplemental Table 1** so that the genes assigned to each Hotspot module are presented as separate module-specific lists rather than as a single seven-column table. This revised format should make the module composition easier to read and interpret.

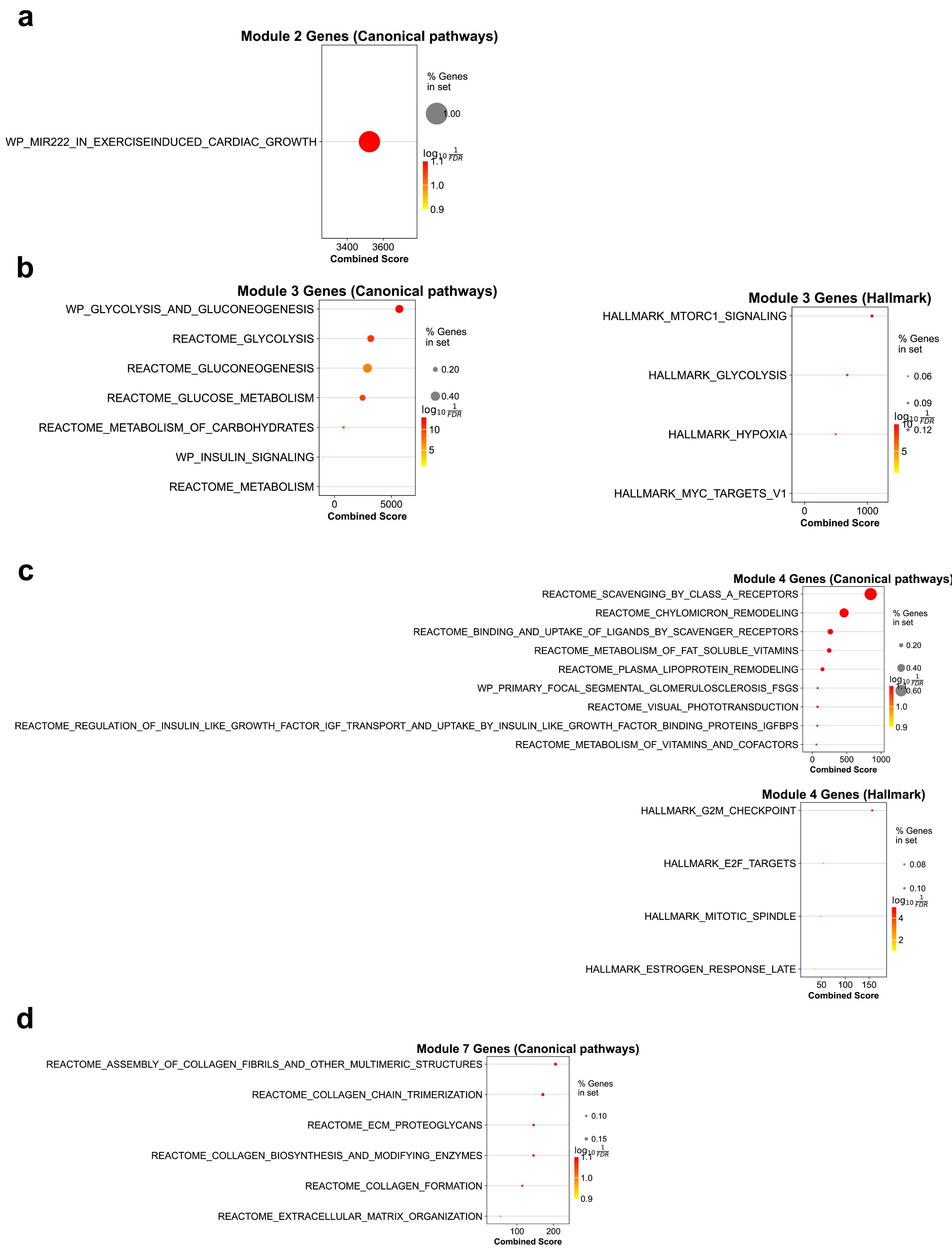

Rebuttal Fig. 1: GO analysis of modules enriched in *KH3^{K36M}-Setd2^{KO}* tumors

Dot plots showing enrichment of Canonical Pathway and Hallmark gene sets for genes contained within **(a)** Module 2, **(b)** Module 3, **(c)** Module 4, and **(d)** Module 7. The x-axis indicates the combined enrichment score. Dot size represents the proportion of genes from each module contained within the indicated pathway, and dot color denotes enrichment significance (–log10 FDR).

a

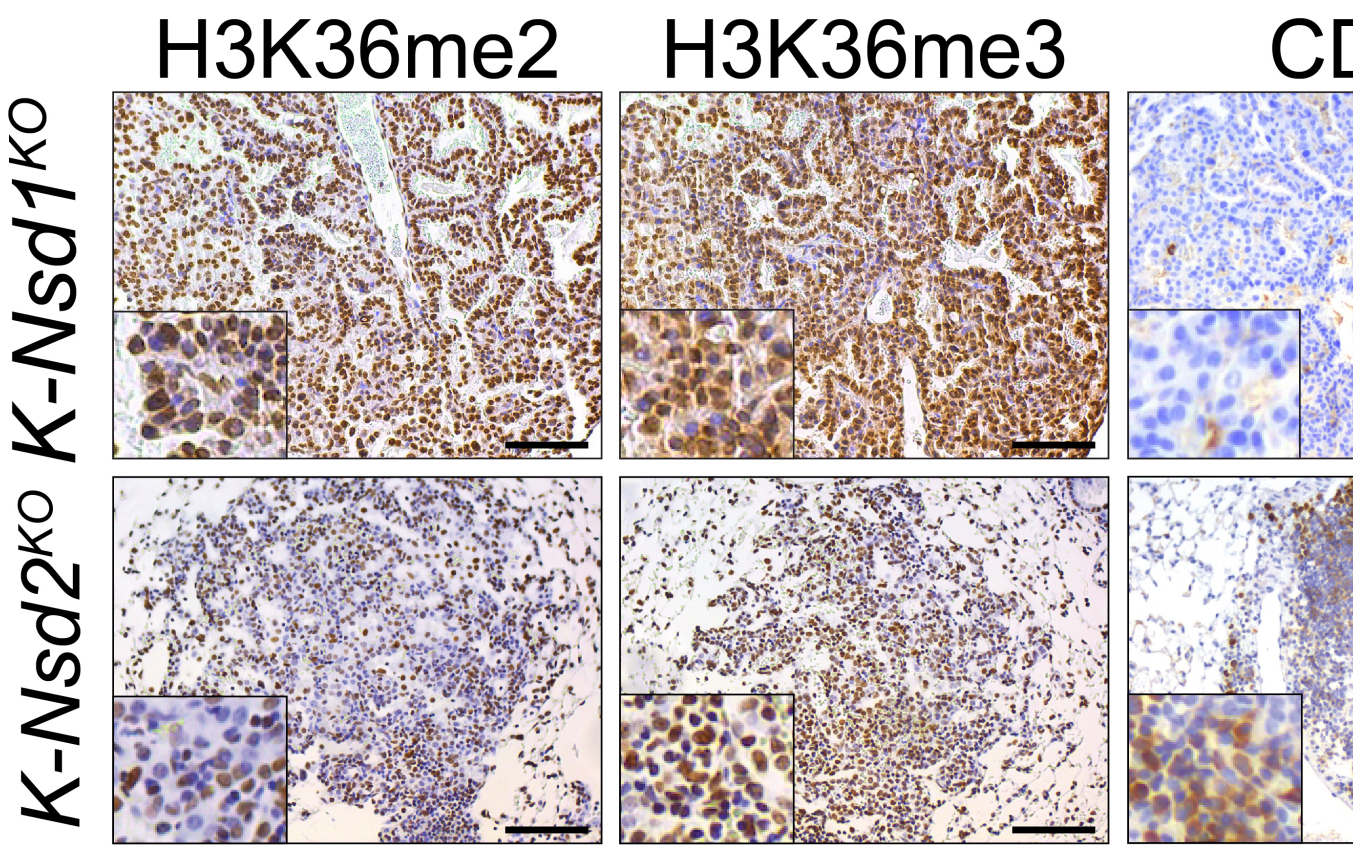

Rebuttal Fig. 2: *K-Nsd1^KO* tumors do not lose H3K36me2
a. IHC for H3K36me2, H3K36me3, and CD45 in *K-Nsd1^KO* and *K-Nsd2^KO* tumors.

Rebuttal References

1. Walter, D.M., et al., *Setd2 inactivation sensitizes lung adenocarcinoma to inhibitors of oxidative respiration and mTORC1 signaling.* Commun Biol, 2023. 6(1): p. 255.
2. Xie, Y., et al., *SETD2 regulates chromatin accessibility and transcription to suppress lung tumorigenesis.* JCI Insight, 2023. 8(4).
3. Jeong, J., et al., *NSD2 inhibitors rewire chromatin to treat lung and pancreatic cancers.* Nature, 2026. 649(8095): p. 205-215.

We have addressed Reviewer #3 comments (replicated below with our responses in blue):

1) I could not locate Extended Data Fig 10f-h that is cited as a supporting evidence regarding nsd2 knockout and ERV and hence can not adequately assess the response to my question #1.
Also, Fig 5h is missing (cited in response to question #1).

We mistakenly cited an old version of our figures in the response to this reviewer comment. To address the first comment, we have shown that Nsd2 knockout increases ERV expression and dsRNA expression in Figures 5e-g, not Extended Data Fig 10f-h. For the second comment, we meant to cite Figures 6a-e, rather than Figures 5e-h. This figure shows that in human lung cancer cell lines, NSD2 inhibition induced dsRNA accumulation, SETD2 inhibition attenuated this response, and EZH2 inhibition restored dsRNA accumulation in the combined NSD2i/SETD2i setting, while EZH2 inhibition alone did not induce dsRNA, addressing the reviewer's request to increase the human relevance of the study.

2) in Ext fig 12d dsRNA image is very dim and can not be assessed. the overlay shows simply the loss of the strong H3K27me3 signal. Better images need to provided.

The take home message of Extended Data Fig 12d is that **no** dsRNA is induced when cells are treated with EZH2 inhibitor. The images in that figure panel are not dim because they are poor quality but because they reflect the biological result.

3) Authors need to show the comparison and non-significant p-value of 0.1493 in ext. fig 5d (from response to question #3)

We have added this p-value to the figure.
